# Supplementary material for: The spatio-temporal patterns and formation mechanisms of cholera epidemics in Hubei Province, China from 1949 to 2020
Source: PLoS Negl Trop Dis. 2026 Mar 18;20(3):e0014125. doi: 10.1371/journal.pntd.0014125 (PMC13020998; doi:10.1371/journal.pntd.0014125)
Supplement: Attachment — Submitted filename: PNTD-D-25-01715_reviewer_report_submitted.pdf [file pntd.0014125.s005.pdf]

Reviewer Report

Manuscript ID: PNTD-D-25-01715
Title: The Spatio-temporal Patterns and Formation Mechanisms of Cholera Epidemics in Hubei Province, China from 1949 to 2020
Journal: PLOS Neglected Tropical Diseases

Overall Assessment

This manuscript presents a comprehensive analysis of cholera epidemics in Hubei Province spanning 72 years (1949-2020). The authors employ multiple analytical approaches—Mann-Kendall trend testing, wavelet analysis, spatial autocorrelation, and structural equation modeling (SEM)—to examine temporal patterns, spatial clustering, and the environmental and socioeconomic drivers of cholera transmission. The long-term dataset and ambitious methodological framework represent significant strengths, and the regional focus on a flood-prone, water-rich area is both timely and relevant for PLOS Neglected Tropical Diseases.

The manuscript makes a solid contribution to understanding cholera dynamics in inland China, particularly in linking hydrological features with disease patterns. However, several areas require clarification and revision before publication, particularly regarding data quality, methodological justification, and interpretation of results.

Recommendation: Major Revision

Major Comments

1. Data Quality and the 25-Year "Cholera-Free Period"

The compilation of historical data from local chronicles is commendable, but critical questions remain about data reliability and completeness:

- The manuscript notes that 71 out of 103 counties reported cholera, with western Hubei (Shiyan, Shennongjia, Enshi) showing almost no cases. Is this truly disease absence or simply poor surveillance and underreporting? While you acknowledge this briefly in the Discussion ("it cannot be ruled out that there are unrecorded epidemic cases"), you don't adequately address how this might bias your spatial analyses, particularly the "east-dense, west-sparse" pattern and the SEM results. This surveillance gap could substantially affect your conclusions about river networks and topography as drivers.
- The 25-year cholera-free period (1955-1979) is fascinating but needs more critical examination. You attribute it to "strict preventive vaccination measures" and cite the 1954 flood response, yet provide no quantitative data on vaccination coverage or sanitation improvements. This period also coincides with major socio-political changes in China that likely affected disease surveillance and reporting. The Cultural Revolution (1966-1976) falls within this period—could reduced reporting, restricted population movement, or healthcare system disruptions have contributed to zero reported cases? You mention the 1954 flood prevention efforts but need to explain why effective vaccination in the 1950s apparently ceased to protect the population in the 1980s when cholera returned with 3,193 cases.
- How were cases defined and diagnosed across different decades? You mention O139 cholera appearing in 1993 and El Tor biotype introduction, but diagnostic capabilities and case definitions surely evolved from 1949 to 2020, potentially affecting case detection rates.

Recommendation: Add a dedicated subsection in the Methods on "Data Quality and Limitations" that discusses surveillance capacity changes by decade. In the Discussion, critically evaluate how surveillance gaps might affect your mechanistic conclusions, particularly for western Hubei.

---

2. Structural Equation Model: Conceptual and Technical Issues

The SEM is ambitious and represents the paper's main analytical contribution, but several concerns need addressing:

Variable Selection and Conceptual Framework:

- Why use "annual mean summer temperature" as a proxy for infection source rather than actual water temperature or seasonal *Vibrio* prevalence data? The conceptual linkage feels indirect. You justify this by stating summer is the high-incidence season, but then your seasonal analysis shows August-September-October peaks, which includes early autumn. This inconsistency needs explanation.
- The exclusion of critical variables is acknowledged in the Discussion but needs upfront acknowledgment as a major limitation:
    - No vaccination coverage data (yet you claim vaccination explains the 1955-1979 gap)
    - No water pH data (acknowledged as affecting *Vibrio* survival)
    - No sanitation infrastructure data
    - Road network density exists for only 6 time points (1962, 1980, 1990, 2000, 2010, 2020) with interpolated values for 66 other years. This temporal mismatch for a 72-year analysis is problematic and needs explicit justification in the Methods section.

Interpretation Issues:

- **River network density** shows a total effect of only 0.013 (Table 3)—essentially zero compared to population density (0.837) and summer temperature (0.807). Yet you state it "contributed to cholera epidemics levels." This coefficient is so weak it's arguably negligible. You should temper claims about river networks being a "driver," perhaps reframing them as a necessary background condition rather than an active direct factor in your model.
- **Night Lights** shows an extraordinarily high coefficient (1.080)—higher even than population density. While you link this to urbanization, such an extreme standardized coefficient can indicate model specification issues or unaccounted multicollinearity despite acceptable VIF values (7.527, though notably high). Night lights likely serves as a composite proxy overlapping substantially with population density (VIF=5.643) and GDP. The indirect effect is negative (-0.059), suggesting suppression effects. This needs careful interpretation—you cannot simply state it "significantly increases cholera" without acknowledging these statistical complexities.
- The **positive effects of GDP and nighttime lights** on cholera seem counterintuitive (wealthier areas should have better sanitation). Your explanation about "population mobility" and "wastewater discharge" feels post-hoc. An alternative explanation—that these variables reflect better disease surveillance and reporting in developed urban areas rather than true elevated risk—deserves serious consideration. Wuhan shows high cholera incidence in your maps and also has the highest GDP and night lights. Is this real elevated risk or better case detection?

Technical Considerations:

- You're essentially analyzing panel data (103 county-level observations across 72 years), but your SEM appears to treat this as cross-sectional data with time-averaged values. Have you considered:
    - Time-lag effects (e.g., does flood in year t affect cholera in year t+1)?
    - Non-stationarity in the time series?

    - Temporal autocorrelation within counties?
- The VIF test (Table 1) shows acceptable values, but you should clarify exactly what data structure was tested—are these 103 spatial observations with time-averaged variables, or something else?
- Table 3 is critical but difficult to interpret. The distinction between "overall," "direct," and "indirect" effects needs clearer explanation in the Methods. Readers unfamiliar with SEM path analysis will struggle to understand what these values represent.

Recommendation:

1. Add a supplementary table with complete variable descriptions: name, definition, unit, temporal coverage, source, and role in model
2. Revise interpretations to be more cautious, particularly for river networks (weak effect) and night lights (potential confounding)
3. Explicitly address the cross-sectional vs. time-series nature of your SEM
4. Consider alternative explanations for the GDP/night lights paradox

---

3. Statistical Rigor and Interpretation of Temporal Patterns

Several statistical findings need stronger justification or clarification:

M-K Test Inconsistency: The text states "a significant downward trend was observed from 1955 to 1994" based on the UF curve (Figure 4). However, this period includes your high-incidence phase (1980-1999) with 88.4% of all cases. How can there be a downward trend during a period of epidemic resurgence? Looking at Figure 3, cases clearly increase from 1980-1988. Please clarify what the M-K statistic is actually showing here—is this an error in interpretation, or are you detecting a trend in variance rather than mean?

Wavelet Analysis - The 55-Year Cycle: You identify four fluctuation cycles: 55, 29, 19, and 8 years, with the 55-year cycle described as the "first main cycle." However:

- Your study span is 72 years with only 31 years of actual epidemic data
- A 55-year cycle can only complete about 1.3 cycles within your timeframe
- You have essentially one peak and one trough to define this "cycle"
- Figure 6 shows the 55-year peak is only marginally higher than the 29-year peak

This is statistically precarious. You cannot reliably detect a cycle that nearly spans your entire observation period. The wavelet analysis is better suited for identifying the 29, 19, and 8-year cycles. I recommend either:

1. Stating explicitly that the 55-year cycle is "tentative" or "preliminary" given data constraints
2. Removing it from the main findings and discussing it as a limitation
3. Providing statistical confidence intervals for the wavelet variance peaks

Contextualization of Cycles: The four cycles need better interpretation beyond mathematical description. Are they linked to:

- Climate oscillations (e.g., ENSO, PDO)?
- Socio-political transitions (policy changes, healthcare reforms)?
- Pathogen evolution (El Tor introduction in 1961, O139 in 1993)?
- Generational immunity cycles?

The Discussion briefly mentions El Tor and O139 but doesn't connect these to your cyclical findings. The 29-year cycle roughly corresponds to the time between the 1960s El Tor introduction and the 1990s peak—is this coincidental or mechanistically related?

Phase Classification: You divide the timeline into four phases (1949-1954, 1955-1979, 1980-1999, 2000-2020) but state "No abrupt change points were detected between the UF and UB curves." If there are no statistical change points, what justifies these exact boundaries? The phase division appears to be based on visual inspection of Figure 3 rather than statistical criteria. Please clarify your methodology for defining these periods.

---

4. Spatial Analysis and Presentation

Missing Visualizations:

- You mention local spatial autocorrelation analysis and state results about "High-High and Low-Low clusters" in the text, but Figure 8b only shows overall Moran's I results. Please add maps showing the LISA (Local Indicators of Spatial Association) results with High-High, Low-Low, High-Low, and Low-High clusters clearly marked for different time periods.

Center of Gravity Analysis:

- The shift from northeast to southwest is interesting and well-presented in Figure 9f. However, you don't adequately explain *why* this shift occurred. What changed in these regions? You mention that southeastern Hubei (Xianning) became a hotspot, but was this due to:
  - Population growth and urbanization?
  - Changes in water management or sanitation?
  - Economic development patterns?
  - Proximity to new transportation routes?

Seasonal Distribution Paradox: You state cholera peaked in "August, September, and October" with August (31.56%), September (33.05%), and October (15.29%) being highest. October is technically autumn, not summer. Yet you use "annual mean summer temperature" (June-August) as your SEM variable. There's a temporal mismatch between when cholera actually peaks (Aug-Oct) and your temperature proxy (Jun-Aug). This needs explanation—is there a 1-month lag effect, or should you be using a different seasonal temperature variable?

Minor Comments

Abstract and Author Summary

- **Abstract - Future Tense:** Change "This study will help to reveal" to "This study reveals" or "Our findings fill" (past tense is appropriate for completed research)
- **Inconsistency between Abstract and Author Summary:** The Author Summary states floods and topography played "indirect roles," while the main Abstract simply lists them as factors. This distinction between direct/indirect effects is a key finding from your SEM and should be consistent across both summaries.

Introduction

- The historical references to "Tiger Lera" and "Tiger Epidemic" are interesting cultural context but feel tangential to your analysis. Consider shortening or moving to a footnote.
- You cite extensive work on cholera in other regions but should strengthen the justification for why Hubei specifically matters. You mention it's a "province of a thousand lakes" but could emphasize: (1) Hubei's position as a major transportation hub, (2) the Three Gorges Dam's impact on hydrology, (3) the 1998 Yangtze flood as a natural experiment, or (4) applicability to other monsoonal regions with dense river networks.

Methods Section

Spatial Merging Method: You describe merging historical counties to match 2020 boundaries, which is excellent practice. However, you need more detail:

- When counties split, how did you allocate historical case counts? Population-weighted? Area-weighted? Equal division?
- Appendix Table 2 lists boundary changes but doesn't explain your allocation method
- This is crucial for reproducibility

Wavelet Analysis:

- Why was the Morlet wavelet chosen over other mother wavelets (e.g., Daubechies, Mexican Hat)? Provide brief justification.
- What was the scale range used? How many decomposition levels?
- Figure 5's caption mentions "Real contour plot" but doesn't explain how to interpret the colors—what do the alternating red/blue zones represent in practical terms?

Spatial Weight Matrix:

- For spatial autocorrelation (Equation 4), you define W_ij as "the spatial weight of counties i and j" but never specify: Queen contiguity? Rook? Distance-based? First-order neighbors only? Please clarify.

SEM Goodness of Fit:

- Table 2 shows excellent fit indices, but with 103 observations and 11 variables, your sample size is relatively small for SEM (typically want $n>200$). Acknowledge this limitation.

Results Section

Figure Quality and Clarity:

- Figure 3: The 1988 peak (1,351 cases) dominates the y-axis scale, making other years difficult to distinguish. Consider a log scale, broken axis, or an inset zoom for 1949-1987 and 1989-2020.
- Figure 5 (wavelet contours): Font sizes for axis labels are quite small. Ensure these will be legible in print.
- Figure 8: Use consistent color schemes. The graduated color in 8a (case counts) differs from 8b (Moran's I), which can confuse readers.
- Figure 9: Excellent time-series maps, but consider adding the migration path of the center of gravity as a line with arrows directly on panel (f) for easier visualization.
- Figure 10: The path diagram would benefit from including path coefficient values directly on the arrows, not just in Table 3.

Endogenous vs. Exogenous Variables Results: The results section jumps between describing endogenous variables (population density, temperature, river networks) and exogenous variables (elevation, precipitation, floods, etc.) in Table 3. Consider reorganizing with clear subheadings: "Direct Effects of Endogenous Variables" and "Indirect Effects of Exogenous Variables" to improve readability.

Discussion Section

Repetition of Results: The Discussion extensively repeats results already presented. For example, the first paragraph of "Summary of cholera epidemics formation mechanisms" largely restates Table 3 findings. The Discussion should focus on *interpretation* and *contextualization* rather than repetition. Consider:

- Comparing your findings to global cholera research
- Discussing implications for climate change
- Addressing why your findings might differ from coastal cholera patterns

Global Cholera Context: You cite some international studies but could strengthen connections to:

- *Vibrio cholerae* ecology and environmental survival (temperature, salinity, pH)
- Climate-driven cholera outbreaks in Bangladesh, Africa, and elsewhere
- The global shift from water-borne to food-borne transmission
- El Niño-Southern Oscillation (ENSO) effects on cholera globally

The "Double Holiday" Hypothesis: The discussion of Mid-Autumn Festival and National Day (September-October) as cholera risk periods is interesting but speculative. You state "cholera epidemic peaks in Hubei Province are significantly associated with meal gatherings during the Mid-Autumn Festival and National Day holidays," but you haven't shown data demonstrating peaks specifically during these holidays versus other September-October periods. Either provide temporal data showing holiday-specific clustering or tone down this claim.

Policy Recommendations: Your Hubei-specific recommendations are creative and locally relevant:

- Sluice biosurveillance stations at Yangtze/Han River entry points
- "Cholera prevention ecological corridors"
- "Double-Non" certification for restaurants

However, these need feasibility consideration:

- Biosurveillance stations require significant infrastructure and ongoing maintenance—are they cost-effective given the declining cholera incidence?
- How would "ecological corridors" be implemented practically?
- The restaurant certification idea is excellent but needs integration with existing food safety regulations

Consider adding a brief sentence on implementation challenges or resource requirements.

Climate Change Implications: You mention climate warming in the Introduction and use temperature as a key variable, but never discuss future implications. With climate change potentially increasing flooding frequency and altering summer temperatures in Hubei, what are the implications for future cholera risk? This deserves at least a brief paragraph.

Writing and Language

Overall Quality: The English is generally good but needs light editing for grammar, flow, and conciseness. Some sentences are overly long or read as literal translations from Chinese.

Specific Examples:

- Abstract: "The rapid spread and high lethality of the disease not only cause large numbers of deaths and injuries, but also have far-reaching effects by disrupting production, increasing the burden of health care and impeding economic exchanges." → This is overly dramatic for an abstract. Simplify.
- Methods: Many sentences exceed 40 words (e.g., the spatial merging method paragraph). Break these into shorter, clearer sentences.
- Results: "Temporally, high incidence occurred in the 1980s and 1990s, with summer and fall being the dominant seasons." → Use "autumn" instead of "fall" for consistency with international readership.

Terminology Consistency:

- Use "Hubei Province" consistently (avoid mixing "in Hubei," "of Hubei," etc.)
- "Cholera epidemics" vs. "cholera epidemic"—ensure singular/plural consistency
- "O139 cholera" appears in the Discussion without prior definition—define it when first mentioned

Technical Terms:

- Define "El Tor biotype" when first mentioned (Introduction or early Discussion)
- Explain "O139 cholera" for non-specialist readers
- The term "Tiger Lera" is interesting but unexplained—is "Lera" a transliteration of cholera? Clarify.

Tables and Figures

Table 1 (VIF):

- The checkmarks in the "criteria" column are unnecessary in a scientific paper. Just state in the table caption: "All VIF values <10 and tolerance >0.1, indicating acceptable multicollinearity."

Table 2 (SEM Fit):

- Excellent presentation. No changes needed.

Table 3 (SEM Effects): This is your most important table but has some issues:

- The asterisk notation (*, **, ***) is good, but add it to the caption, not just the note
- Consider bolding significant values for easier identification
- The "overall," "direct," and "indirect" terminology needs explanation in the table caption for readers unfamiliar with SEM
- Some readers might prefer separate tables for endogenous and exogenous variables

Figure 2 (Theoretical Framework):

- Good conceptual diagram, but it doesn't show expected direction of effects (positive/negative)
- Consider using + and - symbols on arrows to indicate hypothesized relationships
- This would help readers follow your logic before seeing results

Figure 11: This comparison figure is excellent and crucial for understanding your "Summary of cholera epidemics formation mechanisms." However, it appears very late (page 22) after the Discussion has already begun. Consider moving it earlier or referencing it more prominently in the Results section when discussing temporal patterns.

Specific Data and Methodology Questions

1. **Vaccination Data:** You state vaccination explains the 1955-1979 cholera-free period. Do you have *any* quantitative data on vaccination coverage rates from Chinese public health records, even if incomplete? If so, why wasn't it included in the SEM?
2. **1954 vs. 1998 Floods:** You mention both floods—1954 (no cholera) and 1998 (204 cases). This is a natural experiment. Could you quantify flood severity (e.g., flooded area, population affected) and include it in your analysis? This would strengthen your flood disaster findings.
3. **Time-Series Nature of Data:** Your raw data table shows annual counts from 1949-2020. How exactly did you aggregate this for the SEM? Did you:
    - Use all 72 years × 103 counties = 7,416 observations?
    - Time-average each county's 72 years into one observation (n=103)?
    - Use only epidemic years?

    This needs explicit clarification in the Methods.

4. **Separate Pre-1980 and Post-1980 Analyses:** Given the dramatic differences in surveillance, El Tor introduction, and socioeconomic development, have you considered running separate SEMs for 1949-1979 vs. 1980-2020? This might reveal different mechanisms in different eras.
5. **Model Validation:** Have you validated your SEM by testing whether it can predict the 2000-2020 period using only pre-2000 data? This would strengthen confidence in the identified mechanisms.

Data Availability

You state "All data underlying the findings described in this manuscript are publicly available without restriction." However:

- Given the extensive historical data compilation from local chronicles (Appendix 1 lists dozens of sources), it would be helpful to deposit your compiled dataset in a repository (e.g., Figshare, Dryad, or Zenodo) with a DOI
- Specify exact URLs for the environmental data sources (you cite the National Tibetan Plateau Data Center multiple times—include direct links)
- For reproducibility, consider providing your SEM code (SPSS/Amos syntax) as supplementary material

---

Ethical Considerations

No ethical concerns identified. The study uses historical aggregated data with no individual patient information. Complies with PLOS guidelines.

Summary and Recommendation: Major Revision Required

This manuscript represents a valuable and ambitious contribution to understanding cholera epidemiology in inland China. The 72-year scope is impressive, the historical data compilation is commendable, and the multi-method approach is generally appropriate. Your findings about the "east-dense, west-sparse" pattern,

the southwestward shift of epidemic centers, and the multi-factorial formation mechanisms provide important insights for public health planning in water-rich regions.

However, the paper requires major revisions to address:

1. **Critical evaluation of data limitations**, particularly the cholera-free period, western Hubei surveillance gaps, and how these affect mechanistic conclusions
2. **SEM interpretation and justification**, including:
    - More cautious language about weak effects (river networks)
    - Critical discussion of counterintuitive findings (GDP, night lights)
    - Clear explanation of data structure and temporal aggregation
    - Acknowledgment of missing variables (vaccination, pH, sanitation)
3. **Statistical rigor**, particularly:
    - Clarification of M-K test interpretation
    - Caution about the 55-year cycle given data constraints
    - Justification for phase boundaries
4. **Enhanced spatial analysis visualization** with LISA cluster maps
5. **Contextualization** within global cholera research and climate change implications
6. **Methodological transparency** with complete variable descriptions and clearer explanations
7. **Discussion streamlining** to reduce repetition and strengthen interpretation
8. **Minor language editing** for clarity and conciseness

Specific Required Actions:

- Add "Data Quality and Limitations" subsection in Methods
- Revise SEM interpretation throughout (particularly river networks, night lights, GDP)
- Add supplementary table with complete variable specifications
- Clarify M-K test results and phase boundary justification
- Add LISA cluster maps for local spatial autocorrelation
- Explain seasonal temperature variable vs. actual peak months
- Reduce Discussion repetition; add global context and climate change implications
- Provide data availability statement with repository links or DOIs

Once these revisions are made, I believe this paper will make a strong contribution to PLOS Neglected Tropical Diseases and provide valuable insights for cholera prevention in regions with dense river and lake networks.

Confidential Comments to Editor

The authors have clearly invested considerable effort in compiling 72 years of historical cholera data from dozens of local Chinese sources—a significant undertaking that represents genuine scholarly contribution. The ambition to use structural equation modeling across such a long time span is commendable.

However, the manuscript would benefit substantially from:

1. More critical self-reflection on data limitations (the 25-year zero-case period and western Hubei surveillance gaps are not adequately interrogated)
2. More cautious interpretation of SEM results (some coefficients are presented as meaningful when they're negligible or problematic)
3. Better integration with global cholera literature
4. Clearer methodological exposition for international readers

The core findings about spatial patterns and the multi-factorial nature of cholera epidemics are sound and valuable. The work is fundamentally publishable but needs the revisions outlined above to meet PLOS NTD's standards for methodological rigor and interpretation. I recommend major revision rather than rejection because the underlying research is strong—it primarily needs better presentation and more nuanced discussion of limitations and findings.

The authors should be commended for tackling a neglected regional context and for their innovative use of nighttime lights and other proxy variables when direct measures were unavailable. With careful revision, this will be an important addition to the health geography literature.
