# Supplementary material for: The spatio-temporal patterns and formation mechanisms of cholera epidemics in Hubei Province, China from 1949 to 2020
Source: PLoS Negl Trop Dis. 2026 Mar 18;20(3):e0014125. doi: 10.1371/journal.pntd.0014125 (PMC13020998; doi:10.1371/journal.pntd.0014125)
Supplement: Attachment — Submitted filename: PNTD-D-25-01715_R1_reviewer_report_submitted.pdf [file pntd.0014125.s007.pdf]

Reviewer Report – R1-Revised Manuscript PNTD-D-25-01715R1

Manuscript Title: *The Spatio-temporal Patterns and Formation Mechanisms of Cholera Epidemics in Hubei Province, China from 1949 to 2020*
Journal: *PLOS Neglected Tropical Diseases*

Overall Assessment

I commend the authors for the substantial and thoughtful revisions made to this manuscript. The majority of my previous concerns have been addressed comprehensively, particularly those related to data quality, methodological justification, and interpretation of statistical findings. Overall, the manuscript has improved markedly in clarity, transparency, and scientific rigor. The newly added discussions significantly strengthen the paper's contribution to understanding cholera epidemiology in inland, water-rich regions.

Recommendation: Minor Revision

The manuscript is now very close to being suitable for publication. Only a small number of remaining issues require clarification before final acceptance.

Major Comments

1. Data Quality and Limitations — *Substantially Addressed*

The authors have made excellent progress in this area.

Strengths of the revision include:

- The newly added *Data Quality and Limitations* subsection (Methods, pp. 10–11), which clearly outlines the historical evolution of surveillance systems.
- The expanded *Research Limitations* section (Discussion, pp. 34–36), which critically evaluates how surveillance gaps may influence spatial interpretations.
- The careful recharacterization of western Hubei as a "low-reporting area based on existing data," rather than definitively a "low-risk area," demonstrating appropriate scientific caution.

Remaining minor concern:

While surveillance limitations are now clearly acknowledged in the Methods and Discussion, the Abstract and Conclusions still present some findings in definitive terms (e.g., "71 counties and

districts were cumulatively affected"). To ensure consistency with the nuanced discussion elsewhere, I suggest adding brief qualifying language such as "based on available records" in the Abstract and Conclusions.

---

2. Structural Equation Model (SEM) — *Well Addressed with One Remaining Clarification*

The revisions to the SEM framework are substantial and largely resolve my previous concerns.

Variable selection and conceptual framework:

- The new Table 1 (pp. 7–10) is an excellent addition and provides comprehensive documentation of all variables.
- The rationale for using annual mean summer temperature is now clearly explained and scientifically sound.
- The absence of certain variables (e.g., vaccination, pH, sanitation) is explicitly acknowledged and appropriately contextualized.

Interpretation improvements:

- Reframing river network density as a "geographical background condition" rather than a primary driver is appropriate given its weak coefficient (0.013).
- The reinterpretation of nighttime lights as a composite indicator reflecting both risk and surveillance capacity demonstrates a sophisticated understanding of potential biases.
- The discussion of the apparent GDP/nighttime lights paradox now appropriately considers both real risk amplification and reporting bias.

Technical clarifications:

- The use of time-averaged, cross-sectional data (n = 103) is now clearly stated.
- The small sample size limitation is acknowledged transparently (p. 35).

One remaining issue requiring clarification:

The manuscript states that data were "aggregated along the temporal dimension," with cholera burden represented as cumulative case counts (p. 7). However, for time-varying exogenous variables such as GDP and nighttime lights, multi-year averages were used across the entire 1949–2020 period. This raises a concern about temporal alignment, given that cholera cases were heavily concentrated during the 1980s–1990s.

Specifically, averaging socioeconomic indicators across 72 years may obscure the actual conditions prevailing during peak incidence periods. For example, high GDP values in 2020 may dilute much lower GDP levels during the 1980s, when most cases occurred.

Suggestion:
Please add a brief explanation (2–3 sentences) in the Methods justifying why time-averaging is

appropriate in this context (e.g., identifying long-term structural predispositions rather than short-term triggers), or explicitly acknowledge this temporal mismatch as a limitation.

---

3. Statistical Rigor and Temporal Patterns — *Excellently Addressed*

- The revised explanation of the M–K test results (pp. 15–16) is now clear and correct.
- The removal of the unsupported 55-year wavelet cycle from the main results was an appropriate decision. Focusing on the statistically robust 29-, 19-, and 8-year cycles strengthens the analysis.
- The updated discussion linking cyclical patterns to large-scale climate variability and pathogen evolution adds valuable mechanistic insight.
- Clarification that the four epidemic phases are descriptive rather than statistically detected breakpoints resolves my earlier concern.

No further issues remain in this section.

---

4. Spatial Analysis — *Well Addressed*

- The explanation of the LISA cluster map (Fig. 8b) is now clear and precise.
- The discussion linking the southwestward shift in the center of gravity to changes in regional socioeconomic centers (Xiaogan → Wuhan → Jingzhou/Jiangling) provides a convincing mechanistic narrative.
- The justification for using June–August temperature to explain August–October case peaks is scientifically sound and well-articulated.

No remaining concerns.

Minor Comments

Abstract and Author Summary — *Fully Addressed*

Both sections now consistently use past tense and clearly distinguish direct and indirect effects. Excellent.

Introduction — *Improved*

- Removal of the term "Tiger Lera" improves clarity.
- The expanded rationale for focusing on Hubei Province is much stronger.
- The inclusion of three explicit research questions provides helpful structure.

Minor suggestion:
When introducing "Class A statutory infectious diseases," consider briefly defining this term for international readers (e.g., "the highest threat category in China's infectious disease classification system").

Methods — *Substantially Improved*

- The detailed explanation of spatial merging procedures is clear and reproducible.
- The wavelet analysis methodology is now well justified.
- Clarification of the spatial weight matrix and SEM sample size limitations is sufficient.

Results — *Significantly Improved*

Figures:

- The redesigned Figure 3 with inset panels is particularly effective.
- Improved font sizes, color consistency, and labeling across figures greatly enhance readability.

Tables:

- Table 2 is cleaner and easier to interpret.
- Table 4 benefits from bolded significant values and clearer effect decomposition explanations.

Minor issue:
In Table 4, please clarify whether reported p-values are one-tailed or two-tailed (likely two-tailed).

Discussion — *Significantly Strengthened*

The restructured Discussion is much clearer, particularly with the addition of distinct sections on global comparison, policy implications, and limitations.

- The comparison with Bangladesh and African Great Lakes regions appropriately situates the findings globally.
- The expanded discussion on climate change and early warning systems strengthens future relevance.
- Emphasizing water, sewage, and food management over vaccination for the 1955–1979 control period better reflects historical evidence.

One remaining concern:
Reference 52 (local chronicles) is a valuable historical source, but for a key claim regarding national cholera control strategy, I recommend supplementing it with a peer-reviewed publication on China's cholera control history to strengthen credibility for international readers.

Writing and Language — *Improved with Minor Corrections Needed*

Only a few minor language issues remain:

1. Abstract (p. 2): "has led" → "have led"
2. Introduction (p. 3): Consider briefly explaining why the El Tor biotype mattered (e.g., environmental resilience).
3. Methods (p. 10): Clarify whether the <1% underreporting rate refers to urban areas or a national average.
4. Discussion (p. 28): Adjust tense consistency ("has historically relied").

Outstanding Issues Requiring Attention

1. **[Critical]** Clarify or acknowledge limitations of temporal aggregation in the SEM.
2. **[Minor]** Add qualifying language in the Abstract/Conclusions.
3. **[Minor]** Specify whether Table 4 p-values are one- or two-tailed.
4. **[Minor]** Supplement Reference 52 with a peer-reviewed source.
5. **[Minor]** Address remaining language corrections.

Summary

This is a substantially improved manuscript, and the authors have clearly engaged seriously with the review process. The additions related to data transparency, methodological justification, global context, and climate implications significantly strengthen the paper.

The sole critical remaining issue concerns clarification of the temporal aggregation approach in the SEM. Once addressed, I believe the manuscript will be suitable for publication.

Congratulations to the authors on a thoughtful and rigorous revision. This study will be a valuable contribution to the literature on cholera epidemiology in inland water-rich regions.

Recommendation: Minor Revision

Required before acceptance:

- Clarification of temporal aggregation methodology in the SEM

Recommended but not required:

- Minor clarifications and language refinements as outlined above

---

Confidential Comments to the Editor

The authors have made substantial improvements in response to prior reviews. The remaining issue is methodological but straightforward to resolve. With this clarification, the manuscript will represent a strong and meaningful contribution to *PLOS Neglected Tropical Diseases*, particularly given the rarity and value of a 72-year longitudinal dataset from an inland region.
