# Supplementary material for: The spatio-temporal patterns and formation mechanisms of cholera epidemics in Hubei Province, China from 1949 to 2020
Source: PLoS Negl Trop Dis. 2026 Mar 18;20(3):e0014125. doi: 10.1371/journal.pntd.0014125 (PMC13020998; doi:10.1371/journal.pntd.0014125)
Supplement: Attachment — Submitted filename: PNTD-D-25-01715_R2_reviewer_report_submitted.pdf [file pntd.0014125.s009.pdf]

Reviewer Report – R2 Revised Manuscript

PNTD-D-25-01715R2

Manuscript Title: The Spatio-temporal Patterns and Formation Mechanisms of Cholera Epidemics in Hubei Province, China from 1949 to 2020

Journal: PLOS Neglected Tropical Diseases

Overall Assessment

I have now reviewed this manuscript through two rounds and am pleased to note that the authors have again responded diligently to the concerns raised. The revisions made for this second submission (R2) directly and satisfactorily address the outstanding issues I flagged in my R1 report. The additions are precise, appropriately placed, and do not introduce new inconsistencies into the text. I am satisfied that the manuscript has now reached a level of scientific rigor and transparency appropriate for publication in PLOS Neglected Tropical Diseases.

Recommendation: Accept (pending minor language check)

Response to Outstanding Issues from R1 Report

1. Temporal Aggregation in the SEM — Now Addressed

This was my primary remaining concern from R1. I had flagged the potential mismatch between cholera burden (cumulative case counts, heavily concentrated in the 1980s–1990s) and time-varying exogenous variables like GDP and nighttime lights, which were averaged across the full 72-year study period. The authors have now added a clear, well-reasoned explanation in the Methods section (Structural Equation Modeling subsection, p. 14) justifying the time-averaging approach. The added sentences frame the SEM explicitly as a tool for identifying steady-state spatial drivers rather than short-term volatility triggers, and note that the cross-sectional design aligns with SEM's application premise for detecting long-term structural influences.

I find this justification adequate. While a methodological purist might still prefer period-matched averages (e.g., restricting exogenous variable averaging to 1980–2000 to align with peak incidence), I accept that the authors' framing is internally consistent and is now explicitly stated — which was the core of my concern. The transparency is there; readers can now evaluate this design choice for themselves. No further action needed on this point.

2. Qualifying Language in Abstract and Conclusions — Addressed

I had recommended adding phrases such as "based on available records" to the Abstract and Conclusions to align them with the more cautious language used in the Methods and Discussion. The authors have done this. The Abstract now includes the qualifier "Based on existing historical monitoring records" before summarizing the results, and the Conclusions section has been updated with "Based on existing data" in the spatial distribution paragraph.

This is exactly what I had requested and it reads naturally without disrupting the flow of the text. The consistency across sections is now satisfactory.

3. Table 4 — One-Tailed vs. Two-Tailed p-values — Addressed

The table note has been updated to explicitly state that all significance tests are two-tailed. This is a small but important clarification for methodological reproducibility, and I am satisfied with how it has been handled.

4. Reference 52 — Supplemented with Peer-Reviewed Source — Addressed

I had noted that the key claim about China's national cholera control strategy was supported only by local chronicles (Reference 52). The authors have now added a peer-reviewed citation — Pang B, Kan B. (2021), Disease Surveillance — to the relevant paragraph in the Discussion. This is an appropriate addition and meaningfully strengthens the credibility of the historical argument for international readers.

5. Language Corrections — Addressed

All four minor language corrections I requested have been made. Specifically:

- "has led" → "have led" in the Abstract has been corrected.
- The El Tor biotype now includes a brief parenthetical describing its environmental resilience — this is helpful context for non-specialist readers.
- The <1% underreporting rate is now clarified as a national-level figure.
- Tense has been corrected to "has historically relied" in the Discussion.

6. Class A Infectious Disease Definition — Addressed

I had suggested briefly defining "Class A statutory infectious diseases" for international readers. The authors have added the parenthetical "(the highest hazard level in China's infectious disease classification system)" immediately after the term in the Introduction. This is concise and appropriate — exactly what was needed.

Additional Observations on the R2 Manuscript

Having re-read the full manuscript in its R2 form, I note the following:

Positive observations:

The Discussion section is now genuinely one of the stronger parts of the manuscript. The comparison with cholera dynamics in Bangladesh and the African Great Lakes region is well-integrated and provides real added value, not just token contextualization. The "hydrological base + socio-climatic amplifier" framing is a useful conceptual contribution that should resonate with readers working on cholera in freshwater-dominated settings beyond China.

The policy recommendations section, while unusually detailed for a research article, is internally consistent with the study's findings and the reorganization (placing drinking water safety first) reflects sound public health prioritization.

The methodological decision to retain the full 1949–2020 study period for the Mann-Kendall, wavelet, and SEM analyses — rather than restricting to recent decades as some reviewers suggested — is defensible and the authors' rationale is clearly articulated.

Minor residual language issues (not blocking):

The manuscript continues to carry a small number of grammatical imperfections that appear to reflect non-native English authorship. These are distributed across the Discussion in particular — for example, "indicateed" (p. 32), "provideed" (p. 32), "transcended" (p. 33), and "addtion" (p. 33). None of these affect scientific meaning or reproducibility, but I would recommend a final copy-editing pass before publication to address them. This should not require another full review cycle — the journal's editorial team can handle this at the production stage.

Final Recommendation

All critical and minor concerns raised in my previous reports have been addressed. The authors have engaged seriously and specifically with the review process across both rounds, and the manuscript is substantially better for it. The 72-year longitudinal dataset, the explicit decomposition of direct and indirect drivers through SEM, and the careful situating of findings within the global cholera literature together make this a meaningful contribution to the field.

I recommend acceptance subject to a final language/copy-editing pass at the production stage. No further scientific revisions are required from the authors.

Recommendation: Accept

Required before acceptance: Final copy-editing pass (production stage — no author action needed)

Outstanding scientific concerns: None

Confidential Comments to the Editor

The authors have been thorough and responsive through both review rounds. The remaining language issues are minor and can be addressed at the copy-editing stage without requiring author re-submission. I see no reason to delay acceptance further. This paper fills a genuine gap in the regional cholera literature, and the 72-year dataset is a rare and valuable resource — the analytical work done here is appropriate to the data and the conclusions are well-supported.
