# Supplementary material for: The spatio-temporal patterns and formation mechanisms of cholera epidemics in Hubei Province, China from 1949 to 2020
Source: PLoS Negl Trop Dis. 2026 Mar 18;20(3):e0014125. doi: 10.1371/journal.pntd.0014125 (PMC13020998; doi:10.1371/journal.pntd.0014125)
Supplement: Attachment — Submitted filename: Response to Reviewers.docx [file pntd.0014125.s008.docx]

Dear Editor-in-Chief,

Thank you sincerely for the effective efforts you have devoted to handling our manuscript, and thank the reviewers for their review of our paper and their valuable comments. In accordance with your and the reviewers' comments, we have carefully revised the manuscript. We are now submitting the following explanation of the revisions for your further guidance.

Sincerely yours,

The Authors

December 28, 2025

**The Spatio-temporal Patterns and Formation Mechanisms of Cholera Epidemics in Hubei Province** **, China from 1949 to 2020**

**Response to Reviewers**

**Major Comments**

**Comment 1-1.**

1.Data Quality and the 25-Year "Cholera-Free Period"

The compilation of historical data from local chronicles is commendable, but critical questions remain about data reliability and completeness:

The manuscript notes that 71 out of 103 counties reported cholera, with western Hubei (Shiyan, Shennongjia, Enshi) showing almost no cases. Is this truly disease absence or simply poor surveillance and underreporting? While you acknowledge this briefly in the Discussion ("it cannot be ruled out that there are unrecorded epidemic cases"), you don't adequately address how this might bias your spatial analyses, particularly the "east-dense, west-sparse" pattern and the SEM results. This surveillance gap could substantially affect your conclusions about river networks and topography as drivers.

The 25-year cholera-free period (1955-1979) is fascinating but needs more critical examination. You attribute it to "strict preventive vaccination measures" and cite the 1954 flood response, yet provide no quantitative data on vaccination coverage or sanitation improvements. This period also coincides with major socio-political changes in China that likely affected disease surveillance and reporting. The Cultural Revolution (1966-1976) falls within this period—could reduced reporting, restricted population movement, or healthcare system disruptions have contributed to zero reported cases? You mention the 1954 flood prevention efforts but need to explain why effective vaccination in the 1950s apparently ceased to protect the population in the 1980s when cholera returned with 3,193 cases.

How were cases defined and diagnosed across different decades? You mention O139 cholera appearing in 1993 and El Tor biotype introduction, but diagnostic capabilities and case definitions surely evolved from 1949 to 2020, potentially affecting case detection rates.

Recommendation: Add a dedicated subsection in the Methods on "Data Quality and Limitations" that discusses surveillance capacity changes by decade. In the Discussion, critically evaluate how surveillance gaps might affect your mechanistic conclusions, particularly for western Hubei.

**Response 1-1.**

We sincerely appreciate your valuable comments on the data quality and interpretation of this manuscript. The set of questions you raised are extremely insightful and profound. Below, we will address each of your comments and suggestions individually, outlining specific modification approaches and response plans:

1.You have pointed out the critical issue of how to scientifically distinguish between true absence of cases and data gaps caused by insufficient monitoring—a problem that is particularly salient in the analysis of regions with almost no reported cholera cases, such as western Hubei Province(including Shiyan, Shennongjia, and Enshi). In fact, this constitutes a universal challenge that all studies conducting spatial epidemiological analyses based on historical passive surveillance data must confront and address with prudence.We fully concur with your assessment that surveillance gaps are highly likely to introduce systematic bias into this study’s spatial pattern analyses (e.g., the "dense in the east and sparse in the west" distribution) and mechanism interpretations (e.g., the evaluation of the effects of factors like river networks and topography). To address this, we have elaborated on the impacts of cholera data limitations on the conclusions regarding the spatiotemporal distribution pattern and its formation mechanisms in the "Research Limitations" subsection of the "Discussion" section (P 34-36). The specific revisions are detailed as follows:

***Research Limitations***

*(1) Cholera Data. The cholera epidemic data employed in this study were derived from systematic collection of historical documents. However, their quality was inevitably constrained by the varying levels of development of surveillance systems across different periods and regions—a common challenge faced in all historical disease research. First, diachronic changes in surveillance sensitivity might affect trend interpretation. In the early period (1949-1979), when the surveillance system was in its initial stage of establishment, potential case underreporting suggested that the "low-incidence periods" or "disease-free periods" documented in this study might, to a certain extent, reflect the limited surveillance capacity of that era rather than the absolute eradication of the disease. In contrast, the improvement of the surveillance network and the standardization of reporting systems since the 1980s and 1990s might have led to a "statistical increase" in recorded case numbers, which was superimposed on the actual epidemic fluctuations. Second, the uneven spatial coverage of surveillance might have amplified the observed east-west disparities. In the early stages, medical resources, surveillance awareness, and reporting systems in Wuhan (the provincial capital) and counties in the eastern plains were superior to those in the remote mountainous areas of western Hubei Province. Therefore, the sparse or even zero recorded cases in western Hubei Province were likely the combined result of "genuine low disease risk" and "insufficient sensitivity of the surveillance system". This could have led our model to overestimate the protective effects of natural geographical factors (e.g., high altitude, low river network density) and to reinforce the "ldense in the east and sparse in the west" spatial pattern. While reflecting real disease risks, the observed pattern might also have, to some degree, exaggerated the actual disparities between eastern and western regions. Thus, simply classifying western Hubei Province as a "low-risk area" might lack precision; a more rigorous characterization was that it was a "low-reporting area confirmed based on existing data", and its true epidemic situation awaits further investigation. Despite these limitations, we had strived to construct the most complete historical epidemic sequence through systematic cross-validation of multi-source local chronicles and yearbooks. Future research can further refine the reconstruction of historical epidemic dynamics by mining finer-grained historical archives.*

2. Regarding the 25-year cholera-free period (1955-1979), we indeed oversimplified its attribution to a single factor of "strict preventive vaccination measures", while failing to fully discuss the complex socio-political context, changes in the medical system, and the reliability of data reporting during this period. Your comments have prompted us to conduct a more comprehensive and critical re-examination of this historical phenomenon. Based on this, we have elaborated on the causes of the formation of the 25-year cholera-free period (1955-1979) and the recrudescence of cholera epidemics after 1980 in the subsection "Research Extension and Comparison" of the "Discussion" section (P 28-29). The specific revisions are detailed as follows:

***Discussion***

***Research Extension and Comparison***

*Temporal Variations in Cholera Epidemics in Hubei Province. First, the 25-year cholera silent period (1955-1979) identified in this study constitutes one of the salient features of its spatiotemporal distribution pattern. This phenomenon was a phased outcome of the combined effects of robust administrative interventions and a relatively closed social system under specific historical conditions [52]. In the early years of the People's Republic of China, the implementation of strategies such as preventive vaccination, transportation quarantine, and rapid blockade of epidemic foci, coupled with the household registration system and the prevailing international environment at that time, restricted population mobility. This significantly reduced the risk of pathogen introduction and dissemination, thereby sustaining the "zero-reporting" status throughout this period. However, the resurgence of cholera in Hubei Province since the 1980s was primarily driven by a shift in the underlying epidemiological mechanisms, which can be attributed to four key factors. First, the exponential increase in population mobility brought about by the reform and opening-up policy created favorable conditions for the introduction of Vibrio cholerae El Tor biotype, a novel pathogenic strain. Second, the enhanced environmental survivability and higher infectivity of this strain enabled it to easily breach the prior static defense system based on isolation control and vaccination [53]. Third, in the 1980s, drinking water safety issues remained unresolved in many regions, and a considerable number of cholera cases were caused by water source contamination. Fourth, some areas exhibited strategic flaws in epidemic prevention and control practices, including overly extensive blockade of epidemic sites, irrational coverage of chemoprophylaxis, and a prominent tendency of "prioritizing treatment over prevention". Moreover, the emphasis on economic development at the expense of social benefits led to delays in epidemic reporting and prevention, triggering the spread of cholera outbreaks. Thus, the cholera-free period of 1955-1979 represented a fragile epidemic prevention equilibrium achieved through administrative means within a closed system. In contrast, the epidemic trends since the 1980s have revealed that, against an open socioeconomic backdrop, the mechanism of cholera emergence has been restructured into a complex interplay of population mobility amid globalization, the evolutionary adaptation of pathogens themselves, and regional local environmental factors. At the same time, considering the evolution of China's national cholera control strategy, its approach had historically relied on robust public health measures, including the "three controls" (water, sewage, and food management), a sensitive surveillance system, and timely outbreak containment [42]. Although cholera vaccines were used as an adjunct in certain regions during the mid-20th century, they never became a cornerstone of the national prevention and control framework. This further suggested that the fluctuations in incidence observed in Hubei—such as the resurgence in the 1980s—were more attributable to shifts in environmental and socioeconomic drivers, as well as potential adjustments in the intensity of core sanitation measures.*

3. We have added a new subsection entitled "Data Quality and Limitations" (P 10) to the "Data source" section, in which we discuss the temporal variations in cholera surveillance capacity across different time periods in chronological order. The specific revisions are detailed as follows:

*（3）Data Quality and Limitations. Although the cholera epidemic data in this study have undergone multi-source verification, they still have certain limitations due to the constraints of technical conditions and objective environments in specific historical periods. The core influencing factors of data quality stem from the developmental differences in cholera surveillance systems across different eras, with specific manifestations as follows: Before the 1970s, the surveillance system was initially established and relied on manual case reporting. The data underreporting rate was high, and only the rough scope of the epidemic could be grasped, resulting in prominent limitations. During the 1980s—1990s, the surveillance network was gradually taking shape, and the sentinel system for intestinal clinics was fully popularized, which significantly improved the standardization and completeness of surveillance data. However, problems such as insufficient accuracy of strain typing still existed. Since the 21st century, the introduction of molecular biology technologies such as PCR and whole-genome sequencing has achieved a leap-forward improvement in the accuracy of surveillance data, providing core support for strain tracing and transmission chain analysis. With the real-time uploading and integration of data, the underreporting rate has dropped to below 1%. Nevertheless, insufficient grassroots testing capacity in remote areas remains a shortcoming.In general, China’s cholera surveillance system has shown a staged evolution characteristic of "from passive response to active intelligent early warning", and the data quality has undergone significant changes with socio-economic development and technological innovation[42].*

**Comment 1-2.**

Structural Equation Model: Conceptual and Technical Issues

The SEM is ambitious and represents the paper's main analytical contribution, but several concerns need addressing:

**1.Variable Selection and Conceptual Framework:**

•Why use "annual mean summer temperature" as a proxy for infection source rather than actual water temperature or seasonal Vibrio prevalence data? The conceptual linkage feels indirect. You justify this by stating summer is the high-incidence season, but then your seasonal analysis shows August-September-October peaks, which includes early autumn. This inconsistency needs explanation.

•The exclusion of critical variables is acknowledged in the Discussion but needs upfront acknowledgment as a major limitation:

No vaccination coverage data (yet you claim vaccination explains the 1955-1979 gap)

No water pH data (acknowledged as affecting Vibrio survival)

No sanitation infrastructure data

Road network density exists for only 6 time points (1962, 1980, 1990, 2000, 2010, 2020) with interpolated values for 66 other years. This temporal mismatch for a 72-year analysis is problematic and needs explicit justification in the Methods section.

**2.Interpretation Issues:**

•River network density shows a total effect of only 0.013 (Table 3)—essentially zero compared to population density (0.837) and summer temperature (0.807). Yet you state it "contributed to cholera epidemics levels." This coefficient is so weak it's arguably negligible. You should temper claims about river networks being a "driver," perhaps reframing them as a necessary background condition rather than an active direct factor in your model.

•Night Lights shows an extraordinarily high coefficient (1.080)—higher even than population density. While you link this to urbanization, such an extreme standardized coefficient can indicate model specification issues or unaccounted multicollinearity despite acceptable VIF values (7.527, though notably high). Night lights likely serves as a composite proxy overlapping substantially with population density (VIF=5.643) and GDP. The indirect effect is negative (-0.059), suggesting suppression effects. This needs careful interpretation—you cannot simply state it "significantly increases cholera" without acknowledging these statistical complexities.

•The positive effects of GDP and nighttime lights on cholera seem counterintuitive (wealthier areas should have better sanitation). Your explanation about "population mobility" and "wastewater discharge" feels post-hoc. An alternative explanation—that these variables reflect better disease surveillance and reporting in developed urban areas rather than true elevated risk—deserves serious consideration. Wuhan shows high cholera incidence in your maps and also has the highest GDP and night lights. Is this real elevated risk or better case detection?

**3.Technical Considerations:**

•You're essentially analyzing panel data (103 county-level observations across 72 years), but your SEM appears to treat this as cross-sectional data with time-averaged values. Have you considered:

Time-lag effects (e.g., does flood in year t affect cholera in year t+1)?

Non-stationarity in the time series?

Temporal autocorrelation within counties?

•The VIF test (Table 1) shows acceptable values, but you should clarify exactly what data structure was tested—are these 103 spatial observations with time-averaged variables, or something else?

•Table 3 is critical but difficult to interpret. The distinction between "overall," "direct," and "indirect" effects needs clearer explanation in the Methods. Readers unfamiliar with SEM path analysis will struggle to understand what these values represent.

4.Recommendation:

1.Add a supplementary table with complete variable descriptions: name, definition, unit, temporal coverage, source, and role in model

2.Revise interpretations to be more cautious, particularly for river networks (weak effect) and night lights (potential confounding)

3.Explicitly address the cross-sectional vs. time-series nature of your SEM

4.Consider alternative explanations for the GDP/night lights paradox

**Response 1-2.**

We sincerely appreciate your valuable feedback on the article "Structural Equation Modeling: Concepts and Technical Issues." Below, we address each of your comments and suggestions with specific revision approaches and responses:

1. Regarding variable selection and conceptual frameworks. First, your observation that using "annual mean summer temperature" as a proxy variable for the infection source is somewhat indirect, and that it does not fully align with the peak months of the epidemic, is crucial for refining our research. We have given this careful consideration and offer the following explanation and clarification: Firstly, the most ideal variables for infection sources would indeed be the concentration of Vibrio cholerae in water bodies or actual water temperatures. However, in our historical study covering 72 years (1949-2020) across 103 county-level units in Hubei Province, obtaining such long-term, large-scale, and high-precision monitoring data for waterborne microorganisms or water temperatures is impractical. Constrained by this data limitation, we had to identify a reliable, continuous, and accessible proxy variable. Since Vibrio cholerae reproduction and survival are highly temperature-dependent—with optimal growth occurring between 16-42°C—summer air temperature serves as the key determinant of surface water temperature. Furthermore, rigorously processed 1km-resolution raster data for "annual mean summer temperature" covering the entire study period and area are available from the National Qinghai-Tibet Plateau Science Data Center. This ensures consistency in our model's spatio-temporal analysis. Thus, selecting "annual mean summer temperature" represents a trade-off between theoretical support and data availability, aiming to capture the macro-level driving effect of temperature on Vibrio cholerae reproductive fitness. Second, while you observed epidemic peaks occurring from August to October, our model utilized "annual mean summer temperature" data from June to August primarily because Vibrio cholerae reproduction requires time. The elevated temperatures from June to August create optimal conditions for rapid bacterial proliferation in water bodies, leading to peak bacterial loads in the environment by late summer. However, a time lag exists between human exposure to contaminated water or food and the onset of symptoms, subsequent medical visits, and official case recording. Our model aims to capture these upstream environmental conditions driving outbreak occurrence. Based on this, we have made the following modification in the "Data Sources" section under "(2) Influencing Factor Data" (P 6):

***Data source***

*……*

*The reproduction and survival of Vibrio cholerae are highly dependent on environmental temperature, with its optimal growth temperature range being 16-42°C [25]. Ideally, water temperature is the most direct environmental factor driving the reproduction of Vibrio cholerae. However, given the long time span (72 years) and wide spatial scope (103 county-level units) of this study, it was not feasible to obtain consistent and continuous historical data on water temperature or aquatic Vibrio concentration. Summer air temperature is a key driver of surface water temperature, and there is a significant positive correlation between the two[26]. Meanwhile, cholera epidemics exhibit a distinct summer-autumn peak [27]. This peak shows a typical "time-lag effect" relative to summer high temperatures: high temperatures in summer promote the reproduction of Vibrio, leading to a peak in environmental bacterial load, which is then transmitted through water or food media, ultimately resulting in a case peak in late summer and early autumn (August-October). Therefore, this study selected the annual mean summer temperature as an important influencing factor of cholera infection sources.*

Third, the issue of systematic absence of relevant variables you pointed out is indeed a limitation that needs to be clearly clarified in this quantitative study. In accordance with your comments, we have added a subsection titled Data Quality and Limitations (P 10-11) to the "Data Source" section, where we discuss the limitations regarding the influencing factors of cholera. The specific revisions are as follows:

***Data source***

*……*

*(3) Data Quality and Limitations ……*

*The selection of influencing factors in this study was guided primarily by the theoretical framework, while also taking into account the principle of data availability. Constrained by the consistency and continuity of historical data series at the county scale, there were significant discrepancies in the completeness of datasets for various influencing factors: Among them, temperature, precipitation, and economic data could be continuously obtained at the county scale throughout the entire study period, with high data completeness; In contrast, only cross-sectional data at breakpoint of each era were available for population density and road network density datasets, making it impossible to form continuous time series. Data on drought and flood disaster sequences were missing after 2000; sunshine duration data were absent for the period 1949-1959; and nighttime light data were not available from 1949 to 1984; given the strong stability of topography and river-lake distribution, elevation and river network data from 2020 were used as substitutes. In addition, some factors that have been proven to affect cholera epidemics could not be quantitatively incorporated into the model due to data limitations. For example, vaccination coverage, water supply and sanitation facility conditions, etc., could not be directly included in statistical analyses due to the lack of standardized quantitative data at the county scale.*

2. Regarding the interpretation of results. First, you pointed out that the total effect of river network density is only 0.013, with a weak coefficient. We should downplay the expression that "river networks are driving factors" and can redefine them as "necessary background conditions" rather than "active direct influencing factors" in the model. This is indeed the case. It would be imprecise to continue referring to a variable with a low standardized coefficient as a "driving factor", which would undermine the credibility of the conclusions. We have revised the subsection Formation Mechanisms of Endogenous Variables in Cholera Epidemics (P 24) in the Results section and the Author summary section (P 2-3), reclassifying "river network density" from the role of a "driving factor" to that of a "fundamental background condition". The specific revisions are as follows:

***Formation Mechanisms of Endogenous Variables in Cholera Epidemics***

*……*

*(3) River network density, as a geographical background condition for epidemic transmission, contributed to the level of cholera epidemics (0.013). As a waterborne disease, cholera is transmitted through contaminated well water and river water [12]. Therefore, in areas with denser river networks, there are greater opportunities for Vibrio cholerae to come into contact with the population, making water-mediated transmission of cholera more convenient. Compared with the other two endogenous variables (population density and annual mean summer temperature), the effect value of river network density was relatively low. This data characteristic clearly revealed its attribute of being a "background condition rather than a primary driving factor". However, a low effect value did not equate to a secondary role; on the contrary, river network density served as the "fundamental premise" for the transmission of cholera as a waterborne infectious disease—it provided an indispensable geographical carrier for the survival, proliferation, and spread of Vibrio cholerae, forming a potential "hotbed" for the occurrence of cholera epidemics. The results of spatial distribution (Figures 8 and 9) clearly showed that cholera epidemics were highly concentrated in the Jianghan Plain with dense river networks and counties/cities along the Yangtze River and Han River, forming a significant spatial coupling relationship of "high river network density—high epidemic aggregation". In summary, it could be confirmed that a dense river network was the geographical basis for the occurrence of cholera epidemics; without this condition, the core pathway of water-mediated cholera transmission could not be established. However, the mere premise of dense river networks, in the absence of sufficient population density (to provide pathogen hosts) and suitable temperatures (to promote pathogen reproduction), could not trigger large-scale cholera epidemics either.*

***Abstract***

*……*

*Results: Temporally, high incidence occurred in the 1980s and 1990s, with summer and autumn being the dominant seasons. Three fluctuation cycles were identified: 29 years, 19 years, and 8 years. Spatially, 71 counties and districts were cumulatively affected, with Wuhan and Xianning serving as the primary hotspots. The epidemics was distributed along rivers and lakes, and its center of gravity shifted southward and westward over time. Mechanistically, cholera epidemics were the result of the combined effects of natural, disaster, and human factors. Population density and summer temperatures were the direct factors driving the spread of cholera epidemics, while river networks formed the basic environmental background that facilitated transmission. Natural environmental factors such as drought and flood disasters, altitude, and precipitation, as well as human environmental factors including road networks and economic conditions, could not only directly drive the spread of epidemics but also regulate epidemics development through indirect pathways. The coupling effects of various factors across different spatiotemporal scales jointly shaped the unique spatiotemporal distribution and evolutionary characteristics of cholera epidemics.*

***Author summary***

*Cholera remains a major threat to public health, especially in regions with dense water networks. As a "province of a thousand lakes" in China, Hubei* Province *has a long history of cholera epidemics, but its long-term spatiotemporal patterns and driving forces were not fully understood. We studied cholera cases in Hubei Province from 1949 to 2020, combining historical records and environmental data with analytical tools like spatial analysis and statistical modeling. We found cholera was most severe in the 1980s-1990s, peaked in summer and autumn, and had three cycles (29, 19, 8 years). Spatially, it clustered in eastern Hubei Province along rivers/lakes, with its center shifting southwest over time. Population density and summer temperature were the primary direct drivers of outbreaks, while river networks constituted a fundamental environmental backdrop that facilitated transmission. Floods, topography, and socioeconomic factors played additional indirect roles. Our findings filled gaps in Hubei’s cholera research and provided targeted containment and prevention clues for water-rich regions globally, thereby helping to balance environmental protection and disease control.*

Second, the two points you raised—the statistical complexity of the nighttime light coefficient and the interpretive dilemma of the positive effects of GDP/nighttime lights—were indeed weak points in the argumentation of our initial draft. Your comments have prompted us to conduct a more prudent rethinking and a more comprehensive interpretation of the model results, and we have supplemented and revised the discussion section to strive for a more rigorous and balanced argumentation. The specific revisions are as follows (P 30-31) :

***Discussion***

***Research Extension and Comparison***

*……*

*Regarding the influencing factors of cholera epidemics. The results of the structural equation model (SEM) showed that nighttime light exhibited a significant positive total effect on cholera epidemic levels (1.080), yet this finding required cautious interpretation. Nighttime light is a typical high-order composite variable that is strongly correlated with both population density and GDP. It may simultaneously capture multiple dimensions of information, such as population agglomeration in urbanized areas, intensity of economic activities, density of infrastructure, and even the capacity for surveillance and reporting. The coexistence of its positive direct effect (1.139) and negative indirect effect (-0.059) suggested that there may be unfully identified collinear structures within the model, or inhibitory effects generated through other pathways (e.g., improved medical response capabilities). Therefore, nighttime light should not be simply interpreted as an independent "risk driver"; instead, it ought to be regarded as a comprehensive indicator of "risk-surveillance co-occurrence" that marked regional development levels. Furthermore, the study found that GDP and nighttime light exerted positive impacts on cholera epidemic levels, which contradicts the common understanding that "economic development improved sanitation facilities and reduced the risk of waterborne infectious diseases". Two potential reasons for this discrepancy were hypothesized: First, real risk amplification effect. Economically developed and highly urbanized regions such as Wuhan, the large population size, frequent cross-regional mobility, dense transportation networks, and overloaded sewage treatment systems collectively create a favorable environment for cholera transmission. Historically, multiple cluster outbreaks had been concentrated in such areas, indicating that socio-economic activities themselves might exert an "amplification effect" on epidemic spread. Second, surveillance and reporting bias. Economically developed regions were equipped with more robust infectious disease surveillance systems, advanced diagnostic capabilities, and stringent reporting protocols, resulting in far higher case detection and reporting rates than underdeveloped regions. The observed "high epidemic levels" might not stem entirely from differences in actual incidence; instead, they could be systematic errors caused by disparities in public health surveillance capacity—a problem that was difficult to avoid in retrospective studies utilizing passively reported data.*

3. First of all, thank you for your suggestions. You proposed that the differences between "total effect", "direct effect" and "indirect effect" should be explained more clearly in the methodology section to help readers unfamiliar with structural equation model (SEM) path analysis understand the meanings of these values. We have added explanations of "total effect", "direct effect" and "indirect effect" at the end of the "Structural Equation Model" subsection in the "Research Methodology" section (P 14), and the specific revisions are as follows:

***Structural Equation Model.……***

*Among these, the effect value can be further decomposed into direct and indirect effects. Table 4 presents precisely this decomposition, interpreted as follows: The direct effect refers to the strength of a variable's direct influence on another variable. It manifests as the standardized path coefficient between the two variables after controlling for all other variables in the model, reflecting the "net effect" of the independent variable on the dependent variable. For example, after controlling for variables such as the average annual summer temperature and river network density, the path coefficient from "population density" to "level of cholera prevalence" represents the direct effect of population density on cholera outbreaks. Indirect effects refer to the cumulative impact of a variable on the dependent variable through one or more mediating variables, calculated as the sum of the products of the standardized coefficients across all mediating paths. For instance, "road network density" may influence cholera epidemic levels by first increasing "population density" (path: road network density → population density), which then affects cholera epidemic levels (path: population density → cholera epidemic levels). The combined effect generated by this transmission path constitutes the indirect effect of road network density. The total effect is the sum of the direct effect and all potential indirect effects, representing the overall strength of the independent variable's influence on the dependent variable through all defined pathways in the model. This satisfies the quantitative relationship: Total Effect = Direct Effect + Indirect Effect.*

Second, following your suggestion, we have added a new table titled "Definitions and Processing Instructions for All Variables in the Model" to the "Data Sourcesl" section. This table includes the definitions, units, time ranges, and roles of each factor within the structural equation model, facilitating a clearer understanding of how each variable functions within the model. The specific changes required are as follows（P 7-10）:

***Data source***

***……***

*(2) Influencing factor data.……The structural equation model in this study aims to identify the steady-state drivers shaping the long-term spatial patterns of cholera. To this end, we aggregated the data along the temporal dimension. Specifically: for each variable across all counties, we calculated a representative value spanning the entire study period from 1949 to 2020. For cholera case counts, we used cumulative total cases; for continuous variables like temperature and precipitation, we employed multi-year averages; for flood and drought disasters, we utilized cumulative occurrence years. Thus, the final data structure input to the model is a cross-sectional dataset comprising 103 observations, each representing a county's aggregated characteristics over 72 years. All variables involved in this study—including their definitions, units, time ranges, processing methods, and roles within the structural equation model—are summarized in Table 1.*

*Table 1 Definitions and Processing Instructions for All Variables in the Model*

| ***Variable Name*** | ***Variable Definition and Description*** | ***Unit*** | ***Time Coverage and Processing Method*** | ***Role in the Model*** |
| --- | --- | --- | --- | --- |
| ***Cholera Epidemic*** | *The cumulative total of confirmed cholera cases reported by each county between 1949 and 2020 reflects the historical cumulative intensity of cholera epidemics in the region and serves as a core observational indicator for research.* | *cases* | *Complete study period from 1949 to 2020, utilizing cumulative confirmed case counts from county health statistics reports and disease surveillance systems, with duplicate reports excluded.* | *Dependent variable, representing the outcome variable of a cholera epidemic* |
| ***Population Density*** | *The density of regional population distribution reflects the concentration of susceptible individuals and serves as a key demographic indicator influencing the transmission efficiency of infectious diseases.* | *Persons/km²* | *Based on the data from 7 census years (1953, 1964, 1982, 1990, 2000, 2010, and 2020), the county-scale data were supplemented by the spatial interpolation method, and the average value during the study period was calculated.* | *Endogenous variable, serving as a proxy variable for the size of susceptible population* |
| ***Annual mean summer temperature*** | *The average temperature from June to August (summer) each year reflects the cumulative summer heat level of a region and affects the survival and reproduction efficiency of Vibrio cholerae.* | *°C* | *For the complete study period from 1949 to 2020, county-scale data were extracted through spatial overlay analysis based on monthly temperature raster data, and the annual average values during the study period were calculated.* | *Endogenous variable, serving as a proxy variable for the living environment of Vibrio cholerae (infectious source)* |
| ***River Network Density*** | *The ratio of the total length of rivers to the regional area within a county reflects the degree of water system development and the spatial characteristics of water body coverage, and serves as a key environmental carrier indicator for cholera water-borne transmission.* | *km・km⁻²* | *As static geographic data, river elements within county boundaries were extracted based on the 1:100,000 water system vector data from Open Street Map (OSM), and the total river length per unit area was calculated.* | *Endogenous variable, serving as a proxy variable for the water-borne transmission route of cholera* |
| ***Annual Mean GDP*** | *The average annual gross domestic product (GDP) within a county, which reflects the regional economic development level, the capacity for public health resource allocation, and the potential for medical prevention and control investment.* | *ten thousand yuan* | *Actual measurement data from 1949 to 2020 (sourced from statistical yearbooks), with annual averages calculated for the study period.* | *Exogenous variable, serving as a core indicator representing the level of social and economic development* |
| ***Night Lights*** | *The annual average nighttime light radiation intensity within a county reflects the regional urbanization level, intensity of human activities, and the level of infrastructure improvement* | *nW/(cm²·sr)* | *From 1985 to 2020, based on the DMSP-OLS (Defense Meteorological Satellite Program-Operational Linescan System) nighttime light dataset, the county-level average values were calculated after conducting data consistency correction.* | *Exogenous variable, serving as a proxy variable for urbanization and human activity intensity* |
| ***Road Density*** | *The ratio of the total length of highways/roads within a county to the regional area reflects traffic accessibility, the convenience of personnel mobility, and the efficiency of public health emergency response.* | *km・km⁻²* | *Covering 6 time nodes (1962, 1980, 1990, 2000, 2010, and 2020), county-level highway networks were extracted based on transportation vector map data, and the average value of total highway length per unit area was calculated* | *Exogenous variable, an indicator representing traffic connectivity and emergency response capacity* |
| ***Mean Elevation*** | *The average value of topographic altitude within a county reflects the characteristics of regional terrain relief, affects climatic conditions and human settlement patterns, and indirectly acts on cholera transmission* | *m* | *As static geographic data, it was obtained through county boundary clipping and mean value statistics based on the 30m-resolution SRTM DEM* | *Exogenous variable, a basic indicator representing natural geographical characteristics* |
| ***Annual Mean Precipitation*** | *The annual average precipitation within a county reflects the characteristics of regional aridity and humidity, affects the stability of the aquatic environment and the transmission vector conditions of Vibrio cholerae.* | *mm* | *For the complete study period from 1949 to 2020, county-scale data were extracted via spatial overlay analysis based on the 0.5°×0.5° monthly precipitation raster data, and the annual average values over the study period were calculated.* | *Exogenous variable, a key indicator representing natural climatic characteristics* |
| ***Annual Mean Sunshine*** | *The annual average value of sunshine duration in a county is able to reflect the degree of abundance of solar radiation resources in the region* | *hour* | *From 1960 to 2020, based on the monthly sunshine raster data generated from meteorological station observation data and spatial interpolation, the annual average values at the county scale over the study period were calculated* | *Exogenous variable, an indicator representing climatic conditions* |
| ***Years of Flooding*** | *The cumulative frequency of flood disasters within counties from 1949 to 2000 reflects the intensity of water inundation events caused by heavy rainfall, snowmelt and other factors, and is prone to triggering waterborne transmission of cholera* | *Frequency* | *From 1949 to 2000, flood disaster events with clear time records within counties were counted based on historical disaster records* | *Exogenous variable, an indicator representing disaster disturbance factors* |
| ***Years of Drought*** | *The cumulative frequency of drought disasters in counties from 1949 to 2000 reflects the intensity of water shortage events caused by long-term insufficient precipitation, and affects sanitary conditions and disease prevention and control* | *Frequency* | *From 1949 to 2000, drought disaster events with clear time records within counties were counted based on historical disaster records* | *Exogenous variable, an indicator representing disaster disturbance factors* |

*Note: This model adopts a time aggregation strategy, where all variables are processed into a representative value for each county-level unit over the study period (1949-2020), forming a cross-sectional data structure for structural equation model (SEM) analysis*

Third, regarding the technical considerations you raised. To construct a stable, interpretable causal path model for investigating the long-term, macro-level mechanisms of cholera formation, we aggregated the 72-year panel data temporally , forming cross-sectional data for 103 county-level units. The primary reason is that our research focuses on revealing the relatively stable geographic and socioeconomic background factors (such as topography, river network density, average climate conditions, and economic development levels) shaping the long-term spatial pattern of cholera in Hubei Province, rather than short-term fluctuations. Second, as noted in the "Data Quality and Limitations" section, complete and continuous 72-year time series were unavailable for certain critical variables (e.g., population density, road network density, disaster data), with only discontinuous annual data accessible. Using time-averaged values was the most feasible approach to ensure all variables could be incorporated into the same model. Thus, simplifying complex spatiotemporal panel data into spatial cross-sectional data avoided highly intricate econometric issues like time lags, non-stationarity, and spatiotemporal autocorrelation, resulting in a more robust model and easier-to-interpret outcomes. Furthermore, in the subsection "Construction and Validation of a Cholera Epidemics Impact Mechanism Model" within the main text, the VIF tests presented here are also based on cross-sectional data from 103 county-level units, where all variables have been aggregated over time.

Regarding time-lag effects, non-stationarity, and autocorrelation. Within the current time-aggregated cross-sectional SEM framework, we are indeed unable to directly model these temporal dynamic characteristics. We can add a paragraph in the "Discussion and Conclusions" section under "Research Limitations" stating: We recognize the importance of these temporal dynamic effects. Our Figure 11, "Comparison of time series between cholera and influencing factors in Hubei Province from 1949 to 2020", visually illustrates the standardized time series of key variables alongside cholera case counts. The figure reveals, for instance, a rough "trailing" relationship between flood disaster years and subsequent cholera peaks, alongside inherent fluctuations across all series. However, rigorous quantitative validation of these dynamic relationships requires more comprehensive high-frequency panel data and sophisticated spatiotemporal econometric models—beyond the scope of this paper but representing a crucial avenue for future research.The specific changes required are as follows（P 35-36）:

***Discussion***

*……*

***Research Limitations***

*……*

*(3) Structural Equation Modeling. ……Additionally, the model inadequately accounts for temporal dynamics. The structural equation model employed a time aggregation strategy to identify steady-state drivers shaping long-term spatial patterns, thereby failing to capture dynamic characteristics during cholera epidemics. First, the model did not incorporate time-lag effects, such as the potential months- or even years-long delay between major floods and subsequent cholera outbreaks. Second, the current cross-sectional model did not address potential non-stationarity in the time series of individual variables and cholera itself, nor did it account for autocorrelation within county-level units over time. Although presented as exploratory analysis, Figure 11 displays standardized time series of key variables alongside cholera cases, visualizing their covariate trends and potential lagged relationships. However, rigorous validation of time lags, analysis of non-stationary series, and construction of spatiotemporal panel models require more complex econometric methods applied to more complete, higher-frequency panel data—representing a key direction for future research.*

**Comment 1-3.**

Statistical Rigor and Interpretation of Temporal Patterns

Several statistical findings need stronger justification or clarification:

**1.M-K Test Inconsistency**: The text states "a significant downward trend was observed from 1955 to 1994" based on the UF curve (Figure 4). However, this period includes your high-incidence phase (1980-1999) with 88.4% of all cases. How can there be a downward trend during a period of epidemic resurgence? Looking at Figure 3, cases clearly increase from 1980-1988. Please clarify what the M-K statistic is actually showing here—is this an error in interpretation, or are you detecting a trend in variance rather than mean?

**2.Wavelet Analysis - The 55-Year Cycle**: You identify four fluctuation cycles: 55, 29, 19, and 8 years, with

the 55-year cycle described as the "first main cycle." However:

• Your study span is 72 years with only 31 years of actual epidemic data

• A 55-year cycle can only complete about 1.3 cycles within your timeframe

• You have essentially one peak and one trough to define this "cycle"

• Figure 6 shows the 55-year peak is only marginally higher than the 29-year peak

This is statistically precarious. You cannot reliably detect a cycle that nearly spans your entire observation period. The wavelet analysis is better suited for identifying the 29, 19, and 8-year cycles. I recommend either:

1. Stating explicitly that the 55-year cycle is "tentative" or "preliminary" given data constraints

2. Removing it from the main findings and discussing it as a limitation

3. Providing statistical confidence intervals for the wavelet variance peaks

**Contextualization of Cycles:** The four cycles need better interpretation beyond mathematical description.

Are they linked to:

• Climate oscillations (e.g., ENSO, PDO)?

• Socio-political transitions (policy changes, healthcare reforms)?

• Pathogen evolution (El Tor introduction in 1961, O139 in 1993)?

• Generational immunity cycles?

The Discussion briefly mentions El Tor and O139 but doesn't connect these to your cyclical findings. The 29-year cycle roughly corresponds to the time between the 1960s El Tor introduction and the 1990s peak—is this coincidental or mechanistically related?

**3.Phase Classification**: You divide the timeline into four phases (1949-1954, 1955-1979, 1980-1999, 2000-2020) but state "No abrupt change points were detected between the UF and UB curves." If there are no statistical change points, what justifies these exact boundaries? The phase division appears to be based on visual inspection of Figure 3 rather than statistical criteria. Please clarify your methodology for defining these periods

**Response 1-3.**

Thank you for your valuable comments on the "statistical rigor and interpretation of temporal patterns" of the article. Below is the specific revision and response plan for each of your comments and suggestions:

1. We would like to express our sincere gratitude for this highly professional and critical question you raised. You astutely pointed out the inaccuracy in our original description of the Mann-Kendall (M-K) test results—referring to the entire period from 1955 to 1994 as showing a "significant downward trend", which seems contradictory to the fact that this period included an epidemic upsurge phase (1980-1988). This prompted us to re-examine and more precisely elaborate on the implication of the M-K test results for this specific data series. First, the sharp increase in the number of cholera cases from 1980 to 1988 is an objective fact, which is clearly illustrated in Figure 3. The generalized statement in our original manuscript that a "significant downward trend was observed from 1955 to 1994" is indeed prone to misunderstanding. The fundamental reason lies in the inherent characteristics of the M-K test and its sensitivity to the starting point and long-term state of the data series. Our time series starts from 1949 and includes a consecutive 25-year cholera-free period (1955-1979) with a constant value of 0 (no cases reported). The UF statistic of the M-K test begins to be calculated from 1955 (the start of the cholera-free period), and this prolonged "zero-value plateau" has a profound impact on the judgment of subsequent trends. Therefore, the "downward trend" indicated by the continuously negative UF curve during 1955-1994 needs to be interpreted in combination with the specific structure of the data series: it does not mean that the number of cases decreased monotonically throughout this period. On the contrary, it reflects that starting from the baseline state of "zero cases" in 1955, after experiencing an explosive growth and reaching a peak in the 1980s, the number of cases did not maintain such an extremely high level, but instead showed an overall tendency of "declining from the peak". The test captures the falling tendency of the endpoint value of the entire long period (starting from 1955 and ending in 1994) relative to the extreme value reached after the initial "zero-value plateau". The reference frame for "decline" is the implicit "high-level state". Due to the exceptionally prominent peak in 1988, the number of cases in subsequent years, although still relatively high, was significantly lower than the peak; thus, from the long-range perspective of the non-parametric test, a downward trend was identified. To correct this inaccurate expression and avoid misunderstanding, we have revised the discussion in the "Trends" subsection under the Temporal changes in the cholera epidemics in Hubei Province section of the Results (P 15-16). The specific revisions are as follows:

*A Mann-Kendall (M-K) test was performed on the time series of cholera cases in Hubei Province from 1949 to 2020 (Fig 4). The UF curve shows that after 1955 (the start of the cholera-free period), the statistic remained in the negative range for a long time and fell below the significance threshold of -1.96 multiple times between 1955 and 1994. Among these, the 25-year consecutive cholera-free period (1955—1979) at the beginning of the series profoundly influenced the trend judgment. A negative UF value indicates that, from the perspective of the long-term baseline state, after the number of cases rose sharply in the 1980s, its overall level did not continue to move toward higher extreme values but instead showed a statistical tendency to decline from the peak range. Specifically, the rate of decline was relatively fast during 1955-1978 (corresponding to the cholera-free period) and gradually slowed during 1979-1994 (which included the epidemic outbreak and high-level fluctuation period). After 1995, the UF curve fluctuated within the non-significant range until it exhibited a significant downward trend again from 2013 onward, which is consistent with the actual situation that the epidemic entered a "low-incidence and controllable phase" after 2000. No intersecting abrupt change points were observed between the UF and UB curves, indicating that the long-term overall trend of cholera incidence showed a downward tendency throughout the entire study period from 1949 to 2020.*

2. First, thank you very much for your expert advice regarding the statistical uncertainty of the 55-year cycle. You correctly pointed out that for a 72-year sequence, detecting a cycle approaching its length (55 years) is statistically unreliable. We have revised the paper accordingly: First, we removed the 55-year cycle as a primary finding. In the "Results" section, we no longer describe the 55-year cycle as the "first principal cycle" or primary cycle. The wavelet variance analysis now explicitly focuses on the three statistically reliable cycles: 29 years, 19 years, and 8 years. Second, we have updated the textual descriptions and conclusions. All relevant main text, conclusions, and abstracts have been revised to emphasize only these three cycles. Third, we have adjusted the figures. Figures 5, 6, and 7 have all been modified. The specific changes are as follows(P 16-19):

***Cyclical variations***

*The real part of the Morlet wavelet transform coefficients reflects the fluctuation characteristics of data within the wavelet domain across different time scales. The alternating positive and negative values of the real part correspond to the severity characteristics of the cholera sequence in the time domain. Plotting the isocontour map of the real part of the wavelet transform coefficients for the cholera epidemic sequence (Fig 5), the colors represent the sign and magnitude of the real part of the wavelet coefficients: red regions indicate positive real parts of the wavelet coefficients, corresponding to periods of elevated cholera case numbers; blue regions indicate negative real parts of the wavelet coefficients, corresponding to periods of reduced case numbers. It can be observed that the cholera epidemics in Hubei Province exhibited different periodic variations and alternating high-low incidence processes across different time scales, forming oscillation centers with alternating positive and negative values at various scales. Overall, the cholera epidemics in Hubei Province from 1949 to 2020 showed fluctuation cycles at three time scales: 25-35a, 16-22a, and 5-10a. Specifically: At the 25-35a time scale, there were 4 quasi-oscillation cycles with alternating high and low incidence, which were relatively stable and exhibited full-domain coverage throughout the entire study period. At the 16-22a time scale, there were 4 quasi-oscillation cycles with alternating high and low incidence, which mainly occurred between 1960 and 2005. At the 5-10a time scale, there were 4 quasi-oscillation cycles with alternating high and low incidence, which mainly occurred between 1980 and 1999.*

***Fig. 5 Real contour plot of wavelet transform coefficients of cholera caces in Hubei Province***

*Wavelet variance maps can reflect the distribution of fluctuation energy of cholera sequences over time scales, and can be used to determine the existence of multilevel main cycles of cholera sequences. Plotting the wavelet variance map of cholera sequences in Hubei Province (Fig 6), it could be seen that there were three obvious peaks in the cholera sequences in Hubei Province between 1949 and 2020, which corresponded to the time scales of 29, 19, and 8 years in turn. Among them, the largest peak corresponds to the 29a time scale, indicating that the cyclic oscillation around 29 years was the strongest and serves as the first main cycle of cholera variations in Hubei Province; the 8a and 19a time scales were the second and third main cycles, respectively. These three scales of cycle fluctuations controled the characteristics of cholera changes throughout the time domain.*

***Fig. 6*** ***Wavelet Variance Plot of Cholera Case Count Time Series in Hubei Province***

***Fig. 7 Process lines of the real part of wavelet transform coefficients of the number of cholera cases in Hubei Province under 29a time scale and 8a time scale cycles***

*According to the results of the wavelet variance test, plotting the process lines of the real part of the wavelet transform coefficients of the 29a scale time and 8a scale time main cycles of the cholera case count series in Hubei Province during 1949-2020 (Fig. 7), we can analyze the characteristics of the mean cycle and light-heavy change that existed during the process of the cholera change in Hubei Province. On the 29a time scale, the mean change cycle of the number of cholera counties was about 17a, and it had experienced about 4 cycles of light-heavy transition, with the periods of heavy epidemics: 1949-1954, 1964-1972, 1983-1992, 2003-2012, and the periods of light epidemics: 1955-1963, 1973-1982, 1993-2002, and 2013-2020. On the 8a time scale, the mean change cycle of the number of cholera counties is about 7a, and it has experienced about 10 distinct cycles of light-heavy transition, with the periods of heavy epidemics: 1949-1950, 1954-1956, 1972-1974, 1978-1979, 1983-1984, 1988-1989, 1993-1995, 1998-2000, 2004-2005, 2009-2013, and the periods of light epidemics: 1951-1953, 1957-1959, 1975-1977, 1980-1982, 1985-1987, 1990-1992, 1996-1997, 2001-2003, 2006-2008, 2014-2016.*

Second, we fully agree that providing a mechanistic interpretation of the identified periodic phenomena is the key to advancing the research from "description" to "understanding", and we are deeply grateful for this insightful suggestion. In accordance with your advice, we have attempted to conduct a correlation analysis between the three statistically significant cycles (29-year, 19-year, and 8-year) and macro processes such as climate oscillations, socioeconomic development, and pathogen evolution in the Research Extension and Comparison subsection (P 30) of the "Discussion" section, thereby endowing these mathematical cycles with potential biological and sociological significance. The specific revisions and newly added content are as follows:

*In addition, this study identified three significant cholera epidemic cycles of 29, 19, and 8 years, which may reflect the coupling of multi-scale driving processes. The 29a cycle aligned with the epidemiological cycle of "introduction-epidemic-suppression" for major Vibrio cholerae biotypes (e.g., the El Tor biotype in the 1960s) and concurrent socio-economic transformations (e.g., the Reform and Opening-up period), suggesting it was a composite biosocial cycle shaped jointly by pathogen, host, and social factors. The 19a cycle corresponded closely to the activity period of low-frequency climate modes such as the Pacific Decadal Oscillation, potentially modulating regional climatic and hydrological backgrounds and setting an environmental rhythm at the decadal scale for epidemics. The 8a cycle was associated with interannual climate variability such as the El Niño-Southern Oscillation and the triggering frequency of flood disasters, reflecting the role of extreme weather events as direct "triggers" for outbreaks. Together, these cycles constituted the comprehensive temporal characteristics of cholera epidemics under multiple mechanisms, including long-term pathogen evolution, medium-term climatic fluctuations, and short-term disaster triggers.*

3. Thank you very much for raising this important methodological question. You correctly point out that we divided the data into four phases based on visual observations of Figure 3, yet the Mann-Kendall test did not detect any statistically significant breakpoints. This is indeed a key point we need to clarify. First, the M-K test (used to detect trend breaks) and our phase divisions serve two distinct yet complementary analytical purposes. The four-phase classification (1949-1954: low incidence period; 1955-1979: disease-free period; 1980-1999: high incidence period; 2000-2020: controllable period) is primarily grounded in historical epidemiological characteristics and phased shifts in disease control practices. This constitutes an "interpretive" or "descriptive" periodization. The core rationale does not stem from inflection points identified by the M-K test, but rather from the following objective facts and professional judgments: 1949 marked a pivotal starting point for systemic social transformation, signifying the inception of a structured public health system. The 25-year period from 1955 to 1979, with no reported cases, constitutes a clear "epidemic-free period" defined by zero cases. The period from 1980 to 1999 encompassed intensified population mobility following reform and opening-up, the introduction and localization of the seventh cholera pandemic (El Tor type), and a distinct high-incidence phase accounting for 88.4% of total cases. After 2000, with the refinement of the national infectious disease direct reporting system and sustained increases in health investments, reported cholera cases declined to extremely low levels, entering a state of "sporadic and controllable" occurrence. This periodization aims to provide readers with a clear historical epidemiological narrative framework for understanding the macro-level trajectory of cholera's "emergence-disappearance-re-emergence-re-control" in Hubei. It represents a qualitative summary of historical processes. To clarify this distinction and prevent reader misunderstanding, explicit clarification has been added to the ‘Trends’ subsection (P 15) within the "Results, Temporal changes in the cholera epidemics in Hubei Province" section. The specific modification is as follows:

***Trends***

*From 1949 to 2020, over a span of 72 years, cholera was reported in Hubei Province in 31 of those years (Fig. 3), with a cumulative total of 3,608 cases. The years with the highest number of cholera cases were 1988 (1,351 cases), 1994 (396 cases), 1984 (347 cases), 1983 (294 cases), and 1998 (204 cases). To facilitate the description of the macroevolution of cholera in Hubei Province from a historical epidemiological perspective, we first divided the period from 1949 to 2020 into four descriptive phases based on the distribution characteristics of case numbers, important social and historical nodes, and transitions in disease prevention and control stages. 1949-1954 (Low Incidence Period): In the early years after the founding of the People's Republic of China, cholera cases in Hubei Province were sporadic, with a cumulative total of 345 reported cases, marking a relatively low-incidence phase. 1955-1979 (Cholera-Free Period): Hubei Province experienced no cholera cases for 25 consecutive years, representing a disease-free phase. 1980-1999 (High Incidence Period): This marked the first major cholera epidemic after 1949, with a cumulative total of 3,193 cases, accounting for 88.4% of all cholera cases in Hubei Province, making it a high-incidence phase. 2000-2020 (Controlled Incidence Period): Cholera epidemics were gradually brought under control, with the cumulative number of cases dropping to just 70, indicating a controlled-incidence phase.*

**Comment 1-4.**

Spatial Analysis and Presentation

**1.Missing Visualizations:**

• You mention local spatial autocorrelation analysis and state results about "High-High and LowLow clusters" in the text, but Figure 8b only shows overall Moran's I results. Please add maps showing the LISA (Local Indicators of Spatial Association) results with High-High, Low-Low, High-Low, and Low-High clusters clearly marked for different time periods.

**2.Center of Gravity Analysis:**

• The shift from northeast to southwest is interesting and well-presented in Figure 9f. However, you

don't adequately explain why this shift occurred. What changed in these regions? You mention that

southeastern Hubei (Xianning) became a hotspot, but was this due to:

o Population growth and urbanization?

o Changes in water management or sanitation?

o Economic development patterns?

o Proximity to new transportation routes?

**3.Seasonal Distribution Paradox:** You state cholera peaked in "August, September, and October" with August (31.56%), September (33.05%), and October (15.29%) being highest. October is technically autumn, not summer. Yet you use "annual mean summer temperature" (June-August) as your SEM variable. There's a temporal mismatch between when cholera actually peaks (Aug-Oct) and your temperature proxy (Jun-Aug). This needs explanation—is there a 1-month lag effect, or should you be using a different seasonal temperature variable?

**Response 1-4.**

Thank you for your valuable comments on the "spatial analysis and presentation" of the article. Below is the specific revision and response plan for each point:

1. Thank you for your attention to the visualization results. We would like to clarify that Figure 8b is itself a cluster map generated based on the Local Indicators of Spatial Association (LISA) analysis, which is used to display the specific spatial distribution of agglomeration areas such as high-high and low-low clusters. Misunderstanding may have arisen due to the insufficient description of the LISA results in the previous main text. We have added a detailed paragraph in the "Overall distribution characteristics" section (P 19-20) of the Results to systematically interpret the hotspots, coldspots and their spatial patterns revealed by the LISA map. The specific revisions are as follows:

***Overall distribution characteristics***

*Over the 72-year period from 1949 to 2020, among the 103 county-level administrative units in Hubei Province, a total of 71 counties/districts reported cholera epidemics, accounting for approximately 70% of the total. The cumulative number of cholera cases reached 3,475. The hardest-hit areas were mainly concentrated in Daye (344 cases) and Xian'an (326 cases) in the Xianning region, followed by Jianli (168 cases), Jiangling (143 cases), and Hanchuan (141 cases). Light or non-epidemic areas were primarily located in western Hubei Province, including Shiyan, Shennongjia, and Enshi (Fig. 8a). Spatial autocorrelation analysis revealed that the cholera epidemics in Hubei Province exhibited a significant clustered distribution pattern, with a Moran's I value of 0.35, a Z-score of 6.35 (p < 0.01). Local Indicators of Spatial Association (LISA) analysis (Fig. 8b) further accurately identified the spatial locations and types of local hotspots and coldspots. High-high (HH) agglomeration areas were mainly continuously distributed in the core urban districts of Wuhan, such as Wuchang, Hanyang, and Jiangxia, as well as in Xianning City in the southeast (including Xian'an, Chongyang, and Yangxin). In contrast, low-low (LL) agglomeration areas were concentrated in Shiyan City, Shennongjia Forestry District, and most parts of Enshi Tujia and Miao Autonomous Prefecture in western Hubei Province.*

2. Thank you very much for your valuable comments. You pointed out that although we identified the southwestward shift of the cholera epidemic focus, the explanation for its driving factors is insufficient, and this comment is very pertinent. We have supplemented the discussion on the causes of the migration of the epidemic focus in the "Discussion" section of the paper (P 30). The specific revisions are as follows:

*Regarding the spatial changes of cholera epidemics in Hubei Province. The shift in the epidemic's center of gravity was not merely a result of the simple accumulation of cases; instead, it reflected the transformation of core areas of population and socio-economic activities within the province. During 1949-1959, the center was located in the Xiaogan region, which corresponded to the relatively higher population density and agricultural activity levels in the eastern Jianghan Plain (e.g., Xiaogan and Huangpi). With the rise of Wuhan as a central city in China after the 1980s, its massive population size and frequent internal and external exchanges made it an indisputable hotspot for cholera outbreaks in the 1980s-1990s, leading to a southward shift of the center to urban Wuhan (e.g., Jiangxia and Honghu). From the late 1990s to the 2000s, although the number of cases in Wuhan decreased, counties along the Yangtze River, such as Jingzhou and Jiangling, emerged as new key epidemic areas due to their role as secondary population centers in the province and their traditional reliance on water bodies for rice cultivation. This collectively contributed to a further southwestward shift of the center toward the hinterland of the Jianghan Plain (e.g., Honghu). This trajectory essentially outlined how the dynamic changes in the center of socio-economic activities and population distribution in Hubei Province over the past half-century had shaped the spatial pattern of infectious disease risks.*

3. You observed that the epidemic peak occurs from August to October, while we selected the "annual mean summer temperature" from June to August as a key variable in our model. This is primarily based on the lag mechanism between Vibrio cholerae proliferation and disease transmission. The high temperatures from June to August provide optimal conditions for the rapid reproduction of Vibrio cholerae in water bodies, leading to a peak in environmental bacterial load by late summer. However, there is a typical epidemiological delay from human exposure to contaminated water or food, to symptom onset, seeking medical care, and finally being recorded as a case. Therefore, the high temperatures from June to August serve as the proximal environmental driver of the epidemic, while the case peak from August to October is its delayed manifestation. Our model aims to capture this critical environmental driving condition that precedes the outbreak. Relevant explanations have been added in the "(2) Influencing factor data" subsection of the "Data source" section, with specific modifications as follows (P 6):

*The reproduction and survival of Vibrio cholerae are highly dependent on environmental temperature, with its optimal growth temperature range being 16-42°C [25]. Ideally, water temperature is the most direct environmental factor driving the reproduction of Vibrio cholerae. However, given the long time span (72 years) and wide spatial scope (103 county-level units) of this study, it was not feasible to obtain consistent and continuous historical data on water temperature or aquatic Vibrio concentration. Summer air temperature is a key driver of surface water temperature, and there is a significant positive correlation between the two[26]. Meanwhile, cholera epidemics exhibit a distinct summer-autumn peak [27]. This peak shows a typical "time-lag effect" relative to summer high temperatures: high temperatures in summer promote the reproduction of Vibrio, leading to a peak in environmental bacterial load, which is then transmitted through water or food media, ultimately resulting in a case peak in late summer and early autumn (August-October). Therefore, this study selected the annual mean summer temperature as an important influencing factor of cholera infection sources.*

**Minor Comments**

**Comment 1-1.**

**Abstract and Author Summary**

• **Abstract - Future Tense:** Change "This study will help to reveal" to "This study reveals" or "Our findings fill" (past tense is appropriate for completed research)

• **Inconsistency between Abstract and Author Summary**: The Author Summary states floods and topography played "indirect roles," while the main Abstract simply lists them as factors. This distinction between direct/indirect effects is a key finding from your SEM and should be consistent across both summaries.

**Response 1-1.**

Thank you for your valuable comments on the "Abstract and Author Summary" section of the article. Below is the specific revision and response plan for each point:

1.Thank you for bringing the tense issue in the article abstract to our attention. Based on your suggestion, we have revised the abstract from "This study will help to reveal" to "This study helped to reveal".

2.Thank you very much for your meticulous comparison of the expressions in the two abstracts. The inconsistencies you pointed out are entirely correct, and they did affect the clear communication of the core findings. The mechanism of "direct and indirect effects" revealed by the structural equation model is one of the core contributions of this study, which should certainly be clearly and consistently presented in both abstracts. In accordance with your comments, we have unified and strengthened the descriptions of influencing factors in both the Abstract and the Author Summary. The specific modifications are as follows(P 2-3):

***Abstract***

*……*

*Results: Temporally, high incidence occurred in the 1980s and 1990s, with summer and fall being the dominant seasons. three fluctuation cycles were identified: 29 years, 19 years, and 8 years. Spatially, 71 counties and districts were cumulatively affected, with Wuhan and Xianning serving as the primary hotspots. The epidemic was distributed along rivers and lakes, and its center of gravity shifted southward and westward over time. Mechanistically, cholera epidemics were the result of the combined effects of natural, disaster, and human factors. Population density and summer temperatures were the direct factors driving the spread of cholera epidemics, while river networks formed the basic environmental background that facilitated transmission. Natural environmental factors such as drought and flood disasters, altitude, and precipitation, as well as human environmental factors including road networks and economic conditions, could not only directly drive the spread of epidemics but also regulate epidemic development through indirect pathways. The coupling effects of various factors across different spatiotemporal scales jointly shaped the unique spatiotemporal distribution and evolutionary characteristics of cholera epidemics.*

***Author summary***

*Cholera remains a major threat to public health, especially in regions with dense water networks. As a "province of a thousand lakes" in China, Hubei Province has a long history of cholera epidemics, but its long-term spatiotemporal patterns and driving forces were not fully understood. We studied cholera cases in Hubei Province from 1949 to 2020, combining historical records and environmental data with analytical tools like spatial analysis and statistical modeling. We found cholera was most severe in the 1980s–1990s, peaked in summer and autumn, and had three cycles (29, 19, 8 years). Spatially, it clustered in eastern Hubei Province along rivers/lakes, with its center shifting southwest over time. Population density and summer temperatures were the direct factors driving the outbreak of cholera epidemics, while river networks constituted the basic environmental background that facilitated epidemic transmission. Natural environmental factors such as altitude and precipitation, human environmental factors including road networks and economic conditions, as well as drought and flood disasters, could not only directly drive the spread of epidemics but also regulate the development of epidemics through indirect pathways.*

**Comment 1-2.**

**Introduction**

• The historical references to "Tiger Lera" and "Tiger Epidemic" are interesting cultural context but feel tangential to your analysis. Consider shortening or moving to a footnote.

• You cite extensive work on cholera in other regions but should strengthen the justification for why Hubei specifically matters. You mention it's a "province of a thousand lakes" but could emphasize:

(1) Hubei's position as a major transportation hub, (2) the Three Gorges Dam's impact on hydrology, (3) the 1998 Yangtze flood as a natural experiment, or (4) applicability to other monsoonal regions with dense river networks.

**Response 1-2.**

Thank you for your valuable feedback on the introduction section of the article. Below are specific revisions and responses to each point:

We sincerely appreciate your insightful suggestions for improving the introduction. Both points you raised are highly pertinent and will help enhance the section's academic focus and argumentative strength. Based on your recommendations, we have made the following revisions:

First, regarding streamlining historical references: We fully agree that historical aliases like "Tiger Lera" and "Tiger Epidemic" are more closely tied to cultural contexts and less central to the subsequent quantitative spatial analysis. To maintain conciseness, we have removed the phrase "which is also known as ‘Tiger Lera’ and ‘Tiger Epidemic’ in ancient Chinese texts".

Second, regarding the rationale for intensifying research on Hubei. The supplementary directions you proposed are highly valuable. We have strengthened the corresponding sections in the introduction to more fully articulate "why Hubei is an ideal and important case study region": After describing Hubei's characteristic as the "Province of a Thousand Lakes", we added emphasis on its status as a national transportation hub, the "crossroads of nine provinces", noting that this makes it a critical node for the introduction and domestic transmission of infectious diseases. When discussing the Yangtze River, we added how the Three Gorges Dam (located in Yichang, Hubei) provides unique context by altering the environmental substrate for waterborne infectious diseases. In addressing disaster factors, we explicitly cite the 1998 catastrophic Yangtze River floods (with Hubei among the hardest-hit areas), noting this provides a near-perfect natural experiment scenario for studying how extreme climate events trigger and shape cholera outbreaks. In concluding the research significance section, we specifically highlight that this in-depth analysis of Hubei—a prototypical region characterized by dense river networks, large-scale water conservancy projects, frequent flooding, and high-intensity human activity and transportation—holds significant reference value and theoretical implications for cholera prevention and control in other global monsoon climate zones, river deltas, lake-dense areas, and rapidly urbanizing regions. Specific revisions are as follows (P 3-4):

***Introduction***

*Cholera is one of China's two Class A statutory infectious diseases, a virulent intestinal infectious disease caused by the Vibrio cholerae[1]. In terms of spatiotemporal patterns, cholera was likely introduced into China by sea around the Jiaqing-Daoguang period (circa 1796-1850) of the Qing Dynasty. It first landed in Guangzhou and then gradually spread to coastal areas and inland regions [2]. After 1949, with the advancement of prevention and control measures, the national cholera incidence rate dropped significantly. However, there were still three epidemics peaks in the 1960s, 1980s, and 1990s. In 1993, predominantly caused by the El Tor biotype of Vibrio cholerae (a subtype of serogroup O1). It was not until the first local outbreak of O139 cholera (cholera caused by the O139 serogroup of Vibrio cholerae) occurred, and since then, O139 cholera epidemics have continued unabated [3]. As China's "Province of a Thousand Lakes" and a transportation hub in the middle reaches of the Yangtze River (renowned as the "Crossroads of Nine Provinces"), Hubei Province possesses unique regional representativeness for cholera research. Its dense network of rivers and lakes forms a natural environmental substrate for cholera transmission. The world-class Three Gorges Dam, located within the province, has significantly altered the hydrological conditions and ecological environment of the middle and lower reaches of the Yangtze River, potentially exerting profound impacts on the habitat of Vibrio cholerae. Simultaneously, as a core transportation node connecting China's east, west, north, and south, Hubei Province experiences frequent population and material flows, making the risks of infectious disease introduction and spread particularly prominent. Historically, Hubei Province had been hit by severe cholera epidemics on multiple occasions: in the first year of Xuantong (1909), those who died of cholera in Hankou were "omnipresent" [4]. In the 21st year of the Republic of China (1932), cholera occurred in 12 cities in Hubei Province, with over 1,200 people dying from it, and the mortality rate reached 43.5%[5]. Since 1949, especially since the year 2000, the incidence rate of intestinal infectious diseases such as cholera had shown a year-by-year downward trend[6]. The epidemics are mainly characterized by sporadic cases and small-scale outbreaks caused by group dinners [7, 8]. The incidence rises significantly in summer and autumn [9], and high-incidence areas are mostly concentrated in counties along rivers, such as the Jianghan Plain[10]. In terms of the formation mechanism, climate warming can to a certain extent lead to the transmission and spread of cholera [11]. As a waterborne disease, cholera is transmitted through contaminated well water and river water [12]. Hilly areas, mountainous areas, and plain areas are all high-incidence topographical regions [13, 14]. The development of transportation has provided conditions for the transmission of cholera [15]. Cholera often follows disasters. Among them, wars cause frequent outbreaks of cholera by destroying infrastructure, creating population displacement and resource scarcity [16]; floods significantly increase the risk of cholera epidemics by contaminating water sources, damaging sanitation systems, and exacerbating population vulnerability [17]. For example, the catastrophic 1998 floods in the Yangtze River basin heavily impacted Hubei—one of the most severely affected regions—and provided a valuable natural experiment for examining how extreme climate events trigger cholera epidemics.*

*However, existing studies still have some limitations: In terms of spatiotemporal patterns, analyses have mainly focused on the spatiotemporal patterns of cholera in some regions[18-22]. There is still a lack of geographical studies with long-term and high spatiotemporal resolution on regional cholera epidemics. In terms of formation mechanisms, there is still a lack of comprehensive quantitative studies on the formation mechanisms of regional cholera epidemics, and there is also insufficient identification of major influencing factors and analysis of their action mechanisms [18, 23]. In terms of cholera research in Hubei Province, there is a lack of specialized studies on cholera epidemics in this region, which is densely covered with rivers and lakes and has a severe history of epidemics. Therefore, to address these research gaps, this study aims to systematically answer three core scientific questions: (1) The long-term trends, cyclical fluctuations, and seasonal characteristics of cholera epidemics in Hubei Province from 1949 to 2020; (2) Its overall distribution patterns and spatial distribution changes; (3) The formation mechanisms of these spatiotemporal patterns shaped by multidimensional factors including natural conditions, disaster events, and socioeconomic factors. To this end, this study integrates time-series analysis, spatial statistics, and structural equation modeling to elucidate the complex mechanisms underlying regional cholera epidemics. It aims to provide scientific insights for cholera prevention and control in* *river- and lake-rich regions, particularly for other areas globally facing similar environmental conditions (e.g., monsoon climate, dense river networks) and socioeconomic pressures (e.g., rapid urbanization, high population mobility).*

**Comment 1-3.**

**Methods Section**

**Spatial Merging Method**: You describe merging historical counties to match 2020 boundaries, which is excellent practice. However, you need more detail:

•When counties split, how did you allocate historical case counts? Population-weighted? Areaweighted? Equal division?

• Appendix Table 2 lists boundary changes but doesn't explain your allocation method

• This is crucial for reproducibility

**Wavelet Analysis:**

• Why was the Morlet wavelet chosen over other mother wavelets (e.g., Daubechies, Mexican Hat)? Provide brief justification.

• What was the scale range used? How many decomposition levels?

• Figure 5's caption mentions "Real contour plot" but doesn't explain how to interpret the colors—what do the alternating red/blue zones represent in practical terms?

**Spatial Weight Matrix:**

• For spatial autocorrelation (Equation 4), you define W_ij as "the spatial weight of counties i and j" but never specify: Queen contiguity? Rook? Distance-based? First-order neighbors only? Please clarify.

**SEM Goodness of Fit:**

• Table 2 shows excellent fit indices, but with 103 observations and 11 variables, your sample size is relatively small for SEM (typically want n>200). Acknowledge this limitation.

**Response 1-3.**

Thank you for your valuable comments regarding the "Methodology" section of the manuscript. Below are the specific revisions and response plans addressing each point:

1.thank you for your attention to the spatial merging method. We fully agree with your perspective and have supplemented the detailed steps for allocating historical case numbers during administrative division changes in the Data Source section (P 5-6). The specific modifications are as follows:

*To ensure the spatial comparability of data across different periods, the study adopted the "spatial merging method for historical data": for county-level units that were abolished or merged between 1949 and 2020, their historical epidemic data were merged based on the 2020 county-level administrative divisions by comparing the changes in Hubei's administrative divisions during 1949-2020. Specific merging rules: For counties that were split—i.e., where a historical county-level unit was later divided into multiple 2020 county-level units—the number of cases was allocated according to the population proportion of the affected area in the year the epidemic occurred. Specifically, the most recent population census data for that epidemic year was obtained first. Then, the case numbers were weighted and allocated based on the proportion of the population in each post-split county relative to the total population of the original county. For example, if a county had a total population of 1 million in the epidemic year and was later split into County A (600,000 people) and County B (400,000 people), then 60% of the cholera cases reported for the original county would be assigned to County A and 40% to County B. For counties that underwent only name changes or jurisdictional adjustments with essentially unchanged boundaries, their historical data were directly matched to the 2020 county-level unit with the same name or covering the same geographical area. This method uses population as the allocation weight, based on the epidemiological characteristic that cholera transmission is closely related to population density and the frequency of human contact. All key time nodes of administrative division adjustments are detailed in "Appendix Table 2: Comparison of Adjustments to Hubei's County-Level Administrative Divisions (1949-2020)" to ensure data traceability.*

2.Thank you very much for your professional inquiries regarding the details of the wavelet analysis method. This has prompted us to clarify the rationale for parameter selection more clearly, ensuring transparency and reproducibility of the analytical process.

Fist, regarding the choice of the mother wavelet, this study employs the complex Morlet wavelet, primarily due to its good balance between the time and frequency domains. The Morlet wavelet is a non-orthogonal, exponentially decaying complex-valued wavelet that achieves an optimal compromise between time resolution and frequency resolution. It is particularly well-suited for analyzing time series such as cholera case counts, which exhibit non-stationarity and potential multi-scale periodic characteristics. Its oscillatory waveform better matches the gradual fluctuation patterns commonly observed in infectious disease data, enabling effective extraction of quasi-periodic oscillatory signals from the series. In contrast, the Daubechies family of wavelets is more adept at capturing abrupt changes and local features (e.g., edges) in signals, while the Mexican hat wavelet (Ricker wavelet) has relatively weaker frequency-domain resolution and is more sensitive to noise. Therefore, the Morlet wavelet is more aligned with the objective of this study, which aims to detect medium- to long-term periodic fluctuations.

Second, regarding the scale range and number of decomposition levels: This study has redefined the scale range to 0 to 35 years. This range is designed to cover the main periodic fluctuations of cholera epidemics that are shorter than the total length of the time series (72 years). Setting the upper limit to 35 years is sufficient to detect potential decadal-scale fluctuations while avoiding statistical unreliability issues associated with periods that are too long (approaching the length of the series), as previously discussed for the 55-year cycle. Within the framework of continuous wavelet transform, we performed logarithmically equally spaced sampling across the 0–35 year range by setting 64 decomposition levels (or scale numbers). Selecting 64 levels ensures sufficiently dense and smooth scale sampling within this range, guaranteeing the continuity and precision of the periodic spectrum. This enables reliable identification of the locations of dominant cycles (e.g., 29-year, 19-year, and 8-year periods) and avoids missing important periodic information due to overly sparse scale sampling. We have supplemented the explanation for the above rationale and specific parameters in the "Research Methods", "Wavelet Analysis" section of the manuscript (P 11-12). The specific modifications are as follows:

***Wavelet Analysis.*** *Wavelet Analysis is a new branch of mathematics formed in the late 1980s, which is widely used in many fields such as signal processing because of its good time-frequency localization analysis capability [46]. The basic idea of wavelet analysis is to use a cluster of wavelet function systems to represent or approximate a signal or function. The wavelet function is the key to wavelet analysis, and there are many wavelet functions that could be selected. This paper selectes the Morlet wavelet function, primarily based on two considerations. First, the Morlet wavelet exhibits good localization properties in both the time and frequency domains, allowing for an optimal balance between temporal resolution and frequency resolution. This makes it particularly suitable for analyzing non-stationary time series such as cholera case counts[27, 44, 45]. Second, its oscillatory waveform better matches potential quasi-periodic fluctuation signals within the series, enabling effective extraction of latent periodic components. Compared to the Daubechies wavelet, which is more adept at capturing abrupt changes, or the Mexican hat wavelet (Ricker wavelet), which has relatively weaker frequency-domain resolution, the Morlet wavelet is better aligned with the analytical objectives of this study. In terms of parameter settings, continuous wavelet transform was employed, with the scale range corresponding to periods from 2 to 35 years, to detect potential fluctuations from short-term to long-term cycles. The scales were densely sampled using a logarithmic equal-interval approach across 64 scale levels to ensure the continuity of the periodogram, thereby enabling precise identification of the dominant cycles in the series. By computing the real part of the wavelet coefficients, the oscillation phase and intensity of the series across different time scales can be analyzed; meanwhile, calculating the wavelet variance allows for the determination of the dominant periodic scales. In this paper, Matlab software’s wavelet analysis tool was used to calculate the real part of wavelet coefficients and wavelet variance of the number of cholera cases in Hubei Province, and Surfer software was used to draw the contour map of the real part of wavelet coefficients.*

Third, regarding your comments on the real-part contour plot of wavelet coefficients (Fig. 5), the issue of incomplete legend information you pointed out is indeed critical. The interpretation of the color coding is essential for readers to correctly understand the periodic oscillation characteristics revealed by the figure. Following your suggestion, we have added more direct interpretive guidance when describing Figure 5 in the "Results" section, under the "Periodic Variations" subsection. The specific modifications are as follows(P 16-17):

***Cyclical variations***

*The real part of the Morlet wavelet transform coefficients reflects the fluctuation characteristics of data within the wavelet domain across different time scales. The alternating positive and negative values of the real part correspond to the severity characteristics of the cholera sequence in the time domain. Plotting the isocontour map of the real part of the wavelet transform coefficients for the cholera epidemic sequence (Fig 5), the colors represent the sign and magnitude of the real part of the wavelet coefficients: red regions indicate positive real parts of the wavelet coefficients, corresponding to periods of elevated cholera case numbers; blue regions indicate negative real parts of the wavelet coefficients, corresponding to periods of reduced case numbers. It can be observed that the cholera epidemics in Hubei Province exhibited different periodic variations and alternating high-low incidence processes across different time scales, forming oscillation centers with alternating positive and negative values at various scales. Overall, the cholera epidemics in Hubei Province from 1949 to 2020 showed fluctuation cycles at three time scales: 25-35a, 16-22a, and 5-10a. Specifically: At the 25-35a time scale, there were 4 quasi-oscillation cycles with alternating high and low incidence, which were relatively stable and exhibited full-domain coverage throughout the entire study period. At the 16-22a time scale, there were 4 quasi-oscillation cycles with alternating high and low incidence, which mainly occurred between 1960 and 2005. At the 5-10a time scale, there were 4 quasi-oscillation cycles with alternating high and low incidence, which mainly occurred between 1980 and 1999.*

3.Thank you for your valuable feedback regarding the spatial weight matrix. We have now explicitly specified the type of spatial weight matrix in the revised manuscript: when generating the spatial weight matrix, the inverse distance was adopted to define spatial relationships. This information has been added to the "Spatial Autocorrelation Analysis" subsection under the "GIS Spatial Analysis" section (P 13). The specific revisions are as follows:

***GIS Spatial Analysis.***

*……*

*Where n represented the number of spatial units, i.e., 103 counties in Hubei Province; xᵢ and xⱼ referred to the number of cholera cases in counties i and j, respectively;* $\bar{X}$ *represented the mean number of cholera cases in all counties; and Wᵢⱼ was the spatial weight of counties i and j. In this study, the ArcGIS "spatial autocorrelation" tool was used to calculate Moran’s I for global spatial autocorrelation analysis, and the "clustering and outlier analysis" tool was used to analyze local spatial clustering patterns, and when generating the spatial weight matrix, the inverse distance was adopted to define spatial relationships.*

4.Thank you for pointing out the sample size issue. We fully agree with your perspective and have acknowledged this limitation in the "Discussion" section. The text explicitly states: "The sample size for the structural equation model in this study was 103 county-level units. While meeting the basic requirements for model fitting, it remains relatively small compared to generally recommended sample sizes (typically n > 200), which may affect parameter stability to some extent." We have placed this clarification within the "Study Limitations" subsection (P 35) to enhance the transparency and rigor of the research. The specific revision is as follows:

***Discussion***

*……*

***Research Limitations***

*……*

*(3) Structural Equation Modeling. The sample size for the structural equation model in this study comprised 103 county-level units. While meeting the basic requirements for model fitting, it remains relatively small compared to the generally recommended sample size (typically n>200). This might have somewhat limited the stability and generalization capability of the model parameters. Future studies utilizing longer time series or higher spatial resolution data could enhance the model's robustness and interpretability.*

**Comment 1-4.**

**Results Section**

**Figure Quality and Clarity**:

• Figure 3: The 1988 peak (1,351 cases) dominates the y-axis scale, making other years difficult to distinguish. Consider a log scale, broken axis, or an inset zoom for 1949-1987 and 1989-2020.

• Figure 5 (wavelet contours): Font sizes for axis labels are quite small. Ensure these will be legible in print.

• Figure 8: Use consistent color schemes. The graduated color in 8a (case counts) differs from 8b (Moran's I), which can confuse readers.

• Figure 9: Excellent time-series maps, but consider adding the migration path of the center of gravity as a line with arrows directly on panel (f) for easier visualization.

• Figure 10: The path diagram would benefit from including path coefficient values directly on the arrows, not just in Table 3.

**Endogenous vs. Exogenous Variables Results**: The results section jumps between describing endogenous variables (population density, temperature, river networks) and exogenous variables (elevation, precipitation, floods, etc.) in Table 3. Consider reorganizing with clear subheadings: "Direct Effects of Endogenous Variables" and "Indirect Effects of Exogenous Variables" to improve readability

**Response 1-4.**

Thank you for your valuable feedback on the "Results" section of the article. Below are specific revisions and responses to each point:

1.Thank you for your valuable and specific suggestions for improving the charts. These recommendations are crucial for enhancing the paper's visual appeal, the clarity of data presentation, and the reader's experience. We have carefully reviewed and revised all relevant charts based on each of your suggestions. The specific modifications are as follows:

**Fig. 3 Changes in the number of cholera cases in Hubei Province from 1949 to 2020:** We fully agree that the original Figure 3 obscures the trend in subsequent years due to the extreme peak in 1988. In the revised version, we have redrawn the figure by adding enlarged insets for the periods 1949–1987 and 1989–2020. The new version retains the 1988 peak while clearly illustrating the subtle fluctuations in cholera case numbers during the low-level oscillation phase after 1989, significantly enhancing the clarity and scientific rigor of the results presentation.

**Fig. 5 Real contour plot of wavelet transform coefficients of cholera caces in Hubei Province:** We have increased the font size and weight of axis labels, tick marks, and legends to ensure they are clearly legible both in print and on screen.

**Fig. 8 The overall distribution characteristics of cholera epidemics in Hubei Province from 1949 to 2020:** Thank you for pointing out the inconsistency in the color scheme. We have standardized the color schemes in Figures 8a and 8b to avoid visual confusion.

**Fig. 9 Cholera epidemics in Hubei Province by chronology from 1949 to 2020:** Figure 9(f) in the initial submission version already depicted the migration path of the disaster's center of gravity, but the visual distinctiveness of the arrow indicating the potential path was insufficient. To more clearly and intuitively present the spatiotemporal direction of the center's shift, the revised version has enlarged the path arrow, thereby enhancing the graphic's readability and information transmission efficiency.

**Fig. 10 Mechanism and transmission path of influencing factors of cholera epidemics in Hubei Province：**We have directly labeled standardized path coefficients above each arrow in the path diagram. This fully aligns with your request, enabling readers to interpret key quantitative relationships within the model directly without consulting tables.

2.We sincerely appreciate your valuable suggestions regarding the section structure of "The Formation Mechanism of Cholera Epidemics." Upon careful review, we note that while our previous presentation of structural equation modeling results followed the logical framework of "endogenous variables" and "exogenous variables," the subheadings may not have sufficiently highlighted this framework. To enhance the clarity and logical hierarchy of the chapter structure, we have revised the original headings to: "***Formation Mechanisms of Endogenous Variables in Cholera Epidemics***" and "***Epidemics Formation Mechanisms of Exogenous Variables in Cholera Epidemics***".

**Comment 1-5.**

**Discussion Section**

**Repetition of Results:** The Discussion extensively repeats results already presented. For example, the first paragraph of "Summary of cholera epidemics formation mechanisms" largely restates Table 3 findings. The Discussion should focus on interpretation and contextualization rather than repetition. Consider:

• Comparing your findings to global cholera research

• Discussing implications for climate change

• Addressing why your findings might differ from coastal cholera patterns

Global Cholera Context: You cite some international studies but could strengthen connections to:

• Vibrio cholerae ecology and environmental survival (temperature, salinity, pH)

• Climate-driven cholera outbreaks in Bangladesh, Africa, and elsewhere

• The global shift from water-borne to food-borne transmission

• El Niño-Southern Oscillation (ENSO) effects on cholera globally

**The "Double Holiday" Hypothesis:** The discussion of Mid-Autumn Festival and National Day (September-October) as cholera risk periods is interesting but speculative. You state "cholera epidemic peaks in Hubei Province are significantly associated with meal gatherings during the Mid-Autumn Festival and National Day holidays," but you haven't shown data demonstrating peaks specifically during these holidays versus other September-October periods. Either provide temporal data showing holiday-specific clustering or tone down this claim.

**Policy Recommendations:** Your Hubei-specific recommendations are creative and locally relevant:

• Sluice biosurveillance stations at Yangtze/Han River entry points

• "Cholera prevention ecological corridors"

• "Double-Non" certification for restaurants

However, these need feasibility consideration:

• Biosurveillance stations require significant infrastructure and ongoing maintenance—are they costeffective given the declining cholera incidence?

• How would "ecological corridors" be implemented practically?

• The restaurant certification idea is excellent but needs integration with existing food safety regulations

Consider adding a brief sentence on implementation challenges or resource requirements.

**Climate Change Implications:** You mention climate warming in the Introduction and use temperature as a key variable, but never discuss future implications. With climate change potentially increasing flooding frequency and altering summer temperatures in Hubei, what are the implications for future cholera risk? This deserves at least a brief paragraph

**Response 1-5.**

Thank you for your valuable feedback on the "Discussion" section of the article. Below are specific revisions and responses to each point:

1.Thank you very much for your highly constructive and insightful critique and guidance regarding the "Discussion" section. We fully agree with your core point that the "Discussion" should focus on interpretation, contextualization, and synthesis, rather than reiterating the "Results". The specific directions you highlighted—such as global comparisons, implications for climate change, regional pattern differences, and strengthening the global ecological context of cholera—have provided a clear blueprint for us to deepen the discussion section. In the "Discussion" section, under the subsection "Research Extension and Comparison," we have: First, supplemented our findings from Hubei with a global perspective by comparing the mechanisms observed in cholera-affected regions such as Bangladesh and Africa. This approach affirms the universality of temperature-driven mechanisms while highlighting that Hubei, as an inland freshwater lake region, exhibits fundamentally different driving patterns compared to coastal/estuarine areas influenced by salinity and sea surface temperature. Second, we discuss the increase in foodborne outbreaks in Hubei since the 1980s, interpreting this within the broader global trend of cholera transmission shifting from "waterborne" to "foodborne" pathways. This emphasizes the role of urbanization and food supply chains in modern cholera transmission. Third, we explored potential links between the cycles identified in this study and climate modes such as El Niño, situating regional epidemic dynamics within the framework of the global climate system. Specific revisions are as follows(P 28-32):

***Discussion***

***Research Extension and Comparison***

*Temporal Variations in Cholera Epidemics in Hubei Province.……In addition, this study identified three significant cholera epidemic cycles of 29, 19, and 8 years, which may reflect the coupling of multi-scale driving processes. The 29a cycle aligned with the epidemiological cycle of "introduction-epidemic-suppression" for major Vibrio cholerae biotypes (e.g., the El Tor biotype in the 1960s) and concurrent socio-economic transformations (e.g., the Reform and Opening-up period), suggesting it was a composite biosocial cycle shaped jointly by pathogen, host, and social factors. The 19a cycle corresponded closely to the activity period of low-frequency climate modes such as the Pacific Decadal Oscillation, potentially modulating regional climatic and hydrological backgrounds and setting an environmental rhythm at the decadal scale for epidemics. The 8a cycle was associated with interannual climate variability such as the El Niño-Southern Oscillation and the triggering frequency of flood disasters, reflecting the role of extreme weather events as direct "triggers" for outbreaks. Together, these cycles constituted the comprehensive temporal characteristics of cholera epidemics under multiple mechanisms, including long-term pathogen evolution, medium-term climatic fluctuations, and short-term disaster triggers.*

*……*

*Regarding the comparison of this study’s findings with other research. Fist, the driving mechanisms of cholera in Hubei Province revealed in this study not only align with the general patterns of global cholera epidemics but also exhibit distinct characteristics due to environmental differences between inland water networks and coastal brackish water convergence zones. This study found that the average summer temperature was one of the strongest environmental factors driving cholera epidemics. This finding was highly consistent with observations from major global cholera-endemic regions. In Bangladesh, rising sea surface temperatures had been confirmed as a key driver of seasonal epidemics and interannual outbreaks; in the African Great Lakes region, temperature increases following the rainy season often coincided with epidemic outbreaks. Together, these findings corroborated the core role of temperature as a "universal switch" regulating the proliferation rate of Vibrio cholerae in aquatic environments [17, 21]. However, the uniqueness of this case lied in its purely inland freshwater environment. Unlike coastal and estuarine epidemic areas (such as the Bay of Bengal), which were strongly influenced by salinity gradients and tidal effects, the river and lake networks in Hubei Province exhibited stable and extremely low salinity. Our model indicateed that the direct impact of river network density itself was weaker, suggesting that in freshwater systems, dense water networks provided the necessary medium for transmission but are not a sufficient condition determining epidemic intensity. The spatial differentiation of epidemics was primarily determined by the degree of population aggregation and climatic suitability windows superimposed on this hydrological background. This "hydrological base + socio-climatic amplifier" mechanism provideed a theoretical framework for understanding cholera risks in other inland water-rich regions (such as large lake basins). Second, the transmission pathways of cholera were undergoing a global evolution. The increased records of outbreaks triggered by group gatherings in Hubei Province since the 1980s, as observed in this study, were not an isolated phenomenon. Instead, they reflected a global trend shifting from traditional "waterborne transmission" dominance toward an increasing importance of "foodborne transmission" [54, 56]. As urban and rural water supply and sanitation infrastructure improve, the proportion of outbreaks directly caused by drinking water contamination had declined. However, the lengthening of food supply chains and the frequent occurrence of group dining activities driven by globalization and urbanization had created new pathways for Vibrio cholerae cross-contamination through food. The case of Hubei Province demonstrated that even in inland regions, socio-economic development and lifestyle changes were reshaping the transmission dynamics of infectious diseases. This necessitated that modern cholera prevention and control must transcended a singular environmental sanitation perspective and incorporate systematic supervision of food supply chain safety and collective dining hygiene.*

2. Thank you very much for pointing out that our original statement regarding "the Mid-Autumn Festival and National Day (the dual holidays) being a cholera risk period" was speculative and failed to provide direct evidence distinguishing between "seasonal risk (September-October)" and "holiday-specific risk." Accordingly, we have substantially revised the relevant statements to align with the level of support provided by existing data. In the discussion section, we have modified the original overly definitive assertion—"The peak of cholera epidemics in Hubei Province is significantly associated with group dining activities during the Mid-Autumn Festival and National Day holidays"—to a more rigorous and data-driven formulation(P 29-30):

***Discussion***

***Research Extension and Comparison***

*Temporal Variations in Cholera Epidemics in Hubei Province……Next, this study found that cholera epidemics peaked between August and October, coinciding with the warm and humid summer-autumn seasons in Hubei Province. Against this seasonal high-risk background, large-scale family and social gatherings were frequent, particularly around the Mid-Autumn Festival and National Day. Existing research has demonstrated that inadequate food hygiene supervision at such collective dining events can serve as critical nodes for foodborne cholera outbreaks[52,54]. The epidemic records collected in this study also contain explicit documentation of cases linked to mass gatherings [55]. Therefore, although the temporal resolution of the data precluded the statistical isolation of the specific risk associated with the holiday days themselves, the intensive dining culture during the "two festivals" likely overlapped with the inherent climatic risk period, jointly shaping the epidemic peak pattern observed from August to October.*

3. Thank you very much for your positive feedback on the policy recommendations we proposed, as well as for your valuable insights regarding their practical feasibility. The issues you raised—such as cost-effectiveness, practical implementation, and integration with existing regulations—are indeed core challenges that must be addressed for any public health intervention to move from conception to practice. We fully agree with your perspective and have supplemented the discussion on the implementation considerations and feasibility of these recommendations in the "Discussion" section, under the subsection on policy recommendations (P 32-34). The specific modifications are as follows:

***Policy Recommendations***

*To effectively prevent and respond to future cholera outbreaks, based on research into the spatio-temporal patterns and formation mechanisms of cholera epidemics in Hubei Province, the following Hubei-specific prevention and control recommendations are proposed. These offer particular reference value for regions with dense river and lake networks: (1) Risk management during key periods: Focus should be placed on the high-incidence summer and autumn seasons, especially August and September. Based on the main cycles of 55 years and 29 years for cholera in Hubei Province , preventive measures should be taken in advance in predicted peak years. (2) Establish a climate-adaptive early warning and response system. Given the critical driving role of climate conditions in this research model, future global warming will prolong summer heatwaves and increase the frequency of extreme precipitation events. This is highly likely to extend the "risk window" for cholera transmission and increase outbreak frequency. Therefore, the prevention and control system must be climate-adaptive. Seasonal risk outlooks based on climate forecasts could be issued each spring. During El Niño development phases, advance assessments of their potential impact on the following year's epidemic should be conducted. Flood disaster warnings should be directly linked to cholera emergency surveillance activation mechanisms, enabling enhanced monitoring of water bodies and diarrheal symptoms in key areas within 72 hours post-disaster. (3) Hierarchical spatial management and control: Level-1 risk zones (Wuhan urban area, 3 counties along the Yangtze River in Xianning): Implement sluice biosurveillance stations, install automatic sampling and testing equipment at the sluices of the Yangtze River/Han River. When vibrio levels exceed standards, trigger the closure of ecological gates to block the invasion of polluted water into urban inland lakes. To reduce implementation costs, this system can be integrated with existing water quality monitoring stations and water conservancy hubs operated by water resources departments. By sharing station locations, power supplies, and communication networks, it achieves functional convergence of hydrological and pathogen monitoring, forming a "multi-functional integrated monitoring platform". Level-2 risk zones (other counties and cities in the Jianghan Plain): Incorporate water vibrio concentration into the assessment system of the river chief system; suspend fishery and tourism projects in river sections that fail to meet standards. This strategy can be directly embedded into China's existing "River and Lake Chief System" governance framework. Implementation requires only adding assessment indicators without creating new administrative bodies, offering significant advantages of low administrative resistance and high synergistic benefits. Level-3 risk zones (western Hubei Province mountainous areas): Set up quarantine stations at inter-county transportation hubs (such as the Three Gorges Dam in Yichang) to conduct random inspections on freight vehicles from historical epidemic areas. Leveraging existing joint enforcement mechanisms between transportation and agriculture departments, deploy additional health quarantine personnel during high-risk summer and autumn periods. This creates a flexible prevention model combining routine patrols with peak-period monitoring, enabling low-cost, high-efficiency interception of imported risks. (4) Joint prevention and control at transportation hubs: Relying on hubs such as Wuhan Tianhe Airport and Hankou Railway Station, establish a mechanism of "symptom monitoring for personnel entering Hubei Province and rapid testing of water samples in luggage". Implement "on-arrival testing" for flights and trains from cholera-affected areas, and establish a "real-time case information sharing platform" with surrounding provinces to block the transmission chain of imported epidemics. (5) Ecological management and control of rivers and lakes: Establish "cholera prevention and control ecological corridors" in key river sections such as the Wuhan section of the Yangtze River and the Xiangyang section of the Han River. It is strictly prohibited to discharge domestic sewage into rivers during the flood season. Complete the construction of "regular monitoring points for Vibrio cholerae" at all drinking water sources at or above the county level by May each year, and implement closed-loop management of "supply suspension—disinfection—re-inspection" for water bodies that exceed standards. Furthermore, "ecological corridor" development should be deeply integrated with the national "Yangtze River Protection" strategy and local "Beautiful Rivers and Lakes" initiatives, translating pathogen control targets into specific ecological design parameters for wetland restoration and shoreline remediation projects. Monitoring station construction can be incorporated into routine fiscal safeguards for drinking water sanitation.* (6) Establish a Safe Drinking Water Assurance System. As the most fundamental public health measure for cholera prevention and control, ensuring drinking water safety requires systematically enhancing water safety assurance capabilities beyond existing environmental monitoring. This includes improving water treatment and disinfection capacity to guarantee the stable effectiveness of chlorination and other processes. During early warning periods, temporarily increase residual chlorine levels in treated water before distribution and intensify testing of terminal pipelines in older urban areas. Addressing shortcomings in rural decentralized water supply, advance projects to consolidate and enhance drinking water safety. Promote small-scale water purification facilities and household disinfection equipment, while strengthening training for water management personnel and users on water quality assessment and equipment maintenance. *(7) Hubei's unique breakfast culture of eating breakfast on the street carries the risk of contamination from blanched raw foods. It is recommended to promote the "Double-Non" certification (non-river-water dishwashing, non-raw/cold ingredients) in scenic spots such as Hubu Lane in Wuhan, and incorporate it into the evaluation criteria for Hubei's (Jingchu) dietary intangible cultural heritage. This certification system can be embedded within the market supervision department's existing "quantitative grading for food safety" dynamic evaluation, serving as a bonus point or specialty certification for "transparent kitchens". Market reputation incentives can encourage voluntary participation by businesses, avoiding additional mandatory administrative burdens.*

4. We sincerely appreciate your valuable suggestion regarding the "Climate Change Implications". We fully concur that when discussing future cholera risks, it is essential to proactively consider the potential impacts of climate change. While we previously mentioned global warming in the introduction, we indeed failed to delve into its future implications. To address this, we have systematically supplemented the "Policy Recommendations" subsection within the "Discussion" section with an analysis of how climate change will affect future cholera risks and corresponding countermeasures. The specific revisions are as follows(P 32-33):

***Policy Recommendations***

*To effectively prevent and respond to future cholera outbreaks, based on research into the spatio-temporal patterns and formation mechanisms of cholera epidemics in Hubei Province, the following Hubei-specific prevention and control recommendations are proposed. These offer particular reference value for regions with dense river and lake networks: …… (2) Establish a climate-adaptive early warning and response system. Given the critical driving role of climate conditions in this research model, future global warming will prolong summer heatwaves and increase the frequency of extreme precipitation events. This is highly likely to extend the "risk window" for cholera transmission and increase outbreak frequency. Therefore, the prevention and control system must be climate-adaptive. Seasonal risk outlooks based on climate forecasts could be issued each spring. During El Niño development phases, advance assessments of their potential impact on the following year's epidemic should be conducted. Flood disaster warnings should be directly linked to cholera emergency surveillance activation mechanisms, enabling enhanced monitoring of water bodies and diarrheal symptoms in key areas within 72 hours post-disaster.*

**Comment 1-6.**

**Writing and Language**

Overall Quality: The English is generally good but needs light editing for grammar, flow, and conciseness. Some sentences are overly long or read as literal translations from Chinese.

**Specific Examples:**

• Abstract: "The rapid spread and high lethality of the disease not only cause large numbers of deaths and injuries, but also have far-reaching effects by disrupting production, increasing the burden of health care and impeding economic exchanges." → This is overly dramatic for an abstract. Simplify.

• Methods: Many sentences exceed 40 words (e.g., the spatial merging method paragraph). Break these into shorter, clearer sentences.

• Results: "Temporally, high incidence occurred in the 1980s and 1990s, with summer and fall being the dominant seasons." → Use "autumn" instead of "fall" for consistency with international readership.

**Terminology Consistency:**

• Use "Hubei Province" consistently (avoid mixing "in Hubei," "of Hubei," etc.)

• "Cholera epidemics" vs. "cholera epidemic"—ensure singular/plural consistency

• "O139 cholera" appears in the Discussion without prior definition—define it when first mentioned

**Technical Terms:**

• Define "El Tor biotype" when first mentioned (Introduction or early Discussion)

• Explain "O139 cholera" for non-specialist readers

• The term "Tiger Lera" is interesting but unexplained—is "Lera" a transliteration of cholera?

Clarify

**Response 1-6.**

Thank you for your valuable feedback on the article "Writing and Language". Below are specific revisions and responses to each point:

1. Thank you for your valuable suggestions regarding the article. We have systematically proofread and revised the content.

First, as you pointed out, the background section was overly exaggerated. We have simplified it to (P 2):

***Abstract***

*Background: The global epidemics of cholera, a virulent enteric infection, poses a serious threat to public health and socio-economics. The disease’s rapid spread and high mortality has led to heavy casualties, along with disruptions to production, rising healthcare burdens and impaired economic exchanges.*

Second, We split long sentences in the Methodology section into shorter, clearer ones. The specific revisions are as follows（P 6、12-13）：

***Data source***

*(1) Cholera epidemics data. ……Specific merging rules: For counties that were split—i.e., where a historical county-level unit was later divided into multiple 2020 county-level units—the number of cases was allocated according to the population proportion of the affected area in the year the epidemic occurred. Specifically, the most recent population census data for that epidemic year was obtained first. Then, the case numbers were weighted and allocated based on the proportion of the population in each post-split county relative to the total population of the original county. For example, if a county had a total population of 1 million in the epidemic year and was later split into County A (600,000 people) and County B (400,000 people), then 60% of the cholera cases reported for the original county would be assigned to County A and 40% to County B. For counties that underwent only name changes or jurisdictional adjustments with essentially unchanged boundaries, their historical data were directly matched to the 2020 county-level unit with the same name or covering the same geographical area. This method uses population as the allocation weight, based on the epidemiological characteristic that cholera transmission is closely related to population density and the frequency of human contact.*

***Methodology***

*……*

***Geographic Information System (GIS) Spatial Analysis.*** *(1) Spatial Autocorrelation Analysis. Spatial Autocorrelation Analysis can measure the spatial distribution pattern of an outbreak based on the similarity of spatial locations and attributes. It can also detect spatial clustering and anomalies, including both global and local spatial autocorrelation.*

*……*

*（2） Distributional Center of Gravity Change Analysis. Referring to the population center of gravity model [47], the center of gravity of epidemic distribution is the point in the spatial plane where moments reach equilibrium, based on the distribution of cases in a given region. The equilibrium of the epidemic distribution can be assessed by comparing this center of gravity with the geometric center of the region.*

Third, we replaced "fall" with "autumn" throughout the article to maintain consistency.

2. Thank you for your valuable suggestions regarding terminology consistency and professional jargon in the article. We have conducted systematic proofreading and revisions to ensure professional and unified expression throughout the text.

First, "Hubei Province" is uniformly used as the standard designation to maintain consistency and avoid ambiguity.

Second, we have reviewed the singular and plural forms of "cholera epidemics" and "cholera epidemic" throughout the text. The choice between "epidemic" and "epidemics" depends on whether the context refers to the overall epidemic phenomenon or specific multiple outbreak events.

Third, brief definitions have been added for "*O139 cholera*" and "*El Tor biotype of Vibrio cholerae*" in the "Abstract" section upon their first mention, to accommodate readers not specializing in infectious diseases. The specific revisions are as follows(P 3):

***Introduction***

*……*

*After 1949, with the advancement of prevention and control measures, the national cholera incidence rate dropped significantly. However, there were still three epidemics peaks in the 1960s, 1980s, and 1990s, predominantly caused by the El Tor biotype of Vibrio cholerae (a subtype of serogroup O1). It was not until 1993 that the first local outbreak of O139 cholera (cholera caused by the O139 serogroup of Vibrio cholerae) occurred, and since then, O139 cholera epidemics have continued unabated .*

Fourth, as mentioned in "Response 1-2" above, historical aliases such as "Tiger Lera" and "Tiger Epidemic" are more closely tied to cultural contexts and less central to the subsequent quantitative spatial analysis. To maintain conciseness, we have streamlined the text by omitting the phrase "which is also known as ‘Tiger Lera’ and ‘Tiger Epidemic’ in ancient Chinese texts".

**Comment 1-7.**

**Tables and Figures**

**Table 1 (VIF):**

• The checkmarks in the "criteria" column are unnecessary in a scientific paper. Just state in the table caption: "All VIF values <10 and tolerance >0.1, indicating acceptable multicollinearity."

**Table 2 (SEM Fit):**

• Excellent presentation. No changes needed.

**Table 3 (SEM Effects)**: This is your most important table but has some issues:

• The asterisk notation (*, **, ***) is good, but add it to the caption, not just the note

• Consider bolding significant values for easier identification

• The "overall," "direct," and "indirect" terminology needs explanation in the table caption for readers unfamiliar with SEM

• Some readers might prefer separate tables for endogenous and exogenous variables

**Figure 2 (Theoretical Framework):**

• Good conceptual diagram, but it doesn't show expected direction of effects (positive/negative)

• Consider using + and - symbols on arrows to indicate hypothesized relationships

• This would help readers follow your logic before seeing results

**Figure 11:** This comparison figure is excellent and crucial for understanding your "Summary of cholera epidemics formation mechanisms." However, it appears very late (page 22) after the Discussion has already begun. Consider moving it earlier or referencing it more prominently in the Results section when discussing temporal patterns.

**Response 1-7.**

Thank you for your valuable suggestions regarding the article's figures and tables. Regarding the figures, we will implement the following key modifications:

**1. Table 2. Results of Variance Inflation Factor (VIF) and Tolerance Tests (P 22):** Remove the checkmark from the "Standard" column. The text explains that all VIF values < 10 and tolerances > 0.1 indicate multicollinearity within an acceptable range. "The test results (Table 2) showed that the VIF values for all independent variables were below 10, and the tolerance values were all greater than 0.1."

**2. Table 4. Direct, indirect, and overall effects of cholera epidemics in Hubei Province (P 24-25):** First, all effect values significant at the p<0.05 level (i.e., values marked with an asterisk) have been bolded to facilitate rapid identification of key pathways by readers. Second, we carefully considered presenting the effect decomposition results for endogenous and exogenous variables in separate tables. However, we believe the current integrated single-table format better serves the paper's purpose. This is primarily because one of the core findings of this study is to reveal the formation mechanism of cholera epidemics under the combined influence of endogenous and exogenous variables, and the indirect effects of exogenous variables are calculated based on the direct effects of endogenous variables. Presenting all effect values—including direct, indirect, and total effects—within a single table provides readers with the most direct and comprehensive overview. This facilitates intuitive comparisons of the relative impact strength and causal pathways across different factors, whether endogenous or exogenous.

***Table 4. Direct, indirect and overall effects of cholera epidemics in Hubei Province.***

| ***Factor*** | ***Effect*** | ***Annual mean summer temperature*** | ***River network density*** | ***Population density*** | ***Cholera prevalence level*** |
| --- | --- | --- | --- | --- | --- |
| *Annual mean summer temperature* | *overall* | *-* | *-* | *-* | ***0.807^*^*** |
| *River network density* | *overall* | *-* | *-* | *-* | *0.013* |
| *Population density* | *overall* | *-* | *-* | *-* | ***0.837^***^*** |
| *Mean elevation* | *overall* | ***-1.221^***^*** | ***-0.400^***^*** | ***0.140^***^*** | ***-0.116^*^*** |
|  | *direct* | ***-1.221^***^*** | ***-0.400^***^*** | ***0.140^***^*** | ***0.757^*^*** |
|  | *indirect* | *-* | *-* | *-* | ***-0.873^*^*** |
| *Annual mean precipitation* | *overall* | ***0.087^***^*** | *-0.031* | *0.041* | *0.115* |
|  | *direct* | ***0.087^***^*** | *-0.031* | *0.041* | *0.011* |
|  | *indirect* | *-* | *-* | *-* | *0.104* |
| *Annual mean sunshine* | *overall* | ***-0.082^**^*** | *-0.123* | *0.054* | *0.110* |
|  | *direct* | ***-0.082^**^*** | *-0.123* | *0.054* | *0.133* |
|  | *indirect* | *-* | *-* | *-* | *-0.023* |
| *Flood disaster* | *overall* | *0.040* | *-0.109* | *0.039* | ***0.417^***^*** |
|  | *direct* | *0.040* | *-0.109* | *0.039* | ***0.353^***^*** |
|  | *indirect* | *-* | *-* | *-* | ***0.064^***^*** |
| *Drought disaster* | *overall* | ***-0.080^***^*** | *-0.140* | *0.045* | *-0.146* |
|  | *direct* | ***-0.080^***^*** | *-0.140* | *0.045* | *-0.117* |
|  | *indirect* | *-* | *-* | *-* | *-0.029* |
| *GDP* | *overall* | *-0.013* | *-0.136* | ***0.089^*^*** | *0.024* |
|  | *direct* | *-0.013* | *-0.136* | ***0.089^*^*** | *-0.038* |
|  | *indirect* | *-* | *-* | *-* | *0.062* |
| *Night Lights* | *overall* | *0.100* | *0.119* | ***-0.169^*^*** | ***1.080^***^*** |
|  | *direct* | *0.100* | *0.119* | ***-0.169^*^*** | ***1.139^***^*** |
|  | *indirect* | *-* | *-* | *-* | ***-0.059^***^*** |
| *Road density* | *overall* | *-0.060* | ***0.591^*^*** | ***1.069^***^*** | ***0.102^***^*** |
|  | *direct* | *-0.060* | ***0.591^*^*** | ***1.069^***^*** | ***-0.752^***^*** |
|  | *indirect* | *-* | *-* | *-* | ***0.854^***^*** |

*Note: Values are standardized regression coefficient estimates; *** indicates that the coefficient is significant at the 0.001 level, ** indicates that the coefficient is significant at the 0.01 level, and * indicates that the coefficient is significant at the 0.05 level.*

Third, we have explained the meanings and calculation methods of "overall effect", "direct effect", and "indirect effect" in the "Structural Equation Model" subsection of the "Research Methods" section, as well as at the beginning of the "Formation Mechanisms of Endogenous Variables in Cholera Epidemics" and "Formation Mechanisms of Exogenous Variables in Cholera Epidemics" subsections of the "Results" section, to facilitate understanding (P 14、23、25).

***Methodology***

*……*

***Structural Equation Model (SEM).*** *…… Among these, the effect value can be further decomposed into direct and indirect effects. Table 4 presents precisely this decomposition, interpreted as follows: The direct effect refers to the strength of a variable's direct influence on another variable. It manifests as the standardized path coefficient between the two variables after controlling for all other variables in the model, reflecting the "net effect" of the independent variable on the dependent variable. For example, after controlling for variables such as the average annual summer temperature and river network density, the path coefficient from "population density" to "level of cholera prevalence" represents the direct effect of population density on cholera outbreaks. Indirect effects refer to the cumulative impact of a variable on the dependent variable through one or more mediating variables, calculated as the sum of the products of the standardized coefficients across all mediating paths. For instance, "road network density" may influence cholera epidemic levels by first increasing "population density" (path: road network density → population density), which then affects cholera epidemic levels (path: population density → cholera epidemic levels). The combined effect generated by this transmission path constitutes the indirect effect of road network density. The total effect is the sum of the direct effect and all potential indirect effects, representing the overall strength of the independent variable's influence on the dependent variable through all defined pathways in the model. This satisfies the quantitative relationship: Total Effect = Direct Effect + Indirect Effect.*

***The formation mechanism of the cholera epidemics in Hubei Province***

*……*

***Formation Mechanisms of Endogenous Variables in Cholera Epidemics***

*Table 4 presents the influence of different geographical environmental factors on the transmission and spread of cholera at the county level. The total effects represent the direct impacts of the endogenous variables (i.e., population density, river network density, and annual mean summer temperature) on cholera epidemics, with the strength and direction of these effects determined by the estimated standardized regression coefficients. All three endogenous variables exerted positive direct effects on cholera incidence, indicating that increases in population density, river network density, and annual mean summer temperature all contributed to the diffusion and transmission of cholera.*

*……*

***Formation Mechanisms of Exogenous Variables in Cholera Epidemics***

*Exogenous variables can not only act directly on the cholera epidemics, but also indirectly by acting on endogenous variables. The overall effect of exogenous variables can be categorized into direct and indirect effects. The direct effect is the estimated value of the standardized regression coefficient of the environmental factor on the level of cholera epidemics, while the indirect effect is the product of the direct effect of the environmental factor on the source of infection, the route of transmission, and the susceptible population, respectively, and the direct effect of the three endogenous variables on the level of cholera epidemics.*

1. **Fig. 2 Theoretical framework diagram of the formation mechanism of cholera epidemics:** Since multiple factors exist within the "Human Factor" "Natural Factor" and "Disaster Factor" categories in the framework diagram, and their expected impact directions vary, it is not feasible to directly add "+" or "-" symbols to the arrows. Therefore, we have added superscript "+" or "-" symbols after each factor to indicate its expected impact direction. The specific modifications are as follows (P 7):

***Fig. 2 Theoretical framework diagram of the formation mechanism of cholera epidemics***

***Note: The symbols "+" (positive) and "-" (negative) in the figure indicate the expected direction of influence for each factor.***

4. **Fig. 11 Comparison of time series between cholera and influencing factors in Hubei Province from 1949 to 2020:** We have moved it to the section "Summary of cholera epidemics formation mechanisms in Hubei Province" on page 27-28, to facilitate summarizing the formation mechanisms of cholera epidemics.

5. As recommended, all figures and tables included in the article were tested with the PLOS NAAS tool (https://ngplosjournals.pagemajik.ai/artanalysis) to verify that they fully meet the submission specifications of the journal. Meanwhile, the direct access links to the base maps have been supplemented in the figure captions of Figure 1, Figure 8 and Figure 9 for clarity and traceability.

**Comment 1-8.**

**Specific Data and Methodology Questions**

1. **Vaccination Data**: You state vaccination explains the 1955-1979 cholera-free period. Do you have any quantitative data on vaccination coverage rates from Chinese public health records, even if incomplete? If so, why wasn't it included in the SEM?

2. **1954 vs. 1998 Floods**: You mention both floods—1954 (no cholera) and 1998 (204 cases). This is a natural experiment. Could you quantify flood severity (e.g., flooded area, population affected) and include it in your analysis? This would strengthen your flood disaster findings.

**3. Time-Series Nature of Data**: Your raw data table shows annual counts from 1949-2020. How exactly did you aggregate this for the SEM? Did you:

o Use all 72 years × 103 counties = 7,416 observations?

o Time-average each county's 72 years into one observation (n=103)?

o Use only epidemic years?

This needs explicit clarification in the Methods.

**4. Separate Pre-1980 and Post-1980 Analyses**: Given the dramatic differences in surveillance, ElTor introduction, and socioeconomic development, have you considered running separate SEMs for 1949-1979 vs. 1980-2020? This might reveal different mechanisms in different eras.

**5. Model Validation:** Have you validated your SEM by testing whether it can predict the 2000-2020 period using only pre-2000 data? This would strengthen confidence in the identified mechanisms.

**Response 1-8.**

Thank you for your valuable feedback on the article "Specific Data and Methods". Below are specific revisions and responses to each point:

1.We appreciate your attention to the role of vaccination in cholera control. The previous statement regarding vaccination's role in cholera prevention was indeed incomplete: while vaccination was one factor contributing to the effective control of cholera outbreaks between 1955 and 1979, it was not the primary factor. To address this, we have revised the relevant statements in the Discussion section (P 28-29) of the revised manuscript. We now explicitly define vaccination as a supplementary measure in cholera control and shift the focus of our argument to the core control measures more aligned with historical facts—namely, the public health intervention system centered on population management, quarantine in affected areas, and case isolation. The specific revisions are as follows:

***Discussion***

***Research Extension and Comparison***

*Temporal Variations in Cholera Epidemics in Hubei Province. First, the 25-year cholera silent period (1955-1979) identified in this study constitutes one of the salient features of its spatiotemporal distribution pattern. This phenomenon was a phased outcome of the combined effects of robust administrative interventions and a relatively closed social system under specific historical conditions [52]. In the early years of the People's Republic of China, the implementation of strategies such as preventive vaccination, transportation quarantine, and rapid blockade of epidemic foci, coupled with the household registration system and the prevailing international environment at that time, restricted population mobility. This significantly reduced the risk of pathogen introduction and dissemination, thereby sustaining the "zero-reporting" status throughout this period. However, the resurgence of cholera in Hubei Province since the 1980s was primarily driven by a shift in the underlying epidemiological mechanisms, which can be attributed to four key factors. First, the exponential increase in population mobility brought about by the reform and opening-up policy created favorable conditions for the introduction of Vibrio cholerae El Tor biotype, a novel pathogenic strain. Second, the enhanced environmental survivability and higher infectivity of this strain enabled it to easily breach the prior static defense system based on isolation control and vaccination [53]. Third, in the 1980s, drinking water safety issues remained unresolved in many regions, and a considerable number of cholera cases were caused by water source contamination. Fourth, some areas exhibited strategic flaws in epidemic prevention and control practices, including overly extensive blockade of epidemic sites, irrational coverage of chemoprophylaxis, and a prominent tendency of "prioritizing treatment over prevention". Moreover, the emphasis on economic development at the expense of social benefits led to delays in epidemic reporting and prevention, triggering the spread of cholera outbreaks. Thus, the cholera-free period of 1955-1979 represented a fragile epidemic prevention equilibrium achieved through administrative means within a closed system. In contrast, the epidemic trends since the 1980s have revealed that, against an open socioeconomic backdrop, the mechanism of cholera emergence has been restructured into a complex interplay of population mobility amid globalization, the evolutionary adaptation of pathogens themselves, and regional local environmental factors. At the same time, considering the evolution of China's national cholera control strategy, its approach had historically relied on robust public health measures, including the "three controls" (water, sewage, and food management), a sensitive surveillance system, and timely outbreak containment [42]. Although cholera vaccines were used as an adjunct in certain regions during the mid-20th century, they never became a cornerstone of the national prevention and control framework. This further suggested that the fluctuations in incidence observed in Hubei—such as the resurgence in the 1980s—were more attributable to shifts in environmental and socioeconomic drivers, as well as potential adjustments in the intensity of core sanitation measures.*

Furthermore, existing historical records on vaccination primarily consist of qualitative descriptions in administrative reports (e.g., "launching large-scale preventive vaccination campaigns") or scattered vaccination coverage percentages. Such data not only lack continuous, comparable time series at the county-level administrative unit, but their spatial resolution (typically provincial or regional) and temporal frequency (non-consecutive annual records) also fail to align with the county-level, time-aggregated panel data structure constructed for this study. Consequently, they were excluded from the structural equation model's analytical framework.

2. Thank you for your insightful suggestions. The two major flood events in 1954 and 1998 you mentioned, along with the starkly different characteristics of cholera outbreak responses in the two locations, undoubtedly provide an excellent natural experiment for examining the complex coupling relationship between disasters and epidemics. However, it should be clarified that the core objective of this study lies in identifying relatively stable drivers shaping the long-term spatial risk patterns of cholera. Due to constraints such as paper length, single extreme hydrological events are not the primary focus of this research. We deeply regret the inability to conduct an in-depth quantitative analysis of this exemplary comparative case within this study. Nevertheless, we believe this represents a research direction of significant academic value and practical application potential. In future research, we will further explore threshold conditions and moderating factors within the "disaster-epidemic" chain of effects, aiming to provide more robust scientific support for precision prevention and control strategies grounded in risk assessment.

3. Regarding data aggregation in SEM. The current SEM employs time-averaged cross-sectional data. This study's structural equation model aims to identify steady-state drivers shaping cholera's long-term spatial patterns. To this end, we aggregated data across the temporal dimension. Specifically: For each variable across all counties, we calculated its representative value over the entire study period from 1949 to 2020. For cholera case counts, we used cumulative total cases; for continuous variables like temperature and precipitation, we employed multi-year averages; for flood and drought disasters, we utilized cumulative occurrence years. Consequently, the final data structure input to the model is a cross-sectional dataset comprising 103 observations, each representing the aggregated characteristics of a county over 72 years. We have provided detailed explanations in the Data Source section and added a new table specifying each variable's definition, unit, time range, processing method, and role within the structural equation model. The specific modifications are as follows(P 7-10):

*(2) Influencing factor data.……The structural equation model in this study aims to identify the steady-state drivers shaping the long-term spatial patterns of cholera. To this end, we aggregated the data along the temporal dimension. Specifically: for each variable across all counties, we calculated a representative value spanning the entire study period from 1949 to 2020. For cholera case counts, we used cumulative total cases; for continuous variables like temperature and precipitation, we employed multi-year averages; for flood and drought disasters, we utilized cumulative occurrence years. Thus, the final data structure input to the model is a cross-sectional dataset comprising 103 observations, each representing a county's aggregated characteristics over 72 years. All variables involved in this study—including their definitions, units, time ranges, processing methods, and roles within the structural equation model—are summarized in Table 1.*

*Table 1 Definitions and Processing Instructions for All Variables in the Model*

| ***Variable Name*** | ***Variable Definition and Description*** | ***Unit*** | ***Time Coverage and Processing Method*** | ***Role in the Model*** |
| --- | --- | --- | --- | --- |
| ***Cholera Epidemic*** | *The cumulative total of confirmed cholera cases reported by each county between 1949 and 2020 reflects the historical cumulative intensity of cholera epidemics in the region and serves as a core observational indicator for research.* | *case* | *Complete study period from 1949 to 2020, utilizing cumulative confirmed case counts from county health statistics reports and disease surveillance systems, with duplicate reports excluded.* | *Dependent variable, representing the outcome variable of a cholera epidemic* |
| ***Population Density*** | *The density of regional population distribution reflects the concentration of susceptible individuals and serves as a key demographic indicator influencing the transmission efficiency of infectious diseases.* | *Persons/km²* | *Based on the data from 7 census years (1953, 1964, 1982, 1990, 2000, 2010, and 2020), the county-scale data were supplemented by the spatial interpolation method, and the average value during the study period was calculated.* | *Endogenous variable, serving as a proxy variable for the size of susceptible population* |
| ***Annual mean summer temperature*** | *The average temperature from June to August (summer) each year reflects the cumulative summer heat level of a region and affects the survival and reproduction efficiency of Vibrio cholerae.* | *°C* | *For the complete study period from 1949 to 2020, county-scale data were extracted through spatial overlay analysis based on monthly temperature raster data, and the annual average values during the study period were calculated.* | *Endogenous variable, serving as a proxy variable for the living environment of Vibrio cholerae (infectious source)* |
| ***River Network Density*** | *The ratio of the total length of rivers to the regional area within a county reflects the degree of water system development and the spatial characteristics of water body coverage, and serves as a key environmental carrier indicator for cholera water-borne transmission.* | *km・km⁻²* | *As static geographic data, river elements within county boundaries were extracted based on the 1:100,000 water system vector data from Open Street Map (OSM), and the total river length per unit area was calculated.* | *Endogenous variable, serving as a proxy variable for the water-borne transmission route of cholera* |
| ***Annual Mean GDP*** | *The average annual gross domestic product (GDP) within a county, which reflects the regional economic development level, the capacity for public health resource allocation, and the potential for medical prevention and control investment.* | *ten thousand yuan* | *Actual measurement data from 1949 to 2020 (sourced from statistical yearbooks), with annual averages calculated for the study period.* | *Exogenous variable, serving as a core indicator representing the level of social and economic development* |
| ***Night Lights*** | *The annual average nighttime light radiation intensity within a county reflects the regional urbanization level, intensity of human activities, and the level of infrastructure improvement* | *nW/(cm²·sr)* | *From 1985 to 2020, based on the DMSP-OLS (Defense Meteorological Satellite Program-Operational Linescan System) nighttime light dataset, the county-level average values were calculated after conducting data consistency correction.* | *Exogenous variable, serving as a proxy variable for urbanization and human activity intensity* |
| ***Road Density*** | *The ratio of the total length of highways/roads within a county to the regional area reflects traffic accessibility, the convenience of personnel mobility, and the efficiency of public health emergency response.* | *km・km⁻²* | *Covering 6 time nodes (1962, 1980, 1990, 2000, 2010, and 2020), county-level highway networks were extracted based on transportation vector map data, and the average value of total highway length per unit area was calculated* | *Exogenous variable, an indicator representing traffic connectivity and emergency response capacity* |
| ***Mean Elevation*** | *The average value of topographic altitude within a county reflects the characteristics of regional terrain relief, affects climatic conditions and human settlement patterns, and indirectly acts on cholera transmission* | *m* | *As static geographic data, it was obtained through county boundary clipping and mean value statistics based on the 30m-resolution SRTM DEM* | *Exogenous variable, a basic indicator representing natural geographical characteristics* |
| ***Annual Mean Precipitation*** | *The annual average precipitation within a county reflects the characteristics of regional aridity and humidity, affects the stability of the aquatic environment and the transmission vector conditions of Vibrio cholerae.* | *mm* | *For the complete study period from 1949 to 2020, county-scale data were extracted via spatial overlay analysis based on the 0.5°×0.5° monthly precipitation raster data, and the annual average values over the study period were calculated.* | *Exogenous variable, a key indicator representing natural climatic characteristics* |
| ***Annual Mean Sunshine*** | *The annual average value of sunshine duration in a county is able to reflect the degree of abundance of solar radiation resources in the region* | *hour* | *From 1960 to 2020, based on the monthly sunshine raster data generated from meteorological station observation data and spatial interpolation, the annual average values at the county scale over the study period were calculated* | *Exogenous variable, an indicator representing climatic conditions* |
| ***Years of Flooding*** | *The cumulative frequency of flood disasters within counties from 1949 to 2000 reflects the intensity of water inundation events caused by heavy rainfall, snowmelt and other factors, and is prone to triggering waterborne transmission of cholera* | *Frequency* | *From 1949 to 2000, flood disaster events with clear time records within counties were counted based on historical disaster records* | *Exogenous variable, an indicator representing disaster disturbance factors* |
| ***Years of Drought*** | *The cumulative frequency of drought disasters in counties from 1949 to 2000 reflects the intensity of water shortage events caused by long-term insufficient precipitation, and affects sanitary conditions and disease prevention and control* | *Frequency* | *From 1949 to 2000, drought disaster events with clear time records within counties were counted based on historical disaster records* | *Exogenous variable, an indicator representing disaster disturbance factors* |

*Note: This model adopts a time aggregation strategy, where all variables are processed into a representative value for each county-level unit over the study period (1949-2020), forming a cross-sectional data structure for structural equation model (SEM) analysis*

4. We appreciate your insightful suggestion to conduct a comparative analysis across different time periods. We fully acknowledge that China underwent changes in its socioeconomic structure, disease surveillance systems, and cholera pathogen spectrum around 1980, which theoretically could have led to differing formation mechanisms. We have seriously considered performing a SEM analysis across these time periods. However, after thorough evaluation, we found this approach faces fundamental challenges in data availability, rendering it unfeasible for this study: First, key variables are systematically missing for the early period. Multiple core explanatory variables relied upon in this study are either unavailable or severely incomplete at the county level prior to 1980. For instance, nighttime light data begins only in 1985, and road network data only provides a single data point from 1962. The absence of these variables would result in a severely under-specified SEM model for the 1949–1979 period, making meaningful comparisons with later models impossible and rendering the results unreliable due to missing critical variables. Second, splitting the 103 county-level observations by time period drastically reduces the sample size for each timeframe (especially earlier periods). For complex SEMs involving multiple latent variables and paths, excessively small samples lead to highly unstable parameter estimates, inflated standard errors, and statistically ineffective model results. Therefore, the explanatory power of the SEM model we currently present should be more accurately understood as revealing which geographic and socioeconomic background factors robustly explain the long-term spatial patterns of cholera risk under long-term average conditions spanning different developmental stages. Should future breakthroughs be achieved in historical data reconstruction, conducting comparative studies across different time periods would represent a highly valuable avenue for further research.

5. We appreciate the reviewers' important questions regarding model validation. We understand that validating models through temporal out-of-sample prediction is the standard for assessing predictive performance. However, it should be clarified that the SEM constructed in this study was primarily designed for "explanation" rather than "prediction". Its design objectives and data structure render it unsuitable for such validation. Therefore, conducting rigorous temporal out-of-sample prediction is not applicable within the framework of this study. The model's "validation" is primarily demonstrated through goodness-of-fit indices (Table 3) and the statistical significance/reasonableness of path coefficients.

**Comment 1-9.**

**Data Availability**

You state "All data underlying the findings described in this manuscript are publicly available without restriction." However:

• Given the extensive historical data compilation from local chronicles (Appendix 1 lists dozens of sources), it would be helpful to deposit your compiled dataset in a repository (e.g., Figshare, Dryad, or Zenodo) with a DOI

• Specify exact URLs for the environmental data sources (you cite the National Tibetan Plateau Data Center multiple times—include direct links)

• For reproducibility, consider providing your SEM code (SPSS/Amos syntax) as supplementary material

**Response 1-9.**

Thank you for your valuable suggestions on enhancing data transparency. We fully agree with your perspective and have implemented the following key steps accordingly:

1. We have systematically organized and uploaded the core datasets used in this study—including the consolidated county-level historical cholera data (1949–2020) and all environmental and socioeconomic factor data analyzed in the paper—to the internationally recognized Zenodo data repository. This dataset has been assigned a Digital Object Identifier (DOI): 10.5281/zenodo.18008359 (https://zenodo.org/records/18008359). This permanent link ensures long-term accessibility and citability of the data.

2. Detailed references are provided for all environmental data. The sources for precipitation, temperature, and sunshine duration—which you mentioned as being obtained from the National Qinghai-Tibet Plateau Science Data Center—are also cited in the references ([28,29,30]).

3. Regarding reproducibility, the MK test code used in this paper is included in the supplementary materials uploaded with the manuscript. For other analytical methods: wavelet analysis was performed using MATLAB software, while the structural equation model was completed using the Amos plugin in SPSS 27.0. Detailed operational procedures for these methods are outlined in the paper's Methodology section, and the relevant analyses do not require additional supporting code.

**Reviewer 1**

**Comment 1-1.**

The manuscript is suitable for the journal's readership, as it addresses a relevant and timely topic related to the environmental and climatic determinants of cholera. The study provides valuable insights into the long-term relationship between cholera incidence and ecological, climatic, and demographic variables in Hubei Province, China, from 1949 to 2020. Using a combination of statistical and spatial correlation models, the authors effectively identify the significant factors associated with disease dynamics, including population density, temperature, precipitation, drought and flood frequency, as well as economic development. The manuscript makes a meaningful contribution to the literature on infectious disease ecology and environmental health.

One of the manuscript's main strengths lies in its use of multiple complementary methodologies, which allows for a robust and multidimensional interpretation of the data. The integration of climatological, hydrological, and socioeconomic information strengthens the analytical framework and enhances the validity of the findings. Additionally, the use of an extensive temporal dataset spanning more than seven decades provides a unique historical perspective on the evolution of cholera risk factors in the region. This longitudinal approach is particularly commendable, as it captures long-term environmental changes and their cumulative effects on disease patterns.

To further improve the clarity and contextual understanding of the manuscript, the authors are encouraged to define all acronyms upon first mention, ensuring accessibility for a broad readership that may include professionals from interdisciplinary backgrounds.

**Response 1-1.**

We sincerely thank the reviewers for their constructive and insightful comments on our manuscript. We are particularly grateful for the reviewers' recognition of this study's relevance, methodological robustness, and the value of its longitudinal data. We fully agree with your suggestion that all abbreviations should be defined upon their first mention to facilitate understanding for interdisciplinary readers. Accordingly, we have systematically reviewed and revised the entire text. Key revisions include: (1) Full names have been added for variables appearing for the first time in the "Data Sources" section(P); (2) Adding full names and brief descriptions for all statistical and spatial analysis methods (e.g., M-K test, SEM, GIS) upon their first appearance in the "Methodology" section(P 7、11-13) . Specific revisions are as follows:

***Data source***

*……*

*Gross Domestic Product (GDP) data were from China County Statistical Yearbook and Hubei Statistical Yearbook 1991*

***Methodology***

***Mann-Kendall Test (M-K Test).*** *The Mann-Kendall Test (M-K Test) is a nonparametric method widely used in climate, hydrology, and other fields to analyze trends and detect abrupt changes in time series data. Its key advantage is that the data did not need to follow a specific distribution and were less affected by outliers.*

*……*

***Geographic Information System (GIS) Spatial Analysis.*** *(1) Spatial Autocorrelation Analysis. Spatial Autocorrelation Analysis can measure the spatial distribution pattern of an outbreak based on the similarity of spatial locations and attributes. It can also detect spatial clustering and anomalies, including both global and local spatial autocorrelation.*

……

**Structural Equation Model (SEM).** Structural Equation Model (SEM) is a statistical method based on the covariance matrix of variables that analyzes the relationship between variables (including the role of exogenous variables on endogenous variables and the relationship between endogenous variables, etc.).

……

**Comment 1-2.**

Furthermore, to help readers situate the study within the broader public health context, it would be helpful to include a concise description of China's national cholera prevention program, including the year the vaccination program was introduced, the type of vaccine employed, and its estimated effectiveness. This contextual information would enable readers to understand better how policy interventions may have impacted cholera trends during the study period.

**Response 1-2.**

Thank you for your constructive suggestion regarding the addition of background information on China's national cholera prevention program. We agree that this information will help readers better understand our findings within the broader context of public health policy. Accordingly, we have added a concise explanation (P) to the Research Extension and Comparison subsection in the Discussion section of the manuscript. This supplementary note clarifies that China's cholera control relies primarily on comprehensive sanitation and surveillance measures rather than mass vaccination, as cholera vaccines were not central to the national strategy during our study period. This context helps explain why the epidemiological trends we observed in Hubei Province were more directly driven by the environmental and socioeconomic factors we analyzed. The specific revision is as follows (P 28-29):

***Discussion***

***Research Extension and Comparison***

*Temporal Variations in Cholera Epidemics in Hubei Province. First, the 25-year cholera silent period (1955-1979) identified in this study constitutes one of the salient features of its spatiotemporal distribution pattern. This phenomenon was a phased outcome of the combined effects of robust administrative interventions and a relatively closed social system under specific historical conditions [52]. In the early years of the People's Republic of China, the implementation of strategies such as preventive vaccination, transportation quarantine, and rapid blockade of epidemic foci, coupled with the household registration system and the prevailing international environment at that time, restricted population mobility. This significantly reduced the risk of pathogen introduction and dissemination, thereby sustaining the "zero-reporting" status throughout this period. However, the resurgence of cholera in Hubei Province since the 1980s was primarily driven by a shift in the underlying epidemiological mechanisms, which can be attributed to four key factors. First, the exponential increase in population mobility brought about by the reform and opening-up policy created favorable conditions for the introduction of Vibrio cholerae El Tor biotype, a novel pathogenic strain. Second, the enhanced environmental survivability and higher infectivity of this strain enabled it to easily breach the prior static defense system based on isolation control and vaccination [53]. Third, in the 1980s, drinking water safety issues remained unresolved in many regions, and a considerable number of cholera cases were caused by water source contamination. Fourth, some areas exhibited strategic flaws in epidemic prevention and control practices, including overly extensive blockade of epidemic sites, irrational coverage of chemoprophylaxis, and a prominent tendency of "prioritizing treatment over prevention". Moreover, the emphasis on economic development at the expense of social benefits led to delays in epidemic reporting and prevention, triggering the spread of cholera outbreaks. Thus, the cholera-free period of 1955-1979 represented a fragile epidemic prevention equilibrium achieved through administrative means within a closed system. In contrast, the epidemic trends since the 1980s have revealed that, against an open socioeconomic backdrop, the mechanism of cholera emergence has been restructured into a complex interplay of population mobility amid globalization, the evolutionary adaptation of pathogens themselves, and regional local environmental factors. At the same time, considering the evolution of China's national cholera control strategy, its approach had historically relied on robust public health measures, including the "three controls" (water, sewage, and food management), a sensitive surveillance system, and timely outbreak containment [42]. Although cholera vaccines were used as an adjunct in certain regions during the mid-20th century, they never became a cornerstone of the national prevention and control framework. This further suggested that the fluctuations in incidence observed in Hubei—such as the resurgence in the 1980s—were more attributable to shifts in environmental and socioeconomic drivers, as well as potential adjustments in the intensity of core sanitation measures.*

**Comment 1-3.**

Regarding the limitations, although the authors acknowledge potential data constraints, it is recommended that the discussion be expanded to include underreporting, particularly in the early decades of the study period, and that the potential impact of this limitation on the results be addressed. The years following 1949 were characterized by limited surveillance capacity and inconsistent reporting practices, which could have led to an underestimation of case numbers and, consequently, affected the identification of long-term trends. A deeper reflection on this issue would enhance the transparency and critical depth of the discussion section.

**Response 1-3.**

Thank you for your insightful comments. We fully agree that a thorough analysis of data limitations, particularly underreporting due to insufficient surveillance capacity during the early stages of the study and its potential impact on trend identification, is crucial for enhancing research transparency and critical depth. Accordingly, we have systematically reviewed and revised the entire manuscript. Key modifications include: (1) Adding a new subsection titled "Data Quality and Limitations" under "Data Sources" (P), which discusses the temporal variations in cholera surveillance capabilities across different periods, their potential impact on data quality, and limitations regarding cholera risk factors. (2) In the "Research Extension and Comparison" subsection of the "Discussion" section (P), we have elaborated on the formation of the 25-year cholera-free period (1955-1979) and the reasons for the resurgence of outbreaks after 1980. (3) In the "Research Limitations" subsection of the "Discussion" section (P 10-11、28-29、34-35), we further explored the impact of limitations in cholera data quality on the spatiotemporal distribution and its formation mechanisms. Specific revisions are as follows:

***Data source***

*……*

*（3）Data Quality and Limitations. Although the cholera epidemic data in this study have undergone multi-source verification, they still have certain limitations due to the constraints of technical conditions and objective environments in specific historical periods. The core influencing factors of data quality stem from the developmental differences in cholera surveillance systems across different eras, with specific manifestations as follows: Before the 1970s, the surveillance system was initially established and relied on manual case reporting. The data underreporting rate was high, and only the rough scope of the epidemic could be grasped, resulting in prominent limitations. During the 1980s-1990s, the surveillance network was gradually taking shape, and the sentinel system for intestinal clinics was fully popularized, which significantly improved the standardization and completeness of surveillance data. However, problems such as insufficient accuracy of strain typing still existed. Since the 21st century, the introduction of molecular biology technologies such as PCR and whole-genome sequencing has achieved a leap-forward improvement in the accuracy of surveillance data, providing core support for strain tracing and transmission chain analysis. With the real-time uploading and integration of data, the underreporting rate has dropped to below 1%. Nevertheless, insufficient grassroots testing capacity in remote areas remains a shortcoming.In general, China’s cholera surveillance system has shown a staged evolution characteristic of "from passive response to active intelligent early warning", and the data quality has undergone significant changes with socio-economic development and technological innovation [42].*

*The selection of influencing factors in this study was guided primarily by the theoretical framework, while also taking into account the principle of data availability. Constrained by the consistency and continuity of historical data series at the county scale, there were significant discrepancies in the completeness of datasets for various influencing factors: Among them, temperature, precipitation, and economic data could be continuously obtained at the county scale throughout the entire study period, with high data completeness; In contrast, only cross-sectional data at breakpoint of each era were available for population density and road network density datasets, making it impossible to form continuous time series. Data on drought and flood disaster sequences were missing after 2000; sunshine duration data were absent for the period 1949-1959; and nighttime light data were not available from 1949 to 1984; given the strong stability of topography and river-lake distribution, elevation and river network data from 2020 were used as substitutes. In addition, some factors that have been proven to affect cholera epidemics could not be quantitatively incorporated into the model due to data limitations. For example, vaccination coverage, water supply and sanitation facility conditions, etc., could not be directly included in statistical analyses due to the lack of standardized quantitative data at the county scale.*

***Discussion***

***Research Extension and Comparison***

*Temporal Variations in Cholera Epidemics in Hubei Province. First, the 25-year cholera silent period (1955-1979) identified in this study constitutes one of the salient features of its spatiotemporal distribution pattern. This phenomenon was a phased outcome of the combined effects of robust administrative interventions and a relatively closed social system under specific historical conditions [52]. In the early years of the People's Republic of China, the implementation of strategies such as preventive vaccination, transportation quarantine, and rapid blockade of epidemic foci, coupled with the household registration system and the prevailing international environment at that time, restricted population mobility. This significantly reduced the risk of pathogen introduction and dissemination, thereby sustaining the "zero-reporting" status throughout this period. However, the resurgence of cholera in Hubei Province since the 1980s was primarily driven by a shift in the underlying epidemiological mechanisms, which can be attributed to four key factors. First, the exponential increase in population mobility brought about by the reform and opening-up policy created favorable conditions for the introduction of Vibrio cholerae El Tor biotype, a novel pathogenic strain. Second, the enhanced environmental survivability and higher infectivity of this strain enabled it to easily breach the prior static defense system based on isolation control and vaccination [53]. Third, in the 1980s, drinking water safety issues remained unresolved in many regions, and a considerable number of cholera cases were caused by water source contamination. Fourth, some areas exhibited strategic flaws in epidemic prevention and control practices, including overly extensive blockade of epidemic sites, irrational coverage of chemoprophylaxis, and a prominent tendency of "prioritizing treatment over prevention". Moreover, the emphasis on economic development at the expense of social benefits led to delays in epidemic reporting and prevention, triggering the spread of cholera outbreaks. Thus, the cholera-free period of 1955-1979 represented a fragile epidemic prevention equilibrium achieved through administrative means within a closed system. In contrast, the epidemic trends since the 1980s have revealed that, against an open socioeconomic backdrop, the mechanism of cholera emergence has been restructured into a complex interplay of population mobility amid globalization, the evolutionary adaptation of pathogens themselves, and regional local environmental factors. At the same time, considering the evolution of China's national cholera control strategy, its approach had historically relied on robust public health measures, including the "three controls" (water, sewage, and food management), a sensitive surveillance system, and timely outbreak containment [42]. Although cholera vaccines were used as an adjunct in certain regions during the mid-20th century, they never became a cornerstone of the national prevention and control framework. This further suggested that the fluctuations in incidence observed in Hubei—such as the resurgence in the 1980s—were more attributable to shifts in environmental and socioeconomic drivers, as well as potential adjustments in the intensity of core sanitation measures.*

*……*

***Research Limitations***

*(1) Cholera Data. The cholera epidemic data employed in this study were derived from systematic collection of historical documents. However, their quality was inevitably constrained by the varying levels of development of surveillance systems across different periods and regions—a common challenge faced in all historical disease research. First, diachronic changes in surveillance sensitivity might affect trend interpretation. In the early period (1949–1979), when the surveillance system was in its initial stage of establishment, potential case underreporting suggested that the "low-incidence periods" or "disease-free periods" documented in this study might, to a certain extent, reflect the limited surveillance capacity of that era rather than the absolute eradication of the disease. In contrast, the improvement of the surveillance network and the standardization of reporting systems since the 1980s and 1990s might have led to a "statistical increase" in recorded case numbers, which was superimposed on the actual epidemic fluctuations. Second, the uneven spatial coverage of surveillance might have amplified the observed east-west disparities. In the early stages, medical resources, surveillance awareness, and reporting systems in Wuhan (the provincial capital) and counties in the eastern plains were superior to those in the remote mountainous areas of western Hubei Province. Therefore, the sparse or even zero recorded cases in western Hubei Province were likely the combined result of "genuine low disease risk" and "insufficient sensitivity of the surveillance system". This could have led our model to overestimate the protective effects of natural geographical factors (e.g., high altitude, low river network density) and to reinforce the "ldense in the east and sparse in the west" spatial pattern. While reflecting real disease risks, the observed pattern might also have, to some degree, exaggerated the actual disparities between eastern and western regions. Thus, simply classifying western Hubei Province as a "low-risk area" might lack precision; a more rigorous characterization was that it was a "low-reporting area confirmed based on existing data", and its true epidemic situation awaits further investigation. Despite these limitations, we had strived to construct the most complete historical epidemic sequence through systematic cross-validation of multi-source local chronicles and yearbooks. Future research can further refine the reconstruction of historical epidemic dynamics by mining finer-grained historical archives.*

**Comment 1-4.**

In the recommendations, most of the proposed measures focus appropriately on environmental control strategies, such as integrating water monitoring into surveillance systems. However, it would strengthen the public health relevance of the conclusions to explicitly include the recommendation of ensuring access to safe and potable water for the population. Given that contaminated water remains one of the principal transmission routes for Vibrio cholerae, such an addition would align the recommendations with the global health framework for cholera elimination promoted by the World Health Organization.

**Response 1-4.**

Thank you for your important supplementary suggestions. We fully agree that explicitly incorporating recommendations to ensure safe drinking water is crucial for enhancing the practical value of public health research. To this end, we have supplemented the "Recommendations" section of the paper with a dedicated recommendation specifically addressing "ensuring drinking water safety"(P 33-34). The specific revisions are as follows:

***Policy Recommendations***

*To effectively prevent and respond to future cholera outbreaks, based on research into the spatio-temporal patterns and formation mechanisms of cholera epidemics in Hubei Province, the following Hubei-specific prevention and control recommendations are proposed. These offer particular reference value for regions with dense river and lake networks:……(4) Joint prevention and control at transportation hubs: Relying on hubs such as Wuhan Tianhe Airport and Hankou Railway Station, establish a mechanism of "symptom monitoring for personnel entering Hubei Province and rapid testing of water samples in luggage". Implement "on-arrival testing" for flights and trains from cholera-affected areas, and establish a "real-time case information sharing platform" with surrounding provinces to block the transmission chain of imported epidemics. (5) Ecological management and control of rivers and lakes: Establish "cholera prevention and control ecological corridors" in key river sections such as the Wuhan section of the Yangtze River and the Xiangyang section of the Han River. It is strictly prohibited to discharge domestic sewage into rivers during the flood season. Complete the construction of "regular monitoring points for Vibrio cholerae" at all drinking water sources at or above the county level by May each year, and implement closed-loop management of "supply suspension—disinfection—re-inspection" for water bodies that exceed standards. Furthermore, "ecological corridor" development should be deeply integrated with the national "Yangtze River Protection" strategy and local "Beautiful Rivers and Lakes" initiatives, translating pathogen control targets into specific ecological design parameters for wetland restoration and shoreline remediation projects. Monitoring station construction can be incorporated into routine fiscal safeguards for drinking water sanitation. (6) Establish a Safe Drinking Water Assurance System. As the most fundamental public health measure for cholera prevention and control, ensuring drinking water safety requires systematically enhancing water safety assurance capabilities beyond existing environmental monitoring. This includes improving water treatment and disinfection capacity to guarantee the stable effectiveness of chlorination and other processes. During early warning periods, temporarily increase residual chlorine levels in treated water before distribution and intensify testing of terminal pipelines in older urban areas. Addressing shortcomings in rural decentralized water supply, advance projects to consolidate and enhance drinking water safety. Promote small-scale water purification facilities and household disinfection equipment, while strengthening training for water management personnel and users on water quality assessment and equipment maintenance.*

**Reviewer 3**

**Comment 1-1.**

The objectives of the study become apparent but are not as clearly articulated as they might be. The study design is mostly appropriate, with limitations likely beyond the control of the authors (due to requirement for historical data). I am not well placed to comment on all the statistical approaches used, but I did wonder if some approaches were being used because they could be (M-K test, Wavelet analysis), rather than because they needed to be. Examples (references) of where these analyses have been used for similar purposes would help justify the analytic approach used in this study. Variables investigated seemed a bit haphazard, though all could potentially have some impact on V. cholerae. More justification for some variables (e.g. nighttime lighting) would help justify the study

**Response 1-1.**

We sincerely appreciate your insightful and constructive review comments on this paper. Your suggestions regarding the articulation of research objectives, rationale for methodological choices, and systematic explanation of variables are crucial for enhancing the scientific rigor of this work. We have systematically revised and refined the manuscript based on each of your recommendations.

1. Clarifying Research Objectives: At the conclusion of the "Introduction" section, we explicitly formulated three progressive core research questions, thereby enhancing the purposefulness and logical coherence of the analytical framework throughout the paper. The specific revisions are as follows (P 4):

***Introduction***

*……*

*However, existing studies still have some limitations: In terms of spatiotemporal patterns, analyses have mainly focused on the spatiotemporal patterns of cholera in some regions[18-22]. There is still a lack of geographical studies with long-term and high spatiotemporal resolution on regional cholera epidemics. In terms of formation mechanisms, there is still a lack of comprehensive quantitative studies on the formation mechanisms of regional cholera epidemics, and there is also insufficient identification of major influencing factors and analysis of their action mechanisms [18, 23]. In terms of cholera research in Hubei Province, there is a lack of specialized studies on cholera epidemics in this region, which is densely covered with rivers and lakes and has a severe history of epidemics. Therefore, to address these research gaps, this study aims to systematically answer three core scientific questions: (1) The long-term trends, cyclical fluctuations, and seasonal characteristics of cholera epidemics in Hubei Province from 1949 to 2020; (2) Its overall distribution patterns and spatial distribution changes; (3) The formation mechanisms of these spatiotemporal patterns shaped by multidimensional factors including natural conditions, disaster events, and socioeconomic factors. To this end, this study integrates time-series analysis, spatial statistics, and structural equation modeling to elucidate the complex mechanisms underlying regional cholera epidemics. It aims to provide scientific insights for cholera prevention and control in river- and lake-rich regions, particularly for other areas globally facing similar environmental conditions (e.g., monsoon climate, dense river networks) and socioeconomic pressures (e.g., rapid urbanization, high population mobility).*

2. Strengthening Methodological Justification: For methods such as the Mann-Kendall test and wavelet analysis, we supplemented their classical application cases and references in the field of spatiotemporal analysis of infectious diseases, demonstrating their applicability and advantages in analyzing long-term sequence data within this study. The specific revisions are as follows (P 11-12):

***Mann-Kendall Test (M-K Test).*** *The Mann-Kendall Test (M-K Test) is a nonparametric method widely used in climate, hydrology, and other fields to analyze trends and detect abrupt changes in time series data. Due to its robustness to non-specific data distributions and insensitivity to missing values[43], the M-K test is widely applied in trend analysis and mutation detection for long-term infectious disease sequences[27, 44, 45]. Spanning 72 years, this study's early data might exhibit discontinuities and recording biases; the M-K test reliably identified overall trends and statistical significance in cholera incidence rates.*

***Wavelet Analysis.*** *Wavelet Analysis is a new branch of mathematics formed in the late 1980s, which is widely used in many fields such as signal processing because of its good time-frequency localization analysis capability [46]. The basic idea of wavelet analysis is to use a cluster of wavelet function systems to represent or approximate a signal or function. The wavelet function is the key to wavelet analysis, and there are many wavelet functions that could be selected. This paper selectes the Morlet wavelet function, primarily based on two considerations. First, the Morlet wavelet exhibits good localization properties in both the time and frequency domains, allowing for an optimal balance between temporal resolution and frequency resolution. This makes it particularly suitable for analyzing non-stationary time series such as cholera case counts[27, 44, 45]. Second, its oscillatory waveform better matches potential quasi-periodic fluctuation signals within the series, enabling effective extraction of latent periodic components. Compared to the Daubechies wavelet, which is more adept at capturing abrupt changes, or the Mexican hat wavelet (Ricker wavelet), which has relatively weaker frequency-domain resolution, the Morlet wavelet is better aligned with the analytical objectives of this study. In terms of parameter settings, continuous wavelet transform was employed, with the scale range corresponding to periods from 2 to 35 years, to detect potential fluctuations from short-term to long-term cycles. The scales were densely sampled using a logarithmic equal-interval approach across 64 scale levels to ensure the continuity of the periodogram, thereby enabling precise identification of the dominant cycles in the series. By computing the real part of the wavelet coefficients, the oscillation phase and intensity of the series across different time scales can be analyzed; meanwhile, calculating the wavelet variance allows for the determination of the dominant periodic scales. In this paper, Matlab software’s wavelet analysis tool was used to calculate the real part of wavelet coefficients and wavelet variance of the number of cholera cases in Hubei Province, and Surfer software was used to draw the contour map of the real part of wavelet coefficients.*

3. We have added a new table titled "Definitions and Processing Notes for All Variables in the Model" to the "Data Sources" section. This table includes the definition, unit, time range, and function of each factor in the structural equation model, aiding in a clearer understanding of each variable's role within the model. Specific modification requirements are as follows (P 7-10):

*(2) Influencing factor data.……The structural equation model in this study aims to identify the steady-state drivers shaping the long-term spatial patterns of cholera. To this end, we aggregated the data along the temporal dimension. Specifically: for each variable across all counties, we calculated a representative value spanning the entire study period from 1949 to 2020. For cholera case counts, we used cumulative total cases; for continuous variables like temperature and precipitation, we employed multi-year averages; for flood and drought disasters, we utilized cumulative occurrence years. Thus, the final data structure input to the model is a cross-sectional dataset comprising 103 observations, each representing a county's aggregated characteristics over 72 years. All variables involved in this study—including their definitions, units, time ranges, processing methods, and roles within the structural equation model—are summarized in Table 1.*

*Table 1 Definitions and Processing Instructions for All Variables in the Model*

| ***Variable Name*** | ***Variable Definition and Description*** | ***Unit*** | ***Time Coverage and Processing Method*** | ***Role in the Model*** |
| --- | --- | --- | --- | --- |
| ***Cholera Epidemic*** | *The cumulative total of confirmed cholera cases reported by each county between 1949 and 2020 reflects the historical cumulative intensity of cholera epidemics in the region and serves as a core observational indicator for research.* | *cases* | *Complete study period from 1949 to 2020, utilizing cumulative confirmed case counts from county health statistics reports and disease surveillance systems, with duplicate reports excluded.* | *Dependent variable, representing the outcome variable of a cholera epidemic* |
| ***Population Density*** | *The density of regional population distribution reflects the concentration of susceptible individuals and serves as a key demographic indicator influencing the transmission efficiency of infectious diseases.* | *Persons/km²* | *Based on the data from 7 census years (1953, 1964, 1982, 1990, 2000, 2010, and 2020), the county-scale data were supplemented by the spatial interpolation method, and the average value during the study period was calculated.* | *Endogenous variable, serving as a proxy variable for the size of susceptible population* |
| ***Annual mean summer temperature*** | *The average temperature from June to August (summer) each year reflects the cumulative summer heat level of a region and affects the survival and reproduction efficiency of Vibrio cholerae.* | *°C* | *For the complete study period from 1949 to 2020, county-scale data were extracted through spatial overlay analysis based on monthly temperature raster data, and the annual average values during the study period were calculated.* | *Endogenous variable, serving as a proxy variable for the living environment of Vibrio cholerae (infectious source)* |
| ***River Network Density*** | *The ratio of the total length of rivers to the regional area within a county reflects the degree of water system development and the spatial characteristics of water body coverage, and serves as a key environmental carrier indicator for cholera water-borne transmission.* | *km・km⁻²* | *As static geographic data, river elements within county boundaries were extracted based on the 1:100,000 water system vector data from Open Street Map (OSM), and the total river length per unit area was calculated.* | *Endogenous variable, serving as a proxy variable for the water-borne transmission route of cholera* |
| ***Annual Mean GDP*** | *The average annual gross domestic product (GDP) within a county, which reflects the regional economic development level, the capacity for public health resource allocation, and the potential for medical prevention and control investment.* | *ten thousand yuan* | *Actual measurement data from 1949 to 2020 (sourced from statistical yearbooks), with annual averages calculated for the study period.* | *Exogenous variable, serving as a core indicator representing the level of social and economic development* |
| ***Night Lights*** | *The annual average nighttime light radiation intensity within a county reflects the regional urbanization level, intensity of human activities, and the level of infrastructure improvement* | *nW/(cm²·sr)* | *From 1985 to 2020, based on the DMSP-OLS (Defense Meteorological Satellite Program-Operational Linescan System) nighttime light dataset, the county-level average values were calculated after conducting data consistency correction.* | *Exogenous variable, serving as a proxy variable for urbanization and human activity intensity* |
| ***Road Density*** | *The ratio of the total length of highways/roads within a county to the regional area reflects traffic accessibility, the convenience of personnel mobility, and the efficiency of public health emergency response.* | *km・km⁻²* | *Covering 6 time nodes (1962, 1980, 1990, 2000, 2010, and 2020), county-level highway networks were extracted based on transportation vector map data, and the average value of total highway length per unit area was calculated* | *Exogenous variable, an indicator representing traffic connectivity and emergency response capacity* |
| ***Mean Elevation*** | *The average value of topographic altitude within a county reflects the characteristics of regional terrain relief, affects climatic conditions and human settlement patterns, and indirectly acts on cholera transmission* | *m* | *As static geographic data, it was obtained through county boundary clipping and mean value statistics based on the 30m-resolution SRTM DEM* | *Exogenous variable, a basic indicator representing natural geographical characteristics* |
| ***Annual Mean Precipitation*** | *The annual average precipitation within a county reflects the characteristics of regional aridity and humidity, affects the stability of the aquatic environment and the transmission vector conditions of Vibrio cholerae.* | *mm* | *For the complete study period from 1949 to 2020, county-scale data were extracted via spatial overlay analysis based on the 0.5°×0.5° monthly precipitation raster data, and the annual average values over the study period were calculated.* | *Exogenous variable, a key indicator representing natural climatic characteristics* |
| ***Annual Mean Sunshine*** | *The annual average value of sunshine duration in a county is able to reflect the degree of abundance of solar radiation resources in the region* | *hour* | *From 1960 to 2020, based on the monthly sunshine raster data generated from meteorological station observation data and spatial interpolation, the annual average values at the county scale over the study period were calculated* | *Exogenous variable, an indicator representing climatic conditions* |
| ***Years of Flooding*** | *The cumulative frequency of flood disasters within counties from 1949 to 2000 reflects the intensity of water inundation events caused by heavy rainfall, snowmelt and other factors, and is prone to triggering waterborne transmission of cholera* | *Frequency* | *From 1949 to 2000, flood disaster events with clear time records within counties were counted based on historical disaster records* | *Exogenous variable, an indicator representing disaster disturbance factors* |
| ***Years of Drought*** | *The cumulative frequency of drought disasters in counties from 1949 to 2000 reflects the intensity of water shortage events caused by long-term insufficient precipitation, and affects sanitary conditions and disease prevention and control* | *Frequency* | *From 1949 to 2000, drought disaster events with clear time records within counties were counted based on historical disaster records* | *Exogenous variable, an indicator representing disaster disturbance factors* |

*Note: This model adopts a time aggregation strategy, where all variables are processed into a representative value for each county-level unit over the study period (1949-2020), forming a cross-sectional data structure for structural equation model (SEM) analysis*

**Comment 1-2.**

Data presentation is very good, and results are clearly presented in the form of figures and tables. My question, as stated elsewhere, is are all analyses justified?

**Response 1-2.**

Thank you for acknowledging the data presentation approach in this study and for your continued interest in the analytical methods. As mentioned above, we have strengthened the methodological justification. For techniques such as the Mann-Kendall test and wavelet analysis, we have supplemented their classical application cases and references in the field of spatiotemporal analysis of infectious diseases, demonstrating their applicability and advantages in analyzing the long-term time series data within this study.

**Comment 1-3.**

Both the Results and Discussion and Conclusion sections need to be edited. Conclusions more typically come after the Discussion. The conclusions shouldn't be a numbered list. The Discussion is also essentially a numbered list.

**Response 1-3.**

Thank you for your attention to the article format and for your valuable suggestions. We have revised the article format, placing the conclusion after the discussion section and not presenting it as a numbered list. The specific changes are as follows(P 28-36):

***Discussion and Conclusions***

***Discussion***

*……*

***Conclusions***

*In terms of time variation. The cholera epidemics in Hubei Province from 1949 to 2020 showed a pattern of "concentrated high-incidence periods, significant periodic fluctuations, and prominent seasonal characteristics". Its long-term downward trend and multi-scale fluctuation cycles essentially reflect the long-term interplay between climate stability, social prevention and control capabilities, and pathogen variation. The high incidence in summer and autumn highlights the key driving role of the warm and humid summer environment in the reproduction of Vibrio cholerae.*

*In terms of spatial distribution. The pattern of "dense in eastern Hubei Provinceand sparse in western Hubei Province", along with the characteristics of "agglomeration along rivers and lakes, and more severe in plains than mountains", reveals the decisive role of geographical environment and human activities in the spatial differentiation of the epidemic. The "westward and southward shift" of the epidemic's core reflects the reshaping of the epidemic's spread path by regional development disparities and the allocation of prevention and control resources.*

*In terms of formation mechanism. The temporal and spatial patterns of cholera epidemics in Hubei Province are the result of the combined effects of natural factors, disaster factors and human factors at multiple scales；Population density and summer temperatures were the direct factors driving the spread of cholera epidemics, while river networks formed the basic environmental background that facilitated transmission. Natural environmental factors such as drought and flood disasters, altitude, and precipitation, as well as human environmental factors including road networks and economic conditions, could not only directly drive the spread of epidemics but also regulate epidemics development through indirect pathways. Its formation mechanism provides a typical case for understanding the "environment-society-disease" interaction and offers a theoretical basis for formulating differentiated "regional and time-phased" prevention and control strategies.*

**Comment 1-4.**

Cholera outbreaks have sporadically occurred in Hubei, and it is pleasing to see data analysis of those outbreaks. Such analyses can lead to more targeted interventions, as is the stated intention of the manuscript. I feel that some of the discussion points, while important in the control of cholera, were not directly relevant to the analyses conducted. I think the manuscript could be made shorter and more focused, and would be better for it. The authors must clearly state what the aims were, and then focus on those outcomes in the discussion.

As stated in the early section of the Discussion "The cholera outbreaks in Hubei during the 1980s were closely linked to the introduction of this new pathogen". Indeed, it is the presence of the pathogen that is likely the most important factor in whether a cholera outbreak occurs. I acknowledge that other factors are important (presence alone does not result in a cholera outbreak), so this study does add to the body of knowledge around cholera transmission in China, and indeed globally to some extent.

While the standard of writing is generally good, it is a long manuscript and should be edited to make it shorter and improve clarity. In doing so, please ensure you adhere to conventions of scientific writing, not the least italics for genus and species names!

Some minor points to improve clarity:

State approximate year range of Jiaqing-Daoguang period of the Qing Dynasty for benefit of international readers

"especially after the 21st century" we are currently in the 21 century, so change to "after 2000" or "during 21st century"

"The climate is mild and humid, with abundant rainfall, no severe cold in winter, and no cool weather in summer".

Add specific details such as annual rainfall and min and max average daily temperatures.

"Third, overly broad implementation of epidemic foci blockade and drug-based prevention, combined with insufficient human and material resources, resulted in ineffective intervention measures." Please clarify this sentence.

Please provide more information about past vaccination programs in China.

"For instance, the prolonged absence of cholera outbreaks in Hubei Province after 1949 was closely associated with strict preventive vaccination measures." To the best of my knowledge, oral cholera vaccines became available in the 1980s. So what were the strict preventative vaccination measures after 1949? If vaccination has been in place for a long time (maybe 30 – 40 years) perhaps state when it was first used in this region.

It is also implied that vaccination was being used in 1954? If so, as it a locally developed vaccine? Some more details are required. Reference 50 is presumably not widely available, and appears to not be a peer-reviewed article.

**Response 1-4.**

1.Thank you for your valuable feedback. You pointed out that while some perspectives in the Discussion section are important for cholera prevention and control, they are not directly related to the analytical content of this study. We fully agree with your perspective and have revised the discussion section to strengthen its connection to the study's content. We have organized the discussion into three subsections. The first section covers research extensions and comparisons. Regarding temporal variations, it further explores the causes of the 25-year cholera-free period and the 1980 cholera resurgence, the background correlations of the 29a, 19a, and 8a cycles, and potential reasons for the high incidence of cholera during summer and autumn. Second, spatial distribution: we further explore the underlying drivers of shifts in cholera's center of gravity. Third, model construction: we further investigate the causes of numerical anomalies in certain variables. Additionally, we compare our study's conclusions with those of other research. The second subsection presents policy recommendations based on our findings. The third subsection outlines the study's limitations. Specific revisions are as follows (P 28-36):

***Discussion***

***Research Extension and Comparison***

*Temporal Variations in Cholera Epidemics in Hubei Province. First, the 25-year cholera silent period (1955-1979) identified in this study constitutes one of the salient features of its spatiotemporal distribution pattern. This phenomenon was a phased outcome of the combined effects of robust administrative interventions and a relatively closed social system under specific historical conditions [52]. In the early years of the People's Republic of China, the implementation of strategies such as preventive vaccination, transportation quarantine, and rapid blockade of epidemic foci, coupled with the household registration system and the prevailing international environment at that time, restricted population mobility. This significantly reduced the risk of pathogen introduction and dissemination, thereby sustaining the "zero-reporting" status throughout this period. However, the resurgence of cholera in Hubei Province since the 1980s was primarily driven by a shift in the underlying epidemiological mechanisms, which can be attributed to four key factors. First, the exponential increase in population mobility brought about by the reform and opening-up policy created favorable conditions for the introduction of Vibrio cholerae El Tor biotype, a novel pathogenic strain. Second, the enhanced environmental survivability and higher infectivity of this strain enabled it to easily breach the prior static defense system based on isolation control and vaccination [53]. Third, in the 1980s, drinking water safety issues remained unresolved in many regions, and a considerable number of cholera cases were caused by water source contamination. Fourth, some areas exhibited strategic flaws in epidemic prevention and control practices, including overly extensive blockade of epidemic sites, irrational coverage of chemoprophylaxis, and a prominent tendency of "prioritizing treatment over prevention". Moreover, the emphasis on economic development at the expense of social benefits led to delays in epidemic reporting and prevention, triggering the spread of cholera outbreaks. Thus, the cholera-free period of 1955-1979 represented a fragile epidemic prevention equilibrium achieved through administrative means within a closed system. In contrast, the epidemic trends since the 1980s have revealed that, against an open socioeconomic backdrop, the mechanism of cholera emergence has been restructured into a complex interplay of population mobility amid globalization, the evolutionary adaptation of pathogens themselves, and regional local environmental factors. At the same time, considering the evolution of China's national cholera control strategy, its approach had historically relied on robust public health measures, including the "three controls" (water, sewage, and food management), a sensitive surveillance system, and timely outbreak containment [42]. Although cholera vaccines were used as an adjunct in certain regions during the mid-20th century, they never became a cornerstone of the national prevention and control framework. This further suggested that the fluctuations in incidence observed in Hubei—such as the resurgence in the 1980s—were more attributable to shifts in environmental and socioeconomic drivers, as well as potential adjustments in the intensity of core sanitation measures. Next, this study found that cholera epidemics peaked between August and October, coinciding with the warm and humid summer-autumn seasons in Hubei Province. Against this seasonal high-risk background, large-scale family and social gatherings were frequent, particularly around the Mid-Autumn Festival and National Day. Existing research has demonstrated that inadequate food hygiene supervision at such collective dining events can serve as critical nodes for foodborne cholera outbreaks [52,54]. The epidemic records collected in this study also contain explicit documentation of cases linked to mass gathering [55]. Therefore, although the temporal resolution of the data precluded the statistical isolation of the specific risk associated with the holiday days themselves, the intensive dining culture during the "two festivals" likely overlapped with the inherent climatic risk period, jointly shaping the epidemic peak pattern observed from August to October. In addition, this study identified three significant cholera epidemic cycles of 29, 19, and 8 years, which may reflect the coupling of multi-scale driving processes. The 29a cycle aligned with the epidemiological cycle of "introduction-epidemic-suppression" for major Vibrio cholerae biotypes (e.g., the El Tor biotype in the 1960s) and concurrent socio-economic transformations (e.g., the Reform and Opening-up period), suggesting it was a composite biosocial cycle shaped jointly by pathogen, host, and social factors. The 19a cycle corresponded closely to the activity period of low-frequency climate modes such as the Pacific Decadal Oscillation, potentially modulating regional climatic and hydrological backgrounds and setting an environmental rhythm at the decadal scale for epidemics. The 8a cycle was associated with interannual climate variability such as the El Niño-Southern Oscillation and the triggering frequency of flood disasters, reflecting the role of extreme weather events as direct "triggers" for outbreaks. Together, these cycles constituted the comprehensive temporal characteristics of cholera epidemics under multiple mechanisms, including long-term pathogen evolution, medium-term climatic fluctuations, and short-term disaster triggers.*

*Regarding the spatial changes of cholera epidemics in Hubei Province. The shift in the epidemic's center of gravity was not merely a result of the simple accumulation of cases; instead, it reflected the transformation of core areas of population and socio-economic activities within the province. During 1949-1959, the center was located in the Xiaogan region, which corresponded to the relatively higher population density and agricultural activity levels in the eastern Jianghan Plain (e.g., Xiaogan and Huangpi). With the rise of Wuhan as a central city in China after the 1980s, its massive population size and frequent internal and external exchanges made it an indisputable hotspot for cholera outbreaks in the 1980s-1990s, leading to a southward shift of the center to urban Wuhan (e.g., Jiangxia and Honghu). From the late 1990s to the 2000s, although the number of cases in Wuhan decreased, counties along the Yangtze River, such as Jingzhou and Jiangling, emerged as new key epidemic areas due to their role as secondary population centers in the province and their traditional reliance on water bodies for rice cultivation. This collectively contributed to a further southwestward shift of the center toward the hinterland of the Jianghan Plain (e.g., Honghu). This trajectory essentially outlined how the dynamic changes in the center of socio-economic activities and population distribution in Hubei Province over the past half-century had shaped the spatial pattern of infectious disease risks.*

*Regarding the influencing factors of cholera epidemics. The results of the structural equation model (SEM) showed that nighttime light exhibited a significant positive total effect on cholera epidemic levels (1.080), yet this finding required cautious interpretation. Nighttime light is a typical high-order composite variable that is strongly correlated with both population density and GDP. It may simultaneously capture multiple dimensions of information, such as population agglomeration in urbanized areas, intensity of economic activities, density of infrastructure, and even the capacity for surveillance and reporting. The coexistence of its positive direct effect (1.139) and negative indirect effect (-0.059) suggested that there may be unfully identified collinear structures within the model, or inhibitory effects generated through other pathways (e.g., improved medical response capabilities). Therefore, nighttime light should not be simply interpreted as an independent "risk driver"; instead, it ought to be regarded as a comprehensive indicator of "risk-surveillance co-occurrence" that marked regional development levels. Furthermore, the study found that GDP and nighttime light exerted positive impacts on cholera epidemic levels, which contradicts the common understanding that "economic development improved sanitation facilities and reduced the risk of waterborne infectious diseases". Two potential reasons for this discrepancy were hypothesized: First, real risk amplification effect. Economically developed and highly urbanized regions such as Wuhan, the large population size, frequent cross-regional mobility, dense transportation networks, and overloaded sewage treatment systems collectively create a favorable environment for cholera transmission. Historically, multiple cluster outbreaks had been concentrated in such areas, indicating that socio-economic activities themselves might exert an "amplification effect" on epidemic spread. Second, surveillance and reporting bias. Economically developed regions were equipped with more robust infectious disease surveillance systems, advanced diagnostic capabilities, and stringent reporting protocols, resulting in far higher case detection and reporting rates than underdeveloped regions. The observed "high epidemic levels" might not stem entirely from differences in actual incidence; instead, they could be systematic errors caused by disparities in public health surveillance capacity—a problem that was difficult to avoid in retrospective studies utilizing passively reported data.*

*Regarding the comparison of this study’s findings with other research. Fist, the driving mechanisms of cholera in Hubei Province revealed in this study not only align with the general patterns of global cholera epidemics but also exhibit distinct characteristics due to environmental differences between inland water networks and coastal brackish water convergence zones. This study found that the average summer temperature was one of the strongest environmental factors driving cholera epidemics. This finding was highly consistent with observations from major global cholera-endemic regions. In Bangladesh, rising sea surface temperatures had been confirmed as a key driver of seasonal epidemics and interannual outbreaks; in the African Great Lakes region, temperature increases following the rainy season often coincided with epidemic outbreaks. Together, these findings corroborated the core role of temperature as a "universal switch" regulating the proliferation rate of Vibrio cholerae in aquatic environments [17, 21]. However, the uniqueness of this case lied in its purely inland freshwater environment. Unlike coastal and estuarine epidemic areas (such as the Bay of Bengal), which were strongly influenced by salinity gradients and tidal effects, the river and lake networks in Hubei Province exhibited stable and extremely low salinity. Our model indicateed that the direct impact of river network density itself was weaker, suggesting that in freshwater systems, dense water networks provided the necessary medium for transmission but are not a sufficient condition determining epidemic intensity. The spatial differentiation of epidemics was primarily determined by the degree of population aggregation and climatic suitability windows superimposed on this hydrological background. This "hydrological base + socio-climatic amplifier" mechanism provideed a theoretical framework for understanding cholera risks in other inland water-rich regions (such as large lake basins). Second, the transmission pathways of cholera were undergoing a global evolution. The increased records of outbreaks triggered by group gatherings in Hubei Province since the 1980s, as observed in this study, were not an isolated phenomenon. Instead, they reflected a global trend shifting from traditional "waterborne transmission" dominance toward an increasing importance of "foodborne transmission" [54, 56]. As urban and rural water supply and sanitation infrastructure improve, the proportion of outbreaks directly caused by drinking water contamination had declined. However, the lengthening of food supply chains and the frequent occurrence of group dining activities driven by globalization and urbanization had created new pathways for Vibrio cholerae cross-contamination through food. The case of Hubei Province demonstrated that even in inland regions, socio-economic development and lifestyle changes were reshaping the transmission dynamics of infectious diseases. This necessitated that modern cholera prevention and control must transcended a singular environmental sanitation perspective and incorporate systematic supervision of food supply chain safety and collective dining hygiene. In addtion, since 1949, the impact of disaster factors on cholera epidemics had gradually weakened. The root cause of this transformation lied in the progress China had achieved since 1949 in improving medical and health standards, sustaining economic development, and maintaining social stability, which had laid a more solid foundation for cholera prevention and control [27].*

***Policy Recommendations***

*To effectively prevent and respond to future cholera outbreaks, based on research into the spatio-temporal patterns and formation mechanisms of cholera epidemics in Hubei Province, the following Hubei-specific prevention and control recommendations are proposed. These offer particular reference value for regions with dense river and lake networks: (1) Risk management during key periods: Focus should be placed on the high-incidence summer and autumn seasons, especially August and September. Based on the main cycles of 55 years and 29 years for cholera in Hubei Province, preventive measures should be taken in advance in predicted peak years. (2) Establish a climate-adaptive early warning and response system. Given the critical driving role of climate conditions in this research model, future global warming will prolong summer heatwaves and increase the frequency of extreme precipitation events. This is highly likely to extend the "risk window" for cholera transmission and increase outbreak frequency. Therefore, the prevention and control system must be climate-adaptive. Seasonal risk outlooks based on climate forecasts could be issued each spring. During El Niño development phases, advance assessments of their potential impact on the following year's epidemic should be conducted. Flood disaster warnings should be directly linked to cholera emergency surveillance activation mechanisms, enabling enhanced monitoring of water bodies and diarrheal symptoms in key areas within 72 hours post-disaster. (3) Hierarchical spatial management and control: Level-1 risk zones (Wuhan urban area, 3 counties along the Yangtze River in Xianning): Implement sluice biosurveillance stations, install automatic sampling and testing equipment at the sluices of the Yangtze River/Han River. When vibrio levels exceed standards, trigger the closure of ecological gates to block the invasion of polluted water into urban inland lakes. To reduce implementation costs, this system can be integrated with existing water quality monitoring stations and water conservancy hubs operated by water resources departments. By sharing station locations, power supplies, and communication networks, it achieves functional convergence of hydrological and pathogen monitoring, forming a "multi-functional integrated monitoring platform". Level-2 risk zones (other counties and cities in the Jianghan Plain): Incorporate water vibrio concentration into the assessment system of the river chief system; suspend fishery and tourism projects in river sections that fail to meet standards. This strategy can be directly embedded into China's existing "River and Lake Chief System" governance framework. Implementation requires only adding assessment indicators without creating new administrative bodies, offering significant advantages of low administrative resistance and high synergistic benefits. Level-3 risk zones (western Hubei Province mountainous areas): Set up quarantine stations at inter-county transportation hubs (such as the Three Gorges Dam in Yichang) to conduct random inspections on freight vehicles from historical epidemic areas. Leveraging existing joint enforcement mechanisms between transportation and agriculture departments, deploy additional health quarantine personnel during high-risk summer and autumn periods. This creates a flexible prevention model combining routine patrols with peak-period monitoring, enabling low-cost, high-efficiency interception of imported risks. (4) Joint prevention and control at transportation hubs: Relying on hubs such as Wuhan Tianhe Airport and Hankou Railway Station, establish a mechanism of "symptom monitoring for personnel entering Hubei Province and rapid testing of water samples in luggage". Implement "on-arrival testing" for flights and trains from cholera-affected areas, and establish a "real-time case information sharing platform" with surrounding provinces to block the transmission chain of imported epidemics. (5) Ecological management and control of rivers and lakes: Establish "cholera prevention and control ecological corridors" in key river sections such as the Wuhan section of the Yangtze River and the Xiangyang section of the Han River. It is strictly prohibited to discharge domestic sewage into rivers during the flood season. Complete the construction of "regular monitoring points for Vibrio cholerae" at all drinking water sources at or above the county level by May each year, and implement closed-loop management of "supply suspension—disinfection—re-inspection" for water bodies that exceed standards. Furthermore, "ecological corridor" development should be deeply integrated with the national "Yangtze River Protection" strategy and local "Beautiful Rivers and Lakes" initiatives, translating pathogen control targets into specific ecological design parameters for wetland restoration and shoreline remediation projects. Monitoring station construction can be incorporated into routine fiscal safeguards for drinking water sanitation. (6) Establish a Safe Drinking Water Assurance System. As the most fundamental public health measure for cholera prevention and control, ensuring drinking water safety requires systematically enhancing water safety assurance capabilities beyond existing environmental monitoring. This includes improving water treatment and disinfection capacity to guarantee the stable effectiveness of chlorination and other processes. During early warning periods, temporarily increase residual chlorine levels in treated water before distribution and intensify testing of terminal pipelines in older urban areas. Addressing shortcomings in rural decentralized water supply, advance projects to consolidate and enhance drinking water safety. Promote small-scale water purification facilities and household disinfection equipment, while strengthening training for water management personnel and users on water quality assessment and equipment maintenance. (7) Hubei's unique breakfast culture of eating breakfast on the street carries the risk of contamination from blanched raw foods. It is recommended to promote the "Double-Non" certification (non-river-water dishwashing, non-raw/cold ingredients) in scenic spots such as Hubu Lane in Wuhan, and incorporate it into the evaluation criteria for Hubei's (Jingchu) dietary intangible cultural heritage. This certification system can be embedded within the market supervision department's existing "quantitative grading for food safety" dynamic evaluation, serving as a bonus point or specialty certification for "transparent kitchens". Market reputation incentives can encourage voluntary participation by businesses, avoiding additional mandatory administrative burdens.*

***Research Limitations***

*(1) Cholera Data. The cholera epidemic data employed in this study were derived from systematic collection of historical documents. However, their quality was inevitably constrained by the varying levels of development of surveillance systems across different periods and regions—a common challenge faced in all historical disease research. First, diachronic changes in surveillance sensitivity might affect trend interpretation. In the early period (1949–1979), when the surveillance system was in its initial stage of establishment, potential case underreporting suggested that the "low-incidence periods" or "disease-free periods" documented in this study might, to a certain extent, reflect the limited surveillance capacity of that era rather than the absolute eradication of the disease. In contrast, the improvement of the surveillance network and the standardization of reporting systems since the 1980s and 1990s might have led to a "statistical increase" in recorded case numbers, which was superimposed on the actual epidemic fluctuations. Second, the uneven spatial coverage of surveillance might have amplified the observed east-west disparities. In the early stages, medical resources, surveillance awareness, and reporting systems in Wuhan (the provincial capital) and counties in the eastern plains were superior to those in the remote mountainous areas of western Hubei Province. Therefore, the sparse or even zero recorded cases in western Hubei Province were likely the combined result of "genuine low disease risk" and "insufficient sensitivity of the surveillance system". This could have led our model to overestimate the protective effects of natural geographical factors (e.g., high altitude, low river network density) and to reinforce the "ldense in the east and sparse in the west" spatial pattern. While reflecting real disease risks, the observed pattern might also have, to some degree, exaggerated the actual disparities between eastern and western regions. Thus, simply classifying western Hubei Province as a "low-risk area" might lack precision; a more rigorous characterization was that it was a "low-reporting area confirmed based on existing data", and its true epidemic situation awaits further investigation. Despite these limitations, we had strived to construct the most complete historical epidemic sequence through systematic cross-validation of multi-source local chronicles and yearbooks. Future research can further refine the reconstruction of historical epidemic dynamics by mining finer-grained historical archives.*

*(2) Environmental factor data. The consideration of factors affecting cholera epidemics is not fully comprehensive. For instance, the medical and health care level in Hubei Province (such as county-level diagnosis and treatment capabilities, infectious disease prevention and control systems) is one of the variables affecting the spread and control of epidemics. However, before 2000, the statistics of county-level health conditions in Hubei Province (such as the coverage rate of rural sanitation facilities) and medical standards (such as specialized diagnosis and treatment capabilities for intestinal infectious diseases) had not formed a standardized system, and there was a lack of quantitative data available for long-time-series and county-scale analysis, so they were not included in this quantitative model. In addition, water pH value, as a key environmental factor affecting the survival and reproduction of Vibrio cholerae, was also not included in the analysis because there is almost no data on pH monitoring of county-level rivers and lakes before 2000. At the same time, due to historical constraints, solar radiation data from Hubei Province's early period (1949-1982) were also excluded from the quantitative analysis of cholera outbreak mechanisms. To address these limitations, future research could supplement historical data on sanitation conditions, medical standards, water pH levels, and meteorological conditions through systematic review of local chronicles' sections on "sanitation" and "epidemic prevention" , retrospective analysis of historical archives from environmental monitoring agencies (e.g., water quality records from hydrological stations), and enhanced mining and interpolation of historical meteorological data. Building upon this foundation, more comprehensive multi-factor quantitative analyses could be conducted to deepen understanding of the driving mechanisms behind cholera epidemics.*

*(3) Goodness-of-fit of the Structural Equation Model. In this study, the sample size for the structural equation model is 103 county-level units. While this meets the basic requirements for model fitting, it remains relatively small compared to general recommendations (typically n > 200). This may, to some extent, limit the stability and generalizability of the model parameters. Future research that can obtain longer time series or higher spatial resolution data would help further enhance the robustness and explanatory power of the model.*

2.Thank you for your valuable feedback. We have proofread and revised the entire article.

First, we have italicized bacterial species names such as "*El Tor biotype of Vibrio cholerae*" and "*O139 cholera*" throughout the text.

Second, we have specified the approximate timeframe of the Jiaqing-Daoguang period of the Qing Dynasty. The specific revisions are as follows (P 2):

*In terms of spatiotemporal patterns, cholera was likely introduced into China by sea around the Jiaqing-Daoguang period (circa 1796-1850) of the Qing Dynasty.*

Third, we revised "*especially after the 21st century*" to "*especially since the year 2000*".

Fourth, specific data on Hubei's climate characteristics were added to the Study area section (P 4-5):

*The climate is mild and humid, with mild winters and cool summers. The annual average temperature ranges between 15°C and 17°C. January (winter) averages 3-4°C, while July (summer) averages 27-29°C. Precipitation is abundant, with annual rainfall totaling 800-1600 millimeters, 40-50% of which falls during the summer months (June-August), characteristic of a typical subtropical monsoon climate.*

Fifth, the discussion section has been restructured to re-examine the causes of cholera resurgence after 1980, with specific revisions as follows (P 28-29):

***Discussion***

***Research Extension and Comparison***

*Temporal Variations in Cholera Epidemics in Hubei Province. First, the 25-year cholera silent period (1955-1979) identified in this study constitutes one of the salient features of its spatiotemporal distribution pattern. This phenomenon was a phased outcome of the combined effects of robust administrative interventions and a relatively closed social system under specific historical conditions [52]. In the early years of the People’s Republic of China, the implementation of strategies such as preventive vaccination, transportation quarantine, and rapid blockade of epidemic foci, coupled with the household registration system and the prevailing international environment at that time, restricted population mobility. This significantly reduced the risk of pathogen introduction and dissemination, thereby sustaining the "zero-reporting" status throughout this period. However, the resurgence of cholera in Hubei Province since the 1980s was primarily driven by a shift in the underlying epidemiological mechanisms, which can be attributed to four key factors. First, the exponential increase in population mobility brought about by the reform and opening-up policy created favorable conditions for the introduction of Vibrio cholerae El Tor biotype, a novel pathogenic strain. Second, the enhanced environmental survivability and higher infectivity of this strain enabled it to easily breach the prior static defense system based on isolation control and vaccination [53]. Third, in the 1980s, drinking water safety issues remained unresolved in many regions, and a considerable number of cholera cases were caused by water source contamination. Fourth, some areas exhibited strategic flaws in epidemic prevention and control practices, including overly extensive blockade of epidemic sites, irrational coverage of chemoprophylaxis, and a prominent tendency of "prioritizing treatment over prevention". Moreover, the emphasis on economic development at the expense of social benefits led to delays in epidemic reporting and prevention, triggering the spread of cholera outbreaks. Thus, the cholera-free period of 1955-1979 represented a fragile epidemic prevention equilibrium achieved through administrative means within a closed system. In contrast, the epidemic trends since the 1980s have revealed that, against an open socioeconomic backdrop, the mechanism of cholera emergence has been restructured into a complex interplay of population mobility amid globalization, the evolutionary adaptation of pathogens themselves, and regional local environmental factors. At the same time, considering the evolution of China's national cholera control strategy, its approach has historically relied on robust public health measures, including the "three controls" (water, sewage, and food management), a sensitive surveillance system, and timely outbreak containment [42]. Although cholera vaccines were used as an adjunct in certain regions during the mid-20th century, they never became a cornerstone of the national prevention and control framework. This further suggests that the fluctuations in incidence observed in Hubei—such as the resurgence in the 1980s—are more attributable to shifts in environmental and socioeconomic drivers, as well as potential adjustments in the intensity of core sanitation measures.*

3.Thank you for your attention to the role of vaccination in cholera prevention and control. The previous statement in this article regarding vaccination's role in cholera control was indeed incomplete: while this measure was one factor contributing to the effective containment of cholera outbreaks between 1955 and 1979, it was not the primary factor. To address this, we have revised the relevant statements in the discussion section (P 28-29) of the updated version. Vaccination is now explicitly defined as a supplementary measure in cholera control, while the focus shifts to the core control measures more aligned with historical facts—namely, the public health intervention system centered on population management, quarantine in affected areas, and case isolation. And we have removed the potentially inappropriate reference (Ref. 50) and incorporated a more authoritative historical source (Ref. 52). The specific revisions are as follows:

***Discussion***

***Research Extension and Comparison***

*Temporal Variations in Cholera Epidemics in Hubei Province. First, the 25-year cholera silent period (1955-1979) identified in this study constitutes one of the salient features of its spatiotemporal distribution pattern. This phenomenon was a phased outcome of the combined effects of robust administrative interventions and a relatively closed social system under specific historical conditions [52]. In the early years of the People's Republic of China, the implementation of strategies such as preventive vaccination, transportation quarantine, and rapid blockade of epidemic foci, coupled with the household registration system and the prevailing international environment at that time, restricted population mobility. This significantly reduced the risk of pathogen introduction and dissemination, thereby sustaining the "zero-reporting" status throughout this period. However, the resurgence of cholera in Hubei Province since the 1980s was primarily driven by a shift in the underlying epidemiological mechanisms, which can be attributed to four key factors. First, the exponential increase in population mobility brought about by the reform and opening-up policy created favorable conditions for the introduction of Vibrio cholerae El Tor biotype, a novel pathogenic strain. Second, the enhanced environmental survivability and higher infectivity of this strain enabled it to easily breach the prior static defense system based on isolation control and vaccination [53]. Third, in the 1980s, drinking water safety issues remained unresolved in many regions, and a considerable number of cholera cases were caused by water source contamination. Fourth, some areas exhibited strategic flaws in epidemic prevention and control practices, including overly extensive blockade of epidemic sites, irrational coverage of chemoprophylaxis, and a prominent tendency of "prioritizing treatment over prevention". Moreover, the emphasis on economic development at the expense of social benefits led to delays in epidemic reporting and prevention, triggering the spread of cholera outbreaks. Thus, the cholera-free period of 1955-1979 represented a fragile epidemic prevention equilibrium achieved through administrative means within a closed system. In contrast, the epidemic trends since the 1980s have revealed that, against an open socioeconomic backdrop, the mechanism of cholera emergence has been restructured into a complex interplay of population mobility amid globalization, the evolutionary adaptation of pathogens themselves, and regional local environmental factors. At the same time, considering the evolution of China's national cholera control strategy, its approach had historically relied on robust public health measures, including the "three controls" (water, sewage, and food management), a sensitive surveillance system, and timely outbreak containment [42]. Although cholera vaccines were used as an adjunct in certain regions during the mid-20th century, they never became a cornerstone of the national prevention and control framework. This further suggested that the fluctuations in incidence observed in Hubei—such as the resurgence in the 1980s—were more attributable to shifts in environmental and socioeconomic drivers, as well as potential adjustments in the intensity of core sanitation measures.*
