# Supplementary material for: The spatio-temporal patterns and formation mechanisms of cholera epidemics in Hubei Province, China from 1949 to 2020
Source: PLoS Negl Trop Dis. 2026 Mar 18;20(3):e0014125. doi: 10.1371/journal.pntd.0014125 (PMC13020998; doi:10.1371/journal.pntd.0014125)
Supplement: Attachment — Submitted filename: Response_to_Reviewers_auresp_2.docx [file pntd.0014125.s010.docx]

Dear Editor-in-Chief,

Thank you sincerely for the effective efforts you have devoted to handling our manuscript, and thank the reviewers for their review of our paper and their valuable comments. In accordance with your and the reviewers' comments, we have carefully revised the manuscript. We are now submitting the following explanation of the revisions for your further guidance.

Sincerely yours,

The Authors

February 10, 2026

**The Spatio-temporal Patterns and Formation Mechanisms of Cholera Epidemics in Hubei Province** **, China from 1949 to 2020**

**Response to Reviewers**

**Major Comments**

**Comment 1-1.**

**Data Quality and Limitations — Substantially Addressed**

The authors have made excellent progress in this area.

**Strengths of the revision include:**

• The newly added Data Quality and Limitations subsection (Methods, pp. 10–11), which

clearly outlines the historical evolution of surveillance systems.

• The expanded Research Limitations section (Discussion, pp. 34–36), which critically

evaluates how surveillance gaps may influence spatial interpretations.

• The careful recharacterization of western Hubei as a “low-reporting area based on existing

data,” rather than definitively a “low-risk area,” demonstrating appropriate scientific

caution.

**Remaining minor concern:**

While surveillance limitations are now clearly acknowledged in the Methods and Discussion, the Abstract and Conclusions still present some findings in definitive terms (e.g., “71 counties and districts were cumulatively affected”). To ensure consistency with the nuanced discussion elsewhere, I suggest adding brief qualifying language such as “based on available records” in the Abstract and Conclusions.

**Response 1-1.**

Thank you for your valuable suggestions. We fully agree and have incorporated qualifying statements in the abstract (p.2) and conclusion (p.36) sections to reflect the limitations of the data and ensure consistency in our presentation. The specific revisions are as follows:

***Abstract***

*……*

*Results: Based on existing historical monitoring records. Temporally, high incidence occurred in the 1980s and 1990s, with summer and autumn being the dominant seasons. Three fluctuation cycles were identified: 29 years, 19 years, and 8 years. Spatially, 71 counties and districts were cumulatively affected, with Wuhan and Xianning serving as the primary hotspots.*

*……*

***Author summary***

*Cholera remains a major threat to public health, especially in regions with dense water networks. As a “province of a thousand lakes” in China, Hubei Province has a long history of cholera epidemics, but its long-term spatiotemporal patterns and driving forces were not fully understood. We studied cholera cases in Hubei Province from 1949 to 2020, combining historical records and environmental data with analytical tools like spatial analysis and statistical modeling. Based on existing historical monitoring records, we found cholera was most severe in the 1980s–1990s, peaked in summer and autumn, and had three cycles (29, 19, 8 years). Spatially, it clustered in eastern Hubei Province along rivers/lakes, with its center shifting southwest over time.*

*……*

***Conclusions***

*……*

*In terms of spatial distribution. Based on existing data, the cholera epidemics in Hubei Province exhibits a pattern of “dense in eastern Hubei Provinceand sparse in western Hubei Province”, along with the characteristics of “agglomeration along rivers and lakes, and more severe in plains than mountains”, reveals the decisive role of geographical environment and human activities in the spatial differentiation of the epidemic. The “westward and southward shift” of the epidemic's core reflects the reshaping of the epidemic's spread path by regional development disparities and the allocation of prevention and control resources.*

**Comment 1-2.**

**Structural Equation Model (SEM) — Well Addressed with One Remaining Clarification**

The revisions to the SEM framework are substantial and largely resolve my previous concerns.

**Variable selection and conceptual framework:**

• The new Table 1 (pp. 7–10) is an excellent addition and provides comprehensive documentation of all variables.

• The rationale for using annual mean summer temperature is now clearly explained and scientifically sound.

• The absence of certain variables (e.g., vaccination, pH, sanitation) is explicitly acknowledged and appropriately contextualized.

**Interpretation improvements:**

• Reframing river network density as a “geographical background condition” rather than a primary driver is appropriate given its weak coefficient (0.013).

• The reinterpretation of nighttime lights as a composite indicator reflecting both risk and surveillance capacity demonstrates a sophisticated understanding of potential biases.

• The discussion of the apparent GDP/nighttime lights paradox now appropriately considers both real risk amplification and reporting bias.

**Technical clarifications:**

• The use of time-averaged, cross-sectional data (n = 103) is now clearly stated.

• The small sample size limitation is acknowledged transparently (p. 35).

**One remaining issue requiring clarification:**

The manuscript states that data were “aggregated along the temporal dimension,” with cholera burden represented as cumulative case counts (p. 7). However, for time-varying exogenous variables such as GDP and nighttime lights, multi-year averages were used across the entire 1949–2020 period. This raises a concern about temporal alignment, given that cholera cases were heavily concentrated during the 1980s–1990s.

Specifically, averaging socioeconomic indicators across 72 years may obscure the actual conditions prevailing during peak incidence periods. For example, high GDP values in 2020 may dilute much lower GDP levels during the 1980s, when most cases occurred.

**Suggestion:**

Please add a brief explanation (2–3 sentences) in the Methods justifying why time-averaging is appropriate in this context (e.g., identifying long-term structural predispositions rather than shortterm triggers), or explicitly acknowledge this temporal mismatch as a limitation.

**Response 1-2.**

We appreciate the reviewers' valuable suggestions. We have supplemented the following explanation (p.14) in the “Structural Equation Modeling” subsection of the “Methods” section to justify the rationale for time-averaging:

***Structural Equation Model (SEM).***

*……*

*The total effect is the sum of the direct effect and all potential indirect effects, representing the overall strength of the independent variable's influence on the dependent variable through all defined pathways in the model. This satisfies the quantitative relationship: Total Effect = Direct Effect + Indirect Effect. This study employs a time-averaging approach to construct a cross-sectional dataset, aiming to identify the steady-state drivers shaping the long-term spatial patterns of cholera rather than short-term volatility triggers. By focusing on spatial variation, it aligns with the application premise of structural equation modeling in identifying long-term drivers.*

**Minor Comments**

**Comment 1-1.**

**Introduction — Improved**

• Removal of the term “Tiger Lera” improves clarity.

• The expanded rationale for focusing on Hubei Province is much stronger.

• The inclusion of three explicit research questions provides helpful structure.

**Minor suggestion:**

When introducing “Class A statutory infectious diseases,” consider briefly defining this term for international readers (e.g., “the highest threat category in China’s infectious disease classification system”)

**Response 1-1.**

Thank you for your valuable suggestions. We have added a brief definition in the introduction section (p.3) to enhance understanding for international readers. The revised sentence reads:

***Introduction***

*Cholera is one of only two Class A statutory infectious diseases in China (the highest hazard level in China's infectious disease classification system), a virulent intestinal infectious disease caused by the Vibrio cholerae[1].*

**Comment 1-2.**

**Results — Significantly Improved**

**Figures:**

• The redesigned Figure 3 with inset panels is particularly effective.

• Improved font sizes, color consistency, and labeling across figures greatly enhance readability.

**Tables:**

• Table 2 is cleaner and easier to interpret.

• Table 4 benefits from bolded significant values and clearer effect decomposition explanations.

**Minor issue:**

In Table 4, please clarify whether reported p-values are one-tailed or two-tailed (likely two-tailed).

**Response 1-2.**

We appreciate the reviewer for pointing out this omission. We have updated the note below Table 4 to explicitly state that all significance tests were two-tailed (p.25). The revised note reads:

*Note: Values are standardized regression coefficient estimates; *** indicates that the coefficient is significant at the 0.001 level, ** indicates that the coefficient is significant at the 0.01 level, and * indicates that the coefficient is significant at the 0.05 level. All significance tests are two-tailed.*

**Comment 1-3.**

**Discussion — Significantly Strengthened**

The restructured Discussion is much clearer, particularly with the addition of distinct sections on global comparison, policy implications, and limitations.

• The comparison with Bangladesh and African Great Lakes regions appropriately situates the findings globally.

• The expanded discussion on climate change and early warning systems strengthens future relevance.

• Emphasizing water, sewage, and food management over vaccination for the 1955–1979 control period better reflects historical evidence.

**One remaining concern:**

Reference 52 (local chronicles) is a valuable historical source, but for a key claim regarding national cholera control strategy, I recommend supplementing it with a peer-reviewed publication on China’s cholera control history to strengthen credibility for international readers.

**Response 1-3.**

Thank you for your constructive feedback. To enhance the credibility and international relevance of our discussion on historical cholera control strategies in China, we have supplemented the original local chronicles with a peer-reviewed review article. We have added the relevant citation to the Discussion section (p.29):

***Discussion***

***Research Extension and Comparison***

*Temporal Variations in Cholera Epidemics in Hubei Province. First, the 25-year cholera silent period (1955-1979) identified in this study constitutes one of the salient features of its spatiotemporal distribution pattern. This phenomenon was a phased outcome of the combined effects of robust administrative interventions and a relatively closed social system under specific historical conditions [42, 52].*

*“42. Pang B, Kan B. Cholera Prevention and Control in New China. Disease Surveillance, 2021, 36(9): 869-872. ”*

**Comment 1-4.**

**Writing and Language — Improved with Minor Corrections Needed**

Only a few minor language issues remain:

1. Abstract (p. 2): “has led” → “have led”

2. Introduction (p. 3): Consider briefly explaining why the El Tor biotype mattered (e.g., environmental resilience).

3. Methods (p. 10): Clarify whether the <1% underreporting rate refers to urban areas or a national average.

4. Discussion (p. 28): Adjust tense consistency (“has historically relied”).

**Response 1-4.**

We appreciate the reviewers' meticulous proofreading. We have made the following minor revisions throughout the manuscript to address these issues:

1. Abstract section (p.2): Changed “*has led*” to “*have led*”.

2. Introduction section (p.3): Briefly outlined the significance of Vibrio cholerae serovar El Tor:

***Introduction***

*……However, there were still three epidemics peaks in the 1960s, 1980s, and 1990s, predominantly caused by the El Tor biotype of Vibrio cholerae (a subtype of serogroup O1, known for its strong environmental survival and transmission potential).*

3. Methods section (P10): Clarified that the <1% underreporting rate refers to the national average:

*(3) Data Quality and Limitations.*

*……With the real-time uploading and integration of data, the underreporting rate at the national level has dropped to below 1%.*

4. Discussion section (P29): Adjusted tense for consistency: Changed “have historically relied” to “has historically relied”.

**Reviewer #1**

**Comment 1-1.**

The authors have made substantial improvements in response to the reviewers' comments. However, several issues still require clarification.

The explanation provided for river network density is appreciated. The authors state that, compared with the other two endogenous variables (population density and annual mean summer temperature), the effect value of river network density was relatively low. However, it would be essential to explicitly clarify that this variable was not statistically significant in the model.

There are some inconsistencies between the text and Table 4. For example, the reported mean elevation corresponds to the direct effect; the value for annual mean sunshine in the table is 0.110 (not 0.092); and the text describes the direct effect rather than the indirect effect. These discrepancies should be carefully reviewed and corrected to ensure consistency between the narrative and the table results.

The authors are also encouraged to use consistent terminology throughout the manuscript to refer to the same concept (for example, using either elevation or altitude, but not both interchangeably).

Finally, it appears the study's emphasis has shifted, as reflected in the conclusion that "population density and summer temperatures were the direct factors driving the spread of cholera epidemics, while river networks formed the basic environmental background that facilitated transmission." I recommend that the entire manuscript be carefully revised to ensure that the text is entirely consistent with this conclusion. The text would benefit from English-language editing.

**Response 1-1.**

We sincerely thank the reviewers for their meticulous review and further corrections to our revised manuscript. We have carefully verified and corrected inconsistencies between the text and tables, standardized terminology throughout the paper, and strengthened the logical consistency between core conclusions and the overall argument. Specific modifications are detailed below:

1. Your comments regarding the statistical significance of river network density and the shift in research focus are crucial for refining our paper. We have revised the interpretation of river network density in the subsection “Formation Mechanism of Endogenous Variables in Cholera Epidemics” within the “Results” section. We have clarified that this variable lacks statistical significance in the model, explicitly positioning it as a “background condition” rather than a statistically significant “driving factor”. We have also conducted a systematic review and revision of the entire manuscript to ensure that all discussions regarding the three core variables—“population density”, “summer temperature”, and “river network density”—from the Introduction through Methods, Results, and Discussion, strictly serve and ultimately align with the framework summarized in the Conclusions. Specific modifications include:

In the Data source section (pp.6-7), when describing the endogenous variable of river network density, its predefined role is explicitly stated: “*(2) Influencing factor data……As a classic waterborne disease, cholera is primarily transmitted through contaminated water bodies [12]. Consequently, the density and distribution patterns of river networks form the fundamental geographic vehicle and spatial context for cholera transmission. Therefore, the density of river network is selected as an important factor influencing the spread of cholera in this study*”.

In the Results, Formation Mechanisms of Endogenous Variables in Cholera Epidemics section (p.24), we have rewritten the explanatory paragraph regarding river network density. It now explicitly emphasizes that its effect is weak and statistically insignificant, thus strictly defining its role as the geographical context and potential vector for epidemic occurrence: “*(3)* As a geographical background factor for epidemic transmission, the path coefficient for river network density was 0.013 (p > 0.05), showing no statistical significance in this study. As a waterborne disease, cholera is primarily transmitted through contaminated well water and river water [12]. Theoretically, densely river-networked areas could provide Vibrio cholerae with more water environments for human contact, thereby creating potential transmission conditions. However, compared to population density and average summer temperatures, the effect size of river network density was lower and not statistically significant. This indicates that within the study model, the river network itself is not the primary statistical driver of epidemics. Instead, it should be regarded as the environmental substrate and necessary precondition for transmission to occur. Nevertheless, the spatial distribution of river network density highly correlates with epidemic clusters (Fig. 8、9), particularly revealing a spatial coupling pattern of “high river network density—high epidemic clustering” in the densely river-networked Jianghan Plain and its adjacent riverside cities and counties. This indicates that dense river networks provide the geographic foundation for cholera transmission via water sources. Without this condition, outbreaks would struggle to spread through waterways. However, relying solely on dense river networks—without sufficient population density (hosts) and suitable temperatures (promoting pathogen proliferation)—is insufficient to trigger large-scale cholera epidemics”.

2. Correction of inconsistencies between text and Table 4 data: We have conducted item-by-item verification and correction. The total effect value for annual average sunshine duration in the text (p.24) has been amended to “0.110” as shown in Table 4. Other textual descriptions regarding direct and indirect effects have been meticulously cross-checked to ensure strict correspondence with the breakdown in Table 4.

3. We have standardized all references to terrain undulation throughout the text to “elevation”, replacing occasional previous instances of “altitude” and similar terms to ensure conceptual consistency.

**Reviewer #4**

**Comment 1-1.**

1) Method Section – Data Source Paragraph:

The text currently states: “we measure the severity of cholera epidemics using the number of cholera cases as an indicator.”

I suggest replacing “severity” with “intensity”, as severity is usually measured by deaths, case fatality rate (CFR), or mortality rates, whereas the number of cases reflects intensity.

2) Wavelet Analysis Periods:

I would have conducted the wavelet analysis for the third and fourth periods (1980–2020), given the length and uncertainty surrounding the "cholera-free period". Discussing a 29-year cycle seems less meaningful. It would be more informative to focus on recent cycles when the disease is more frequent and recurrent.

3) Mann-Kendall Test:

I find the M-K test not particularly relevant, as the graph already shows that while the frequency of outbreaks is increasing, the intensity appears to be decreasing.

4) SEM Indicators – Water and Sanitation:

Water and sanitation access were not included as indicators in the structural equation model (SEM). If including them is not feasible due to data unavailability for the study period or the scale of analysis (e.g., county level), I recommend explicitly noting this as a limitation of the study.

**Response 1-1.**

We sincerely appreciate the reviewers' insightful and constructive comments. These suggestions are crucial for enhancing the accuracy and relevance of our research. We have made the following revisions based on your recommendations:

1. Terminology correction regarding “severity”:

We fully agree that “intensity” is a more precise term in this context. We have replaced “severity” with “intensity” in the “Data Sources” section of the Methods (p.5):

*(1) Cholera epidemics data. An epidemic refers to the prevalence of an infectious disease within a specific time and spatial scope. In this study, our subject is cholera, and we measure the intensity of cholera epidemics using the number of cholera cases as an indicator.*

2. We extend our gratitude to the peer reviewers for their valuable suggestions aimed at enhancing the analytical reference value of this study. Regarding the issue of focusing the research period for wavelet analysis, after careful consideration, we have decided to retain the existing research design. The core reason lies in the fact that revealing the complete and cross-stage long-term fluctuation patterns of cholera epidemics constitutes a crucial central objective of this study. Narrowing the analysis to 1980-2020 would omit critical observational data from the early evolutionary stages of the epidemic's full “dormant phase-outbreak-control” lifecycle. This omission would prevent verification of whether the identified “29-year cycle” represents a macro-rhythm permeating the entire cholera epidemic evolution process. Therefore, this study retains the full-period wavelet analysis design covering 1949-2020.

3.Regarding the Necessity of the Mann-Kendall Test (M-K Test):

We understand the reviewer's concerns. The primary purpose of the M-K test in this study is not only to validate visual trends but also to provide a nonparametric, robust statistical test to confirm the presence of statistically significant trends in long-term sequences and to assist in detecting potential inflection points. This is particularly important when handling historical data sequences that may contain missing values or exhibit non-normal distributions. We have clearly articulated the specific application purpose of the M-K test in the “Methods” section. We acknowledge that graphs offer intuitive visual trend descriptions, but the M-K test provides objective statistical evidence.

4. Regarding the exclusion of water and sanitation indicators from the structural equation model: You have identified a key limitation. We have explicitly supplemented this point in the “Research Limitations” section (p.35):

*(2) Environmental factor data. The consideration of factors affecting cholera epidemics was not fully comprehensive. First, water and sanitation facilities (such as safe drinking water coverage and hygienic toilet prevalence) are critical components in interrupting cholera transmission. However, due to the study's long time span (1949-2020) and county-level spatial scale, it was impossible to obtain continuous, standardized quantitative data covering the entire period and all regions. Consequently, these factors could not be incorporated into the model.*

**Comment 1-2.**

1) Remove the text: "After floods, a large number of deaths of humans and poultry pollute the water environment, providing conditions for the breeding of pathogenic bacteria. These contaminated water sources could reach other areas via floods, expanding the scope of transmission."

- Human deaths do not themselves “breed” bacteria. The bacteria are shed in stool of infected individuals; corpses from uninfected individuals are not a source of cholera transmission.

- Poultry are not carriers of V. cholerae, so mentioning poultry deaths as a source of cholera bacteria is misleading.

- The mechanism should focus on fecal contamination and bacterial proliferation in water, not human or animal corpses.

2) Consider documenting the rationale for increased cholera transmission following floods, based on the following:After floods, sewage and fecal matter can overflow from sanitation facilities and contaminate water sources, creating favorable conditions for the accelerated transmission of Vibrio cholerae.

3) In your text: “Droughts slowed down the transmission of Vibrio cholerae by reducing water-borne transmission routes. In general, flood disasters promoted the prevalence and transmission of cholera, while drought disasters inhibited its transmission”, I suggest adding supporting references.

I also recommend clarifying that, while drought may reduce cholera transmission in your specific context, it can increase both water-borne and interpersonal transmission. For example, a study in sub-Saharan Africa documented that cholera outbreaks were more likely under drought conditions, as limited access to safe water reduces hygiene and forces people to rely on unsafe surface water or gather closely at limited water points (https://pubmed.ncbi.nlm.nih.gov/40250229/).

While drought may lower incidence in your study area, these findings cannot be generalized to all settings. Finally, for cholera, it is more accurate to refer to incidence rather than prevalence.

**Response 1-2.**

We sincerely appreciate the reviewers' meticulous review and valuable corrections. We fully agree with your insightful comments regarding the cholera transmission mechanism and the applicability of our conclusions. We have made precise revisions to the relevant paragraphs as follows:

1. Regarding the mechanism explaining increased cholera transmission after floods. We have removed the inaccurate statement in the original text that “*After floods, a large number of deaths of humans and poultry pollute the water environment, providing conditions for the breeding of pathogenic bacteria*”. Instead, following your suggestion, we have focused on fecal contamination as the core mechanism, revising it to (pp.26-27):

*(2) Impact of disaster factors on cholera epidemics.……Following floods, damaged or overwhelmed sanitation facilities lead to the overflow of sewage and feces containing Vibrio cholerae, extensively contaminating water sources and creating ideal conditions for the pathogen's proliferation and spread in water bodies [17]. Simultaneously, densely populated post-disaster shelters with limited sanitation further increase exposure risks and interpersonal contact frequency among susceptible populations.*

We also cited supporting literature (Carrel et al., 2009 [17]) to strengthen the theoretical basis.

2. Regarding the impact of drought on cholera. First, following the conclusion that “drought suppresses its transmission”, we have supplemented the key literature supporting this view (Leckebusch & Abdussalam, 2015 [11]), specifically revising it to (P 26):

*(2) Impact of disaster factors on cholera epidemics.……Drought, conversely, limits cholera's waterborne transmission by reducing the volume of water bodies where Vibrio cholerae can survive and spread [11].*

At the same time, we have explicitly emphasized in the Abstract (p.2) and Introduction (p.4) that the conclusions of this study can provide reference for cholera prevention and control efforts in river and lake-dense regions, thereby limiting the applicability of these findings. Furthermore, we have systematically reviewed the use of the term “prevalence” throughout the manuscript and adjusted it to “incidence” or “epidemics” where necessary to ensure terminological accuracy.

**Comment 1-3.**

1) This explanation is tempting and is one of my favorite part: “Thus, the cholera-free period of 1955–1979 represented a fragile epidemic prevention equilibrium achieved through administrative means within a closed system. In contrast, the epidemic trends since the 1980s have revealed that, against an open socioeconomic backdrop, the mechanism of cholera emergence has been restructured into a complex interplay of population mobility amid globalization, the evolutionary adaptation of pathogens themselves, and regional local environmental factors.” However, I would need a bit more to be convinced. Have you looked at cholera incidence in China during this period, or in neighboring provinces, to make sure this isn’t just a reporting artifact? If cholera was largely confined to coastal areas, that could explain why it reemerged in the 1980s, when population movement increased, such as seasonal workers traveling to coastal regions. Showing some of these data would strengthen your argument.

2) In the policy section, please clarify whether “on-arrival testing” refers to stool sample testing or water sample testing. To my knowledge, there is currently no licenced RDT available to detect Vibrio cholerae in water samples. Moreover, the GTFCC surveillance guidelines recommend testing stool samples rather than water samples. For feasibility and alignment with global guidelines, consider replacing “water samples” with “stool samples” in the phrase: “symptom monitoring for personnel entering Hubei Province and rapid testing of water samples in luggage.”

Additionally, you could consider implementing “on-arrival testing” for flights and trains arriving from cholera-affected areas, and establishing a “real-time sharing platform” with neighboring provinces to interrupt the transmission chain of imported cases.

3) Consider moving policy recommendations 6 and 7 on water access and food safety higher. Since drinking water safety is the cornerstone of cholera prevention, these measures deserve greater emphasis.

**Response 1-3.**

Thank you for your valuable feedback. These suggestions are crucial for enhancing the persuasiveness of our discussion and the feasibility and rigor of our policy recommendations. We have made the following revisions accordingly:

1. Further substantiation regarding the cholera-free period from 1955 to 1979. We fully agree that this argument requires more robust evidence. We have supplemented the relevant paragraph in the “Discussion” section (p.29):

*First, the 25-year cholera silent period (1955-1979) identified in this study constitutes one of the salient features of its spatiotemporal distribution pattern. This phenomenon was a phased outcome of the combined effects of robust administrative interventions and a relatively closed social system under specific historical conditions [42, 52]. In the early years of the People's Republic of China, the implementation of strategies such as preventive vaccination, transportation quarantine, and rapid blockade of epidemic foci, coupled with the household registration system and the prevailing international environment at that time, restricted population mobility. This significantly reduced the risk of pathogen introduction and dissemination, thereby sustaining the “zero-reporting” status throughout this period. Additionally, the epidemic situation across the country and in neighboring provinces also reached historic lows during the same period [42], supporting the authenticity of the “epidemic-free period” at the regional level rather than representing a data anomaly unique to Hubei. However, the resurgence of cholera in Hubei Province since the 1980s was primarily driven by a shift in the underlying epidemiological mechanisms, which can be attributed to four key factors.*

2. Technical revision regarding “entry testing”. We sincerely appreciate the reviewer's identification of this critical technical detail. We have amended the inaccurate reference to “water samples” in the original text to “stool samples” in accordance with the recommendations of the Global Task Force on Cholera Control (GTFCC) Monitoring Guidelines. We have also adjusted the wording to ensure consistency with current technical practices and international guidelines (pp.33-34):

***Policy Recommendations***

*……*

*(6) Joint prevention and control at transportation hubs: at transportation hubs such as Wuhan Tianhe Airport and Hankou Railway Station, establish symptom monitoring mechanisms for individuals entering Hubei Province. For flights and trains arriving from cholera-endemic areas, implement active symptom-based screening. Conduct rapid diagnostic tests (RDTs) or PCR testing for Vibrio cholerae on stool samples from symptomatic individuals identified during screening, particularly those with diarrhea, and establish a “real-time case information sharing platform” with surrounding provinces to block the transmission chain of imported epidemics.*

Additionally, adhering to the public health principle of “basic measures first, then enhanced measures”, we have moved the original recommendations on drinking water safety (Section 6) and food safety (Section 7) to the forefront of the policy recommendations (Sections 1 and 2). This highlights the fundamental and core role of ensuring safe drinking water as the most essential measure in cholera prevention and control.

**Comment 1-4.**

Result section: The positive total effect for population density is 0.837 and not 0.807 in the text: "(1) Population density significantly increased the level of cholera epidemics and was the most dominant endogenous factor contributing to cholera epidemics, exhibiting a positive total effect (0.807)".

There is a duplication in the text:"The annual annual mean summer temperature in Hubei Province is approximately 27°C, which is close to the temperature suitable for the reproduction of Vibrio cholerae".

**Response 1-4.**

We thank the reviewers for their meticulous proofreading, which helped us correct two important details in the manuscript.

1. Correction regarding the overall effect value of population density. We have verified Table 4 and confirmed that the total effect value (standardized regression coefficient) of population density on cholera incidence is 0.837. We have revised the relevant statement in point (1) of the “Formation Mechanisms of Endogenous Variables in Cholera Epidemics” section under “Results” to read: “*(1) Population density significantly increased the level of cholera epidemics and was the most dominant endogenous factor contributing to cholera epidemics, exhibiting a positive total effect (0.837)”.*

2. Concision of Textual Redundancy. We have adopted the suggestion to remove the redundant “annual” from “The annual annual mean summer temperature in Hubei Province is approximately 27°C”, revising it to “The annual mean summer temperature in Hubei Province is approximately 27°C”, thereby enhancing sentence conciseness and fluency.

**Comment 1-5.**

The long study period, including the particularly insightful silent period and the exploration of factors that may explain it, is highly valuable. Congratulations for that! However, I would suggest focusing the statistical analyses (Mann-Kendall, Wavelet, and SEM) on the most recent 20-year period, while retaining descriptive analyses (which you have presented very well) for the entire 1949-2020 timeframe.

**Response 1-5.**

We sincerely appreciate the reviewers' recognition of this study, particularly their acknowledgment of the analytical value of the “non-epidemic period”. Regarding the suggestion to focus core statistical analysis on the past 20 years of data, we have carefully weighed this proposal. Based on two key considerations, we have decided to maintain the existing analytical framework and wish to clarify our reasoning: First, the fundamental alignment with the study's core objectives. This research aims to systematically reveal long-term historical patterns spanning different stages of socioeconomic development and epidemic control. Applying core analytical methods—such as Mann-Kendall trend tests, wavelet period analysis, and structural equation modeling—exclusively to the recent 20-year period would strip the study of its most critical historical depth and comparative dimension. Second, practical considerations regarding manuscript length and information density. Within the limited scope of this paper, we must strike an optimal balance between analytical depth and breadth. Implementing the suggested approach would necessitate presenting two complete statistical analysis frameworks in parallel: one for descriptive analysis across the entire time span and another for in-depth statistical modeling of the past two decades. This would not only substantially increase the paper's length but also risk dispersing core information and blurring the focus of the argument, thereby undermining the text's coherence and readability. We believe that, within the current framework, using the full-period data to uniformly support both descriptive and inferential analyses represents the optimal choice for ensuring a compact, logically progressive structure that most effectively serves the core research objectives. We once again express our gratitude for the reviewer's valuable feedback, which prompted a crucial re-examination of the unity and conciseness of our research design.

**Reviewer #5**

**Comment 1-1.**

1. The objectives are articulated with greater precision. However, the authors should clearly address how these objectives align with the existing gaps in the literature.

2. The study design is well-organized, using an integrated approach that combines both spatial and temporal analyses. The methodological rigor is also effectively aligned with the research questions posed, which enhances the overall validity and reliability of the manuscript. A clearer statement is needed to elaborate on the rationale behind the chosen methodology and how the research objectives provide insights into the design's robustness.

3. The authors provide a clear and detailed description of the population under study with the characterstic appropriate for the hypothesis being tested. Suggestions for Improvement such as further elaboration of any demographic variations within the population and their potential impact on the results.

4. The study's sample size is a significant strength, being sufficient, meeting convectional standards, and ensuring statistical power. Further discussion addressing how the sample size relates to different variables would add robustness to the conclusions.

5. The statistical analysis used is appropriate and effectively supports the conclusions drawn. To further strengthen the methodological credibility, it would help readers to see a clear rationale for why these particular tests were chosen over others. Including center of gravity analyses, seasonal distribution visuals, and local indicators of spatial autocorrelation would enhance interpretability. Besides, comparing their choice of Structural Equation Modeling (SEM) and its alignment with the conceptual framework to previous studies that have utilized similar frameworks could further strenthen the manuscript's analytical contribution.

6. The authors’ acknowledgment of ethical compliance is significant strength of the manuscript. Further justification on how informed consent and data confidentiality were managed in practice would boost the ethical rigor.

**Response 1-1.**

We sincerely thank the reviewers for their high recognition of our research design and scientific rigor, as well as for their valuable suggestions to enhance the depth and transparency of the paper. We have carefully considered each of your comments and made targeted revisions and additions to the manuscript.

1. Clarifying the alignment of research objectives with gaps in the literature:

Following the articulation of three research questions at the conclusion of the “Introduction”, we have added a new paragraph explicitly stating how this study addresses two gaps in existing research: “long-term, high-resolution geographic analysis” and “comprehensive quantitative mechanism analysis”. This clearly anchors our research objectives to deficiencies in the current literature. The specific revision is as follows (p.4):

***Introduction***

*……To this end, this study integrates 72 years of county-level cholera epidemic records from Hubei Province spanning 1949 to 2020 with multidimensional environmental data. Employing a comprehensive methodology including time series analysis, spatial statistics, and structural equation modeling, it aims to reveal the complex mechanisms underlying regional cholera epidemics through a long-term, high-spatial-resolution geographic analytical framework. It aims to provide scientific insights for cholera prevention and control in river- and lake-rich regions, particularly for other areas globally facing similar environmental conditions (e.g., monsoon climate, dense river networks) and socioeconomic pressures (e.g., rapid urbanization, high population mobility).*

2. We have elaborated on the specific theoretical rationale and relative advantages for selecting each analytical technique in the corresponding descriptions within the “Methods” section. For instance, regarding the M-K test and wavelet analysis, we emphasize their suitability for trend testing and cycle detection in long-term, non-stationary data. For spatial analysis (centroid, LISA), we explain its effectiveness in revealing spatial clustering and evolution processes. For structural equation modeling (SEM), we highlighted its unique capability to handle direct and indirect causal relationships among multiple variables.

3. Potential impact of demographic variations within analyzed populations:

We fully acknowledge the significance of this suggestion. Given that this study employs “county-level” as its macro-level analytical unit, with a primary focus on examining regional environmental and socioeconomic drivers, the analysis of intra-population variations (e.g., age, occupation, immune status) was not incorporated into the current analytical framework. We anticipate future research will explore the integration of individual-level data by leveraging more detailed historical records.

4. Regarding sample size and variable associations: We have expanded the discussion on model robustness in the “Discussion” section to include considerations of sample size (n=103). We explicitly state that this sample size meets the basic requirements for SEM analysis, enabling stable estimation of strong and moderately strong effects (e.g., population density, summer temperature). However, statistical power may be limited for variables with weak effects (e.g., river network density). We have also addressed this as a “research limitation”, with the specific modification as follows (P):

***Research Limitations***

*……*

*(3) Structural Equation Modeling. The sample size for the structural equation model in this study comprised 103 county-level units. While meeting the basic requirements for model fitting, it remains relatively small compared to the generally recommended sample size (typically n>200). This may result in insufficient statistical power for testing variables with weaker effects in the model (such as river network density), while estimates for variables with strong effects (such as population density and summer temperature) remain relatively stable. This limitation partially affects the stability and generalization ability of the model parameters. Future studies utilizing longer time series or higher spatial resolution data could enhance the model's robustness and interpretability.*

5. Result Visualization and Methodological Comparison. Regarding seasonal distribution visualization, we appreciate the reviewer's suggestion to enhance result readability. After careful evaluation, we found that the source data used for seasonal analysis in this study exhibits the following characteristics: a limited number of case entries explicitly labeled with the month of onset are recorded. Plotting monthly distribution maps based on this incomplete, non-representative subset could yield misleading conclusions that fail to accurately reflect the overall seasonal patterns across the province over 72 years. Therefore, we decided against creating separate seasonal distribution maps to avoid potential bias. Instead, within the “Seasonal changes” subsection of the “Results” section, we have clearly reported the annual distribution frequency of cases with seasonal information (number of outbreaks occurring in spring, summer, autumn, and winter) and the proportion of cases by season (percentage of cases in each season) through detailed text and tables. We have also explicitly identified the peak months (August, September, October). We believe this statistically descriptive presentation represents the most accurate and responsible approach to presenting results given the current data limitations. Additionally, spatial autocorrelation local indicators (LISA), local clustering, and outlier analysis plots are presented as core results (Fig. 8b). Centroid analysis and epidemic center migration trajectory maps are presented as core results (Fig. 9f).

Regarding SEM Methodological Comparison: We have added a new paragraph in the “Discussion” section comparing the SEM framework used in this study with commonly employed methods in similar research, such as multiple regression or geoweight regression. We emphasize SEM's core contribution in quantifying indirect effects and revealing the complex interaction pathways among “nature-disaster-socioeconomic” factors, thereby highlighting the mechanistic explanatory strength of our analysis. Specific revisions are as follows (p.31):

***Discussion***

***Research Extension and Comparison***

*……*

*Regarding the influencing factors of cholera epidemics. First, methodologically, the SEM employed in this study offers a novel perspective beyond traditional association analyses for understanding the mechanisms driving regional cholera outbreaks. Most existing research relies on multiple regression or geoweight regression to identify risk factors. While these methods can reveal spatial heterogeneity, they struggle to quantify indirect effects and complex mediating pathways between factors. SEM's decomposition of causal pathways enables a more mechanistic understanding of how these factors influence cholera epidemics.*

**Comment 1-2.**

1. The analyses used in the results section perfectly align well with the pre-established analysis plan demonstrating systematic and rigorous analytical framework. Two targeted improvements would enhance the discussion

a) Explicitly referencing specific sections of the analysis plan when discussing the results.

b) Contrasting the findings with contemporary studies for greater depth.

2. The results are presented with clarity. The Wavelet Variance Plot for Cholera (Fig. 6) is particularly insightful for revealing the dominant temporal cycles in the outbreak data. To fully deliver on the manuscript's promise of analyzing spatio-temporal patterns, the connection between this temporal analysis and the spatial clusters (Fig. 8) should be strengthened.

3. The figures and tables included in the manuscript are of a generally high quality and contribute effectively to the clarity of the results. Fig. 11 could benefit from additional annotations or emphasizing specific data points to draw attention to key findings.

**Response 1-2.**

We appreciate the reviewers' acknowledgment of our results section and analytical framework, as well as their valuable suggestions for enhancing the depth of discussion and presentation of findings. We have implemented the following improvements based on your guidance:

1. Enhanced discussion with improved coherence:

We have revised the “Discussion” section. When analyzing each key finding (e.g., temporal cycles, spatial hotspots), we explicitly reference the corresponding charts in the “Results” section. This enhances the paper's internal logical consistency from “problem formulation” to “analytical validation” to “interpretative discussion”. Specific modifications are as follows (pp.29-31):

***Discussion***

***Research Extension and Comparison***

*Temporal Variations in Cholera Epidemics in Hubei Province. First, the 25-year cholera silent period (1955-1979) identified in this study constitutes one of the salient features of its spatiotemporal distribution pattern (Fig. 3).*

*……*

*In addition, this study identified three significant cholera epidemic cycles of 29, 19, and 8 years, which may reflect the coupling of multi-scale driving processes (Fig. 5).*

*……*

*Regarding the spatial changes of cholera epidemics in Hubei Province. The shift in the epidemic's center of gravity was not merely a result of the simple accumulation of cases; instead, it reflected the transformation of core areas of population and socio-economic activities within the province (Fig. 9f).*

*……*

*Second, the results of the structural equation model (SEM) showed (Tab. 4) that nighttime light exhibited a significant positive total effect on cholera epidemic levels (1.080), yet this finding required cautious interpretation.*

2. Regarding the comparison of this study's findings with similar research. In the “Discussion” section, we have conducted a comprehensive comparative analysis of this study's conclusions against similar research from different regions and time periods. The specific content is presented as follows (P32-33):

*Regarding the comparison of this study’s findings with other research. Fist, the driving mechanisms of cholera in Hubei Province revealed in this study not only align with the general patterns of global cholera epidemics but also exhibit distinct characteristics due to environmental differences between inland water networks and coastal brackish water convergence zones. This study found that the average summer temperature was one of the strongest environmental factors driving cholera epidemics. This finding was highly consistent with observations from major global cholera-endemic regions. In Bangladesh, rising sea surface temperatures had been confirmed as a key driver of seasonal epidemics and interannual outbreaks; in the African Great Lakes region, temperature increases following the rainy season often coincided with epidemic outbreaks. Together, these findings corroborated the core role of temperature as a “universal switch” regulating the proliferation rate of Vibrio cholerae in aquatic environments [17, 21]. However, the uniqueness of this case lied in its purely inland freshwater environment. Unlike coastal and estuarine epidemic areas (such as the Bay of Bengal), which were strongly influenced by salinity gradients and tidal effects, the river and lake networks in Hubei Province exhibited stable and extremely low salinity. Our model indicateed that the direct impact of river network density itself was weaker, suggesting that in freshwater systems, dense water networks provided the necessary medium for transmission but are not a sufficient condition determining epidemic intensity. The spatial differentiation of epidemics was primarily determined by the degree of population aggregation and climatic suitability windows superimposed on this hydrological background. This “hydrological base + socio-climatic amplifier” mechanism provideed a theoretical framework for understanding cholera risks in other inland water-rich regions (such as large lake basins). Second, the transmission pathways of cholera were undergoing a global evolution. The increased records of outbreaks triggered by group gatherings in Hubei Province since the 1980s, as observed in this study, were not an isolated phenomenon. Instead, they reflected a global trend shifting from traditional “waterborne transmission” dominance toward an increasing importance of “foodborne transmission” [54, 56]. As urban and rural water supply and sanitation infrastructure improve, the proportion of outbreaks directly caused by drinking water contamination had declined. However, the lengthening of food supply chains and the frequent occurrence of group dining activities driven by globalization and urbanization had created new pathways for Vibrio cholerae cross-contamination through food. The case of Hubei Province demonstrated that even in inland regions, socio-economic development and lifestyle changes were reshaping the transmission dynamics of infectious diseases. This necessitated that modern cholera prevention and control must transcended a singular environmental sanitation perspective and incorporate systematic supervision of food supply chain safety and collective dining hygiene. In addtion, since 1949, the impact of disaster factors on cholera epidemics had gradually weakened. The root cause of this transformation lied in the progress China had achieved since 1949 in improving medical and health standards, sustaining economic development, and maintaining social stability, which had laid a more solid foundation for cholera prevention and control [27].*

3. Regarding the relationship between temporal analysis and spatial clustering, we fully understand and appreciate your intention to enhance the paper's comprehensiveness by strengthening spatiotemporal connections. After careful consideration, we believe the current structure—presenting temporal trends, spatial patterns, and formation mechanisms as three distinct, progressive chapters—best facilitates readers' step-by-step understanding of this complex issue. We are concerned that integrating spatiotemporal correlations may duplicate the existing, logically coherent structure and dilute the focus on core conclusions. Furthermore, the primary purpose of Figure 11 is to visually demonstrate the long-term covariant trends between key variables and epidemic sequences, not to precisely label individual events. We believe maintaining its current concise form better enables readers to grasp the macro-level, long-term synergistic relationships. We once again appreciate your valuable suggestions, which have prompted crucial reflection on the overall balance of our paper.

**Comment 1-3.**

1. The conclusions drawn in the manuscript are significantly supported by the data presented throughout the study.

2. The limitations of the analysis are addressed, although they could benefit from more detailed discussion of these limitations such as any restrictions arising from sample size, data availability, or methodological choices.

3. The authors effectively discuss how their findings advance the understanding of cholera in Hubei Province. To further strengthen the manuscript, they should also articulate the broader significance of their data for ongoing global cholera research and public health strategies.

4. The manuscript successfully highlights the public health importance of the cholera findings.

**Response 1-3.**

I appreciate the reviewers' affirmation of my research conclusions and their public health significance. I have made significant additions and revisions to the relevant sections based on your suggestions.

1. Fistly, Regarding sample size limitations, We have explicitly stated that the structural equation model (SEM) in this study employs a sample of 103 county-level units. While this meets the basic requirements for model fit, the relatively small sample size may constrain the stability of model parameters and reduce the statistical power for testing weakly effective variables (such as river network density) within the model. This could result in insufficient sensitivity to certain latent but weak associations. Specific revisions are as follows (p.36):

***Research Limitations***

*……*

*(3) Structural Equation Modeling. The sample size for the structural equation model in this study comprised 103 county-level units. While meeting the basic requirements for model fitting, it remains relatively small compared to the generally recommended sample size (typically n>200). This may result in insufficient statistical power for testing variables with weaker effects in the model (such as river network density), while estimates for variables with strong effects (such as population density and summer temperature) remain relatively stable. This limitation partially affects the stability and generalization ability of the model parameters. Future studies utilizing longer time series or higher spatial resolution data could enhance the model's robustness and interpretability.*

Secondly, regarding data availability, we have provided a more detailed breakdown of several key variables that could not be incorporated into the model due to missing historical records. These include water and sanitation coverage, water pH levels, and vaccination rates. I acknowledge that the absence of this data may prevent us from fully assessing the actual effectiveness of sanitation interventions in interrupting transmission routes. Specific revisions are as follows (P 35-36):

***Research Limitations***

*……*

*(2) Environmental factor data. The consideration of factors affecting cholera epidemics was not fully comprehensive. First, water and sanitation facilities (such as safe drinking water coverage and hygienic toilet prevalence) are critical components in interrupting cholera transmission. However, due to the study's long time span (1949-2020) and county-level spatial scale, it was impossible to obtain continuous, standardized quantitative data covering the entire period and all regions. Consequently, these factors could not be incorporated into the model. Second, Hubei Province's healthcare standards (including county-level diagnostic and treatment capabilities and infectious disease prevention systems) represent variables influencing epidemic spread and control. However, pre-2000 data on county-level health conditions (such as rural sanitation facility coverage) and and medical standards (e.g., specialized diagnostic and treatment capabilities for intestinal infectious diseases) prior to 2000 lacked standardized statistical systems. Consequently, quantitative data suitable for long-term, county-level analysis were unavailable, precluding their inclusion in this quantitative model. Additionally, water pH—a critical environmental factor affecting Vibrio cholerae survival and reproduction—could not be analyzed due to the near-total absence of pH monitoring data for county-level rivers and lakes prior to 2000. Similarly, historical constraints limited the availability of sunshine duration data for Hubei Province during its early period (1949-1982), preventing its inclusion in the quantitative analysis of cholera outbreak mechanisms. To address these limitations, future research could supplement historical data on sanitation conditions, medical standards, water pH levels, and meteorological conditions through systematic review of local chronicles' sections on “sanitation” and “epidemic prevention”, retrospective analysis of historical archives from environmental monitoring agencies (e.g., water quality records from hydrological stations), and enhanced mining and interpolation of historical meteorological data. Building upon this foundation, more comprehensive multi-factor quantitative analyses could be conducted to deepen understanding of the driving mechanisms behind cholera epidemics.*

Finally, regarding methodological choices, we have outlined the inherent considerations and trade-offs involved in selecting a cross-sectional structural equation model with time aggregation. This approach aims to capture the “steady-state” drivers influencing long-term spatial patterns, but it also implies that we have not fully captured the dynamic temporal relationships and lagged effects between variables. We have explicitly identified this as a key direction for future research.

2. Regarding the “universal value” of this research. We have specifically addressed the universal implications of our findings for global cholera research and public health strategies in the final paragraph of the Discussion, Research Extension and Comparison section. First, we confirmed the pivotal role of temperature as a universal driver while establishing the “hydrological foundation + socio-climatic amplifier” theoretical framework applicable to inland water network regions, thereby addressing the historical research gap in freshwater environment focus. Second, it confirms the global shift in cholera transmission pathways from waterborne dominance toward increasing foodborne transmission, warning that even in inland regions with improved infrastructure, food supply chain regulation must be integrated into modern prevention and control systems. Third, historical comparisons demonstrate that sustained socioeconomic development and public health system construction form the fundamental basis for mitigating disaster-induced impacts and achieving long-term epidemic control. In summary, this study not only provides regional empirical evidence but also offers an analytical paradigm and strategic perspective with broad reference value for understanding cholera risks across diverse environments, responding to evolving transmission pathways, and planning long-term prevention strategies. Specific findings are outlined as follows(pp.31-32):

***Discussion***

***Research Extension and Comparison***

*……*

*Regarding the comparison of this study’s findings with other research. Fist, the driving mechanisms of cholera in Hubei Province revealed in this study not only align with the general patterns of global cholera epidemics but also exhibit distinct characteristics due to environmental differences between inland water networks and coastal brackish water convergence zones. This study found that the average summer temperature was one of the strongest environmental factors driving cholera epidemics. This finding was highly consistent with observations from major global cholera-endemic regions. In Bangladesh, rising sea surface temperatures had been confirmed as a key driver of seasonal epidemics and interannual outbreaks; in the African Great Lakes region, temperature increases following the rainy season often coincided with epidemic outbreaks. Together, these findings corroborated the core role of temperature as a “universal switch” regulating the proliferation rate of Vibrio cholerae in aquatic environments [17, 21]. However, the uniqueness of this case lied in its purely inland freshwater environment. Unlike coastal and estuarine epidemic areas (such as the Bay of Bengal), which were strongly influenced by salinity gradients and tidal effects, the river and lake networks in Hubei Province exhibited stable and extremely low salinity. Our model indicateed that the direct impact of river network density itself was weaker, suggesting that in freshwater systems, dense water networks provided the necessary medium for transmission but are not a sufficient condition determining epidemic intensity. The spatial differentiation of epidemics was primarily determined by the degree of population aggregation and climatic suitability windows superimposed on this hydrological background. This “hydrological base + socio-climatic amplifier” mechanism provideed a theoretical framework for understanding cholera risks in other inland water-rich regions (such as large lake basins). Second, the transmission pathways of cholera were undergoing a global evolution. The increased records of outbreaks triggered by group gatherings in Hubei Province since the 1980s, as observed in this study, were not an isolated phenomenon. Instead, they reflected a global trend shifting from traditional “waterborne transmission” dominance toward an increasing importance of “foodborne transmission” [54, 56]. As urban and rural water supply and sanitation infrastructure improve, the proportion of outbreaks directly caused by drinking water contamination had declined. However, the lengthening of food supply chains and the frequent occurrence of group dining activities driven by globalization and urbanization had created new pathways for Vibrio cholerae cross-contamination through food. The case of Hubei Province demonstrated that even in inland regions, socio-economic development and lifestyle changes were reshaping the transmission dynamics of infectious diseases. This necessitated that modern cholera prevention and control must transcended a singular environmental sanitation perspective and incorporate systematic supervision of food supply chain safety and collective dining hygiene. In addtion, since 1949, the impact of disaster factors on cholera epidemics had gradually weakened. The root cause of this transformation lied in the progress China had achieved since 1949 in improving medical and health standards, sustaining economic development, and maintaining social stability, which had laid a more solid foundation for cholera prevention and control [27].*

**Comment 1-4.**

Overall Comments

The manuscript provides a substantial investigation into cholera epidemics in Hubei Province, China, offering valuable insights for epidemiology. It employs rigorous methodologies and has practical implications for public health interventions, though several areas need improvement to enhance clarity and impact.

Strengths

Clear Objectives: The study sets well-defined objectives that clarify its significance in understanding cholera patterns.

Robust Methodology: The integration of spatial and temporal analyses allows for a nuanced examination of cholera outbreaks, enhancing validity.

Public Health Relevance: The research emphasizes important implications for policymakers and health practitioners, particularly regarding intervention strategies.

Effective Data Presentation: Results are clearly presented with appropriate figures and tables, aiding reader comprehension.

Weaknesses

Limited Contextualization: There is insufficient comparison with existing literature, which could strengthen the significance of the findings.

Lack of Discussion on Limitations: A more thorough exploration of potential biases and methodological constraints is needed for a balanced perspective.

Ethical Considerations: The manuscript should provide a deeper discussion of ethical standards adhered to, particularly regarding informed consent and data confidentiality.

The study’s unique contribution lies in its comprehensive analysis, offering fresh insights into cholera transmission dynamics critical for effective public health responses.

Additional Minor Comments for the Authors

1. Data Reliability and True Absence of Cases (25 Years): The authors have addressed data reliability effectively by providing a more detailed discussion of data collection methods. However, they should further elaborate on the implications of any potential gaps in case reporting over the past 25 years. Including a more comprehensive description of validation processes would strengthen the credibility of the findings.

2. Variable Selection and Conceptual Framework of Structural Equation Modeling (SEM): The authors have made commendable progress in clarifying aspects of variable selection. Still, they should explicitly align their choices with the conceptual framework of SEM. Additionally, including examples of previous studies that have utilized similar frameworks could enhance the paper’s analytical contribution.

3. Statistical Rigor and Interpretation of Temporal Patterns: While the justification for statistical methods has improved, the authors should also consider providing comparisons with recent methodologies to further substantiate their claims, thereby enriching the discussion on statistical rigor.

4. Spatial Analysis and Presentation: The authors have made strides in improving spatial analysis by incorporating more visual representations. However, they should also consider adding center of gravity analyses, seasonal distribution visuals, and local indicators of spatial autocorrelation. Including these elements would enhance interpretability and provide deeper insights into the patterns observed.

5. The introduction is somewhat longer than necessary. Consider reducing it by approximately 20–30\%. The historical context on cholera epidemics and theoretical background can be condensed to allow the motivation and unique contributions of this study to emerge more clearly.

**Response 1-4.**

We sincerely thank the reviewers for their careful review of our revised manuscript and for their valuable and insightful suggestions aimed at further enhancing the quality of the paper. These comments have prompted us to engage in deeper reflection on the rigor of our research, the focus of our arguments, and the clarity of our presentation. Our specific responses are as follows:

1. Regarding “Data Reliability and the 25-Year Case-Free Period”. We appreciate the reviewer's attention to this matter. The data foundation of this study consists of systematically collected historical documentary records, not data generated by modern active surveillance systems. We cannot perform data validation procedures or vulnerability analyses as we would with contemporary data, as this falls outside the scope of historical data research. Regarding the “1955-1979 case-free period,” we have thoroughly addressed the differences in surveillance systems across periods in the “Data Quality and Limitations” section (pp.10-11), explicitly stating: The early (1949-1979) surveillance system was still in its formative stages, resulting in high underreporting rates... The “low incidence period” or “disease-free period” reflects, to some extent, the limitations of surveillance capabilities during that era." This candid explanation represents the most appropriate approach to addressing the reliability of historical data. In the “Discussion” section (pp.28-29), we have interpreted this period as “a phased outcome resulting from the combined effects of administrative intervention and a relatively closed social system under specific historical conditions”, rather than simplistically attributing it to an absolute absence of disease. This approach maximizes the credibility and depth of our interpretation for this period. We once again appreciate your valuable suggestions.

2. Regarding “SEM Variable Selection and Theoretical Framework”, we have supplemented the description of variable selection in the “Data source” section (p.8) to explicitly highlight alignment with the theoretical framework and cite a comparable study as supporting evidence. The specific revision is as follows:

*The selection of the aforementioned variables strictly adheres to the theoretical framework of “source of infection-mode of transmission-susceptible population” illustrated in Figure 2. It aims to characterize the core components of this classic epidemiological chain through quantifiable proxy variables [[27](../csc材料/CV and Related Academic Achievements for CSC Joint PhD Program-Chen Zhiyu/Related Academic Achievements/1850—1949年湖北省...乱流行的时空规律及影响机制.pdf)].*

3. We appreciate the reviewers' suggestions. In the “Methods” section (p.11-15), we have elaborated in detail on the rationale for selecting the M-K test, wavelet analysis, and SEM, emphasizing their applicability and robustness for analyzing long-term, non-stationary time series data and multi-variant mechanisms in this study. Compared to some more complex, data-intensive recent time-series models, our chosen combination of methods offers clear theoretical foundations and intuitive result interpretation for long-term trend testing, cycle identification, and cross-sectional causal path analysis. This aligns better with our core objective of “describing long-term patterns and analyzing steady-state driving mechanisms”. We also addressed this in the research limitations section(p.36): “*Although presented as exploratory analysis, Figure 11 displays standardized time series of key variables alongside cholera cases, visualizing their covariate trends and potential lagged relationships. However, rigorous validation of time lags, analysis of non-stationary series, and construction of spatiotemporal panel models require more complex econometric methods applied to more complete, higher-frequency panel data—representing a key direction for future research.”*

4. Regarding “Spatial Analysis and Presentation”. First, the results and visualizations of the center-of-gravity analysis are included in the “Results, Spatial Distribution Changes” section and clearly presented in Figure 9f (p.21). Second, the spatial autocorrelation local indicators. The results and visualizations of the Localized Isotropic and Anomaly Analysis (LISA) are included in the “Results, Overall Distribution Characteristics” section and clearly presented in Figure 8b (pp.19-20). We have referenced and interpreted these figures in the relevant main text. Finally, regarding seasonal distribution visualization: as stated in our previous response, due to incomplete and non-representative monthly information in historical case records, we opted to present seasonal distribution results in detailed textual form to avoid misrepresentation. We believe this constitutes the most accurate and responsible presentation method currently available.

5. Regarding the streamlining of the introduction section. We consolidated redundant background paragraphs and strengthened the logical progression from research background to research gaps and then to research questions. The revised introduction effectively reduces its length while preserving core scientific information, focusing more intently on highlighting the study's innovations and key contributions.
